# IL1RAP expression and the enrichment of IL‐33 activation signatures in severe neutrophilic asthma

**DOI:** 10.1111/all.15487

**Published:** 2022-08-28

**Authors:** Yusef Eamon Badi, Barbora Salcman, Adam Taylor, Batika Rana, Nazanin Zounemat Kermani, John H. Riley, Sally Worsley, Sharon Mumby, Sven‐Eric Dahlen, David Cousins, Silvia Bulfone‐Paus, Karen Affleck, Kian Fan Chung, Stewart Bates, Ian M. Adcock

**Affiliations:** ^1^ National Heart and Lung Institute, Imperial College London London UK; ^2^ Data Science Institute, Imperial College London London UK; ^3^ School of Biological Sciences, University of Manchester Manchester UK; ^4^ GSK Respiratory Therapeutic Area Unit Stevenage UK; ^5^ Orchard Therapeutics London UK; ^6^ GSK Value Evidence and Outcomes, GSK House Brentford UK; ^7^ Institute of Environmental Medicine, Karolinska Institute Stockholm Sweden; ^8^ Department of Respiratory Sciences University of Leicester Leicester UK; ^9^ GSK Adaptive Immunity Research Unit Stevenage UK; ^10^ BenevolentAI London UK

**Keywords:** gene set variation analysis, IL‐33, severe asthma

## Abstract

**Background:**

Interleukin (IL)‐33 is an upstream regulator of type 2 (T2) eosinophilic inflammation and has been proposed as a key driver of some asthma phenotypes.

**Objective:**

To derive gene signatures from in vitro studies of IL‐33‐stimulated cells and use these to determine IL‐33‐associated enrichment patterns in asthma.

**Methods:**

Signatures downstream of IL‐33 stimulation were derived from our in vitro study of human mast cells and from public datasets of in vitro stimulated human basophils, type 2 innate lymphoid cells (ILC2), regulatory T cells (Treg) and endothelial cells. Gene Set Variation Analysis (GSVA) was used to probe U‐BIOPRED and ADEPT sputum transcriptomics to determine enrichment scores (ES) for each signature according to asthma severity, sputum granulocyte status and previously defined molecular phenotypes.

**Results:**

IL‐33‐activated gene signatures were cell‐specific with little gene overlap. Individual signatures, however, were associated with similar signalling pathways (TNF, NF‐κB, IL‐17 and JAK/STAT signalling) and immune cell differentiation pathways (Th17, Th1 and Th2 differentiation). ES for IL‐33‐activated gene signatures were significantly enriched in asthmatic sputum, particularly in patients with neutrophilic and mixed granulocytic phenotypes. IL‐33 mRNA expression was not elevated in asthma whereas the expression of mRNA for IL1RL1, the IL‐33 receptor, was up‐regulated in the sputum of severe eosinophilic asthma. The mRNA expression for IL1RAP, the IL1RL1 co‐receptor, was greatest in severe neutrophilic and mixed granulocytic asthma.

**Conclusions:**

IL‐33‐activated gene signatures are elevated in neutrophilic and mixed granulocytic asthma corresponding with IL1RAP co‐receptor expression. This suggests incorporating T2‐low asthma in anti‐IL‐33 trials.

## Graphical abstract

Stimulation of human mast cells, basophils, ILC2 cells and HUVECs with IL‐33 generates distinct gene expression patterns but similar pathways are activated. We produced IL‐33‐activated gene signatures and used GSVA and showed enrichment of these signatures in patients with neutrophilic and mixed granulocytic asthma. These patients had the highest expression of the IL‐33 receptor co‐receptor IL1RAP.Abbreviations: BAS, basophil; HUVECs, human umbilical vein endothelial cells; IL, interleukin; ILC2, type 2 innate lymphoid cell; IL1RAP, IL‐1 receptor accessory protein; JAK, Janus kinase; MC, mast cell; STAT, signal transducer and activator of transcription; Th, T helper; TNF, tumor necrosis factor

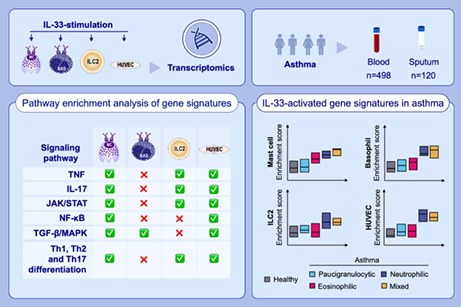

**Abbreviations**
ADEPT: Airway Disease Endotyping for Personalized Therapeutics
DEGs: Differentially expressed genes
ES: Enrichment score
FC: Fold‐change
FDR: False discovery rate
FeNO: Fractional exhaled nitric oxide
GSVA: Gene Set Variation Analysis
HC: Healthy control
HUVEC: Human umbilical vein endothelial cells
IL: Interleukin
ILC2: type 2 innate lymphoid cell
JAK/STAT: Janus kinase/signal transducer and activator of transcription
LS: Lesional
MMA: Mild–moderate asthma
NF‐κB: Nuclear factor‐κB
PNR: Potential non‐responder
PR: Potential responder
SA: Severe asthma
SAs/ex: Severe asthmatic smoker/ex‐smoker
T2: Type 2
TAC: Transcriptome‐Associated Cluster
TGF‐β/MAPK: Transforming growth factor‐β/mitogen‐activated protein kinases
U‐BIOPRED: Unbiased Biomarkers for the Prediction of Respiratory Disease Outcomes

## 1 INTRODUCTION

Asthma is a heterogenous disease characterized by chronic airway inflammation affecting over 350 million people globally. Asthma exhibits many clinical phenotypes which remain poorly understood apart from the severe eosinophilic phenotype characterized by recurrent exacerbations and underlined by a type 2 inflammation with the expression of interleukin (IL)‐4, IL‐13 and IL‐5.^1^ Phenotypes associated with non‐T2 pathways remain unclear although there is evidence for a neutrophilic inflammatory component of asthma that may be driven by Th‐17 and Th1 pathways.^2^ Current research, particularly in severe asthma (SA), aims to identify disease‐driving mechanisms that would constitute a molecular phenotype or endotype of non‐T2 pathways and, thereby enable the discovery of targeted therapies.

Interleukin (IL)‐33 is an alarmin released from airway epithelial cells in response to cellular injury or stress resulting from infection, allergy or pollutants.^3^ Upon release, IL‐33 binds to a heteromeric receptor consisting of the IL‐1 receptor‐like 1, IL1RL1, or ST2 and its co‐receptor IL‐1 receptor accessory protein (IL1RAP). This results in activation of downstream signalling pathways such as nuclear factor‐κB (NF‐κB) and mitogen‐activated protein kinase (MAPK).^3^

IL‐33 activation is associated with type 2 (T2) inflammation and the release of the T2 cytokines, IL‐5 and IL‐13, from T‐helper 2 (Th2) and group 2 innate lymphoid cells (ILC2s). IL‐33 is also involved in the maturation of Th2 cells and acts as a Th2 cell chemoattractant.^4^, ^5^ IL‐33 can also activate mast cells, basophils, eosinophils and natural killer cells.^6^ However, during viral infection, IL‐33 can both drive^3^ and dampen^7^ type 1 immunity. IL1RL1 is constitutively expressed in Th2 cells and transiently expressed in virus‐exposed Th1 cells in vitro and in vivo.^8^ Furthermore, free IL‐33 binds to CD8 + T cells to potentiate cytotoxic functions in the presence of Th1 inflammation.^9^

Associations between *IL‐33* and *IL1RL1* genetic variants have been reported for asthma.^10^ An *IL‐33* loss‐of‐function mutation protects children from asthma and has been associated with a lower blood eosinophil count.^11^ IL‐33 mRNA and protein expression is elevated in the airways of SA patients,^12^ with a greater release of IL‐33 in vivo during rhinovirus‐induced asthma exacerbations.^13^ IL1RL1 is characteristically expressed on Th2 cells which, in part, distinguishes them from Th1 cells^10^ and was one of 20 genes distinguishing eosinophilic from non‐eosinophilic asthma in sputum.^14^ IL1RL1 is also a feature of patients enriched for the molecular phenotype transcriptomic associated cluster (TAC)1 that is associated with very high sputum eosinophilia in SA.^14^ However, drugs directed against the IL‐33/IL1RL1 axis have proved less efficacious in SA patients with high blood eosinophil counts than in those with low blood eosinophils.^15^, ^16^

In order to elucidate the type of asthma driven by IL‐33 and its interaction with its heteromeric receptor, we obtained and derived several cell‐specific IL‐33‐activated gene signatures. We used these to determine their expression levels in the transcriptome of cell compartments in blood and in the airways of asthmatic participants in the Unbiased Biomarkers for the Prediction of Respiratory Disease Outcomes (U‐BIOPRED) cohort. Using this approach, we describe the phenotypes driven by IL‐33, which consisted of the severe neutrophilic and mixed granulocytic asthma.

## 2 METHODS

### 2.1 Patient cohorts

The U‐BIOPRED cohort comprises subjects with severe smoking/ex‐smoking (SAs/ex) (*n* = 23) and severe non‐smoking (SAns) (*n* = 61) asthma in addition to mild–moderate asthma (MMA) (n = 20) and healthy controls (HCs) (*n* = 16).^17^ We analysed the dataset that included clinical data and sputum transcriptomic data for sputum, blood, bronchial and nasal brushings and bronchial biopsies. Patients were compared by asthma severity (MMA, SAs/ex, SAns), by granulocytic subtype (P = Paucigranulocytic, E = Eosinophilic, N = Neutrophilic and M = Mixed) and by molecular phenotype or TAC where TAC1 is high sputum eosinophilic, TAC2 is neutrophilic associated with inflammasome activation and TAC3 is paucigranulocytic or mixed phenotype linked with metabolic/mitochondrial pathways.^14^ The TAC groups were derived from clustering of differentially expressed genes in sputum from asthmatic subjects with eosinophilic versus non‐eosinophilic disease.^14^ The ADEPT asthma cohort,^18^ including severe (SA, *n* = 51), moderate (ModA, *n* = 55) and mild (MldA, *n* = 52) asthma, was used as a validation cohort.

### 2.2 Obtaining public IL‐33 cell stimulation data

A literature search was undertaken to identify cells expressing IL1RL1 which would respond to IL‐33 stimulation. Additionally, we analysed the log_2_ fragments per kilobase of exon per million mapped fragments (fpkm) data of IL1RL1 in different cell types using the gene expression omnibus (GEO).^19^

Th1 cells express IL1RL1 transiently^8^ whilst T‐regulatory (Treg) cells, innate lymphoid cells type 2 (ILC2), Th2 cells, CD8+ T cells, B cells, NK, iNKT and endothelial cells also express IL1RL1.^20^, ^21^ Mast cells, Tregs, eosinophils and basophils were identified as highly expressing IL1RL1 using a log_2_ fpkm cut‐off of 5.

Transcriptomic data were available from the NCBI GEO database^19^ for ILC2 cells cultured with IL‐2/IL‐7 in the presence or absence of IL‐33 (50 ng/ml for 7 days, GSE72433), IL‐33‐stimulated basophils (20 ng/ml for 24 h, GSE64639),^22^ IL‐33 treated human umbilical vein endothelial cells (HUVECs) (50 ng/ml for 4 h, GSE37624)^23^ and Treg cells engineered to constitutively express IL1RL1 and stimulated with IL‐2 and anti‐CD3/CD28 in the presence and absence of 20 ng/ml IL‐33 for 16h (GSE117481).^24^

### 2.3 In vitro IL‐33 stimulation of mast cells

Human peripheral blood mast cells from 6 donors were isolated as previously described incorporating a CD117 magnetic bead purification step.^25^ All patients gave written informed consent, and the protocol was approved by the University of Manchester research ethics committee (UREC 2018–2696‐5711). After 8 weeks, mature primary human mast cells were cultured at 0.5‐1 × 10^6^ in 500 ml supplemented Iscove's Modified Dulbecco's Medium^25^ (see Appendix S1) in the presence or absence of IL‐33 (50 ng/ml) for 24 h. RNA‐seq data were generated using Illumina HiSeq 4000 as previously described.^26^

### 2.4 Development of IL‐33 activated gene signatures

STAR,^27^ Kallisto^28^ and featureCounts^29^ were used in in DNAnexus® for processing RNA‐seq data generated in‐house using IL‐33‐stimulated mast cells. R 3.5.0 (http://www.r‐project.org/) was used to perform statistical analysis. Differential gene expression (DEG) analysis between baseline and treated in vitro cell transcriptomics was performed using Limma for public array datasets and DESeq2 for the mast cell RNA‐seq data.^30^ Up‐ and down‐regulated DEGs were identified by a log_2_ fold change ≥1 or ≤ −1 and a false discovery rate^31^ adjusted *p* value of ≤0.05.

Gene signatures were cross‐checked against published single‐cell data for cell‐specific genes for activated endothelium and mast cells in healthy subjects and patients with mild asthma (Table S1A).^32^ In addition, we compared human neutrophil signatures from healthy donors (Table S1B)^33^ and human eosinophil signatures from both healthy subjects (Shridar eosinophil 2) and patients with asthma (Table S1C).^34^ Any overlap was removed. The final list of gene signatures used can be seen in Table S1. Removed genes are indicated in red.

### 2.5 Identifying a consensus IL‐33‐activated gene signature

Overlap between signatures was investigated to attempt to establish a consensus IL‐33‐activated gene signature. A Venn diagram of all gene signatures was generated using public software (http://bioinformatics.psb.ugent.be/webtools/Venn/). All differential genes seen in at least *n* = 2 experiments were pooled together and analysed in enrichment analysis to attempt to identify any common features across cell types following IL‐33 stimulation.

### 2.6 Statistical and enrichment analysis

Enrichr^35^ was used to perform pathway enrichment analysis of gene signatures in the Kyoto Encyclopedia of Genes and Genomes (KEGG) human gene set database.^36^ Gene set variation analysis (GSVA) was used to determine patient‐wise enrichment scores (ES) indicating the relative collective expression of genes within the gene signatures for patients relative to the rest of the cohort of patients in a given transcriptomics dataset.^37^ The ggpubr package (https://CRAN.R‐project.org/package=ggpubr) was used to perform Wilcoxon rank‐sum tests to establish significance of individual gene differences between groups of subjects and display statistical comparisons on plots with Hommel multiple testing p value adjustment.^38^ Plots were generated using ggplot2 (https://ggplot2.tidyverse.org).

## 3 RESULTS

### 3.1 Generation of IL‐33‐activated gene signatures

IL‐33‐activated gene signatures were generated successfully from mast cell, basophil, ILC2 and HUVECs (Table S2). No differential gene signature was produced from the Tregs analysis. Minimal cell‐specific genes from mast cells, activated endothelium and neutrophils were present in the IL‐33‐activated gene signatures generated (Table S1).

### 3.2 Cell‐specific up‐regulated IL‐33‐activated gene signatures

After filtering out cell‐specific genes, IL‐33‐up‐regulated gene signatures for mast cells (418 genes), basophils (197 genes), ILC2 cells (107 genes) and HUVECs (238 genes) were obtained (Table S2).

Pathway analysis of each cell‐specific IL‐33‐activated up‐regulated gene signature (Tables S3‐6) revealed extensive enrichment of various signalling pathways in all signatures apart from the basophil‐derived signature. These pathways included enrichment in TNF, IL‐17 and JAK/STAT signalling pathways and for Th17 cell, Th1 and Th2 cell differentiation pathways in the mast cell, ILC2 and HUVEC signatures. The NF‐κB signalling pathway was significantly enriched in mast cells and HUVECs but not in either basophil or ILC2 signature. The TGF‐β and MAPK signalling pathways were enriched in all but the ILC2 signature. Pathways associated with asthma were enriched in the mast cell and ILC2 gene signatures whilst pathways associated with inflammatory bowel disease were enriched in all signatures. In addition, rheumatoid arthritis‐associated pathways were enriched in all but the basophil signature.

**FIGURE 1:**
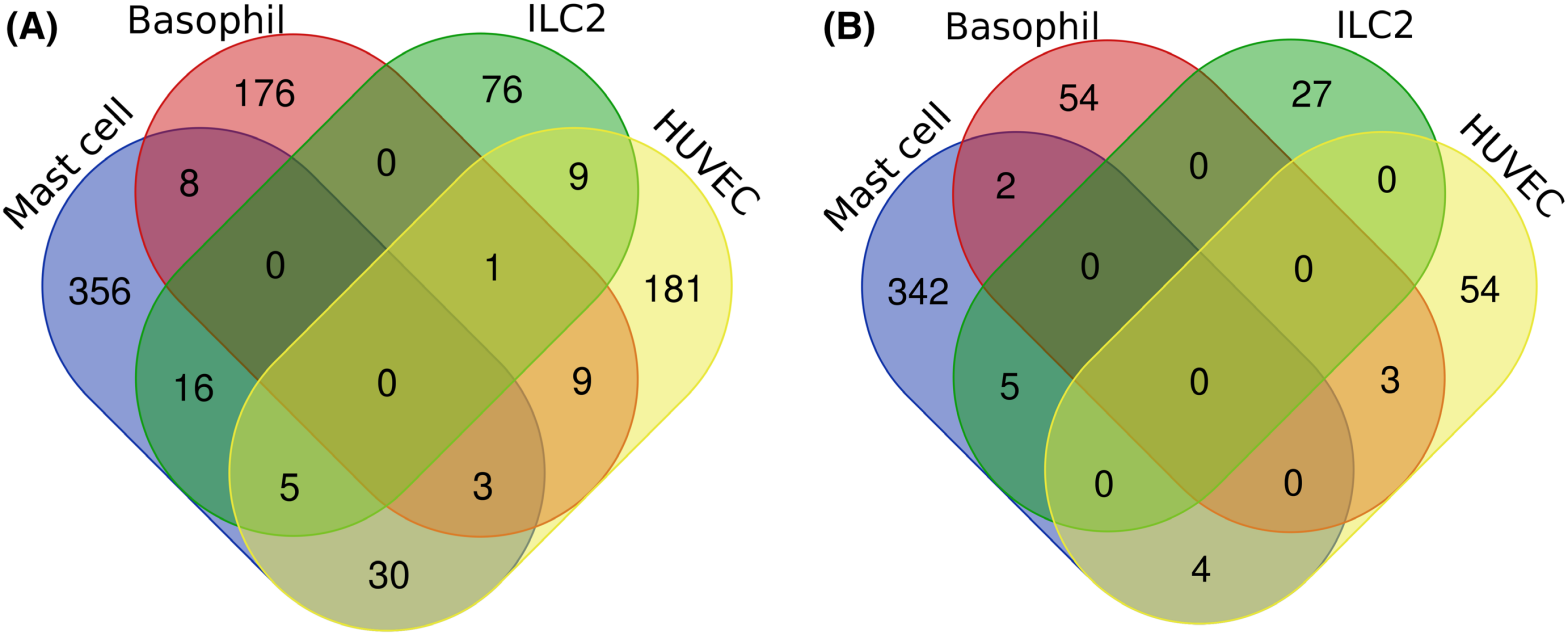
Venn diagram plot of the different cell type generated IL‐33 signatures for (A) up‐regulated (B) and down‐regulated signatures. No consensus overlap is seen; however, there is noted overlap between pairs of signatures

Although the cell‐derived IL‐33‐activated gene signatures overlapped with respect to pathway enrichment, the gene lists were highly cell‐specific with minimal overlap between them (Table S7) and we were unable to identify a consensus IL‐33‐up‐regulated gene signature (Figure 1A). However, an integrative analysis of the overlapping genes within the signatures identified 81 genes that were present in 2 or more signatures (Table S8 A). Pathway analysis of these 81 genes confirmed enrichment of TNF, NF‐κB, IL‐17, JAK/STAT signalling pathways and Th17, Th1 and Th2 differentiation pathways in addition to pathways associated with rheumatoid arthritis and inflammatory bowel disease (Table S9).

### 3.3 Cell‐specific down‐regulated IL‐33‐activated gene signatures

As with the up‐regulated signatures, minimal overlap was seen in the down‐regulated IL‐33‐activated gene signatures with no defined consensus signature (Figure 1B). Only 14 genes (HK3, MS4A3, MATK, ZBTB16, ATP1B1, SCN2A, TP53I11, GPR162, GFOD1, TXNIP, SMAD6, SLC45A3, KLF2, E2F2) had an overlap in 2 or more gene signatures (Table S8B). Pathway enrichment analysis of these 14 genes did not reveal any significant pathways (Table S10). No further analysis of these down‐regulated gene signatures was performed.

### 3.4 Enrichment of IL‐33‐up‐regulated gene signatures in the blood and sputum transcriptome

**FIGURE 2:**
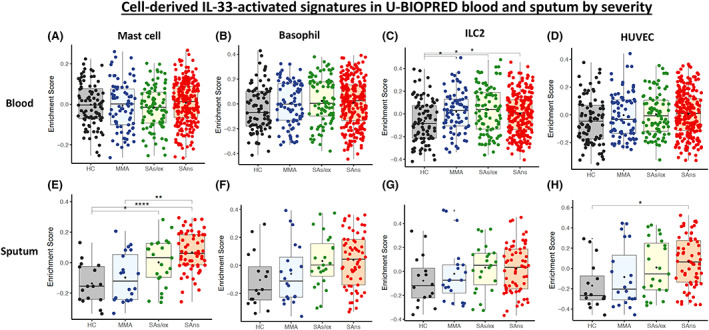
Gene Set Variation Analysis (GSVA) boxplots of U‐BIOPRED blood asthma enrichment scores by severity cohort (HC = healthy control; MMA = mild–moderate asthma; SAs/ex = severe asthma smoking/ex‐smoking; and SAns = severe asthma non‐smoking) for the mast cell, basophil, ILC2 and HUVEC cell‐derived IL‐33‐activated up‐regulated gene signatures for blood (A‐D) and sputum (E‐H)

There was a trend for higher ES in peripheral blood for IL‐33‐up‐regulated gene signatures according to asthma severity (Figure 2A‐D) that reached significance (*p* < 0.05) for the ILC2 signature for all asthma cohorts against HC. There was no significant enrichment in blood by either granulocytic or TAC subtype.

**FIGURE 3:**
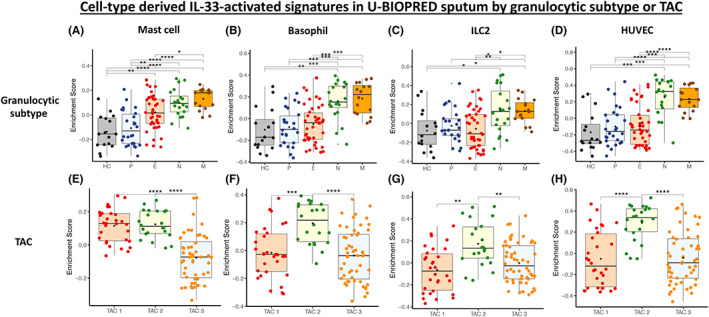
Gene Set Variation Analysis (GSVA) boxplots of U‐BIOPRED sputum enrichment scores for the mast cell, basophil, ILC2 and HUVEC cell‐derived IL‐33‐activated up‐regulated gene signatures by granulocytic subtype (HC = healthy control, P = paucigranulocytic, E = eosinophilic, N = neutrophilic and M = mixed) (A‐D) and transcriptome associated cluster (TAC) which reflects eosinophilic and non‐eosinophilic phenotypes of asthma (TAC1 = eosinophilic asthma, TAC2 = neutrophilic asthma, TAC3 = mixed or paucigranulocytic asthma) (E‐H). These TAC subtypes are novel asthma phenotypes which were derived from clustering performed on differential gene expression analysis between eosinophilic and non‐eosinophilic U‐BIOPRED asthmatic sputum transcriptomics

The ES for IL‐33‐up‐regulated signatures also showed enrichment with asthma severity in sputum (Figure 2E‐H) with significant enrichment for SAns (*p* < 0.0001) and MMA (*p* < 0.01) compared with HC for the IL‐33 mast cell signature (Figure 2E). Significant enrichment was seen in SAns against HC (*p* < 0.05) for the HUVEC IL‐33‐up‐regulated signature (Figure 2H). The ES in sputum were highly significantly elevated for all signatures in neutrophilic and mixed asthma subtypes (Figure 3A‐D). The mast cell signature was also significantly enriched (*p* < 0.01) in eosinophilic asthma compared to HC (Figure 3A).

Furthermore, the TAC1 and TAC2 subtypes, previously derived unbiased clustering of differentially expressed genes between sputum eosinophilic and non‐eosinophilic asthmatics,^14^ were both significantly enriched in comparison with TAC3 (*p* < 0.0001) for the mast cell signature (Figure 3E). Basophil, ILC2 and HUVEC IL‐33‐up‐regulated signatures were significantly enriched in the neutrophilic TAC2 asthma subjects (all *p* < 0.01)(Figure 3F‐H). This was not explained by overlapping genes between the IL‐33‐up‐regulated and TAC gene signatures (Table S7).

There was no significant enrichment of any IL‐33‐activated signatures in bronchial and nasal brushings or bronchial biopsies when analysed according to asthma severity or granulocyte phenotype (Figures S1).

There was a significant positive correlation with sputum neutrophil absolute count and percentages (rho 0.45–0.67) for all cell‐specific IL‐33‐up‐regulated gene signatures. Additionally, epithelial CD3+ and CD8+ and submucosal CD3+ and CD4+ staining^39^ positively correlated (rho 0.43–0.57) with cell‐type signature ESs (Tables S11‐14). A negative correlation with sputum eosinophils was seen in all but the mast cell‐derived signature. Interestingly, serum IL17A positively correlated with all but the basophil‐derived signature. A negative correlation with severity indicators, such as the AQLQ questionnaire and spirometry (FVC and FEV1), was seen for all signatures indicating that a higher IL‐33‐activated ES is associated with poorer asthma control and quality of life.

Analysis of the most versus least enriched (upper versus lower tertiles) U‐BIOPRED asthma patients for each of the IL‐33 signatures in sputum does not reveal any association with age, body mass index (BMI) or sex. No association with LABA or OCS use was found for any of the signatures except for the mast cell signature where OCS use was significantly elevated prior to p value adjustment (most enriched group OCS use *n* = 15/35 versus least enriched group OCS use *n* = 5/34, *p* = 0.0161); however, after adjustment, this is no longer significant (*p* = 0.0569).

### 3.5 Validation of IL‐33‐activated gene signatures in the ADEPT cohort

**FIGURE 4:**
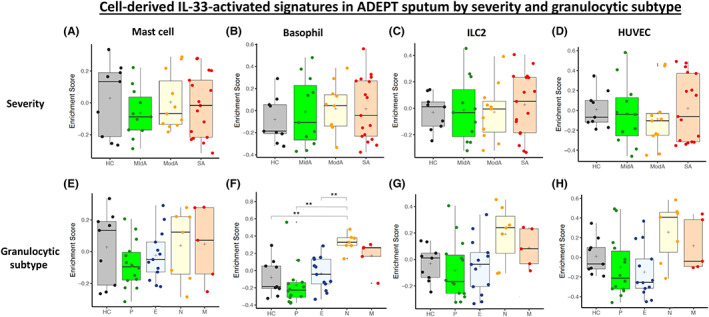
Gene Set Variation Analysis (GSVA) boxplots of ADEPT sputum asthma enrichment scores for the mast cell, basophil, ILC2 and HUVEC cell‐derived IL‐33‐activated up‐regulated gene signatures by severity cohort (SA = severe asthma; ModA = moderate asthma; MldA = mild asthma; and HC = healthy control) (A‐D) and granulocytic subtype (HC = healthy control, P = paucigranulocytic, E = eosinophilic, N = neutrophilic, and M = mixed) (E‐H)

There was no significant difference in ES of cell‐specific IL‐33 signatures by asthma severity across the ADEPT cohort (Figure 4A‐D). However, there was a trend for elevated ES in neutrophilic and mixed subjects in comparison with other inflammatory phenotypes for the basophil, ILC2 and HUVEC signatures, with significance reached in the basophil signature analysis for neutrophilic subjects against all other groups (*p* < 0.01) except the mixed granulocyte group (Figure 4F‐H).

### 3.6 IL‐33 and IL‐33 receptor‐associated mRNA expression asthma

**FIGURE 5:**
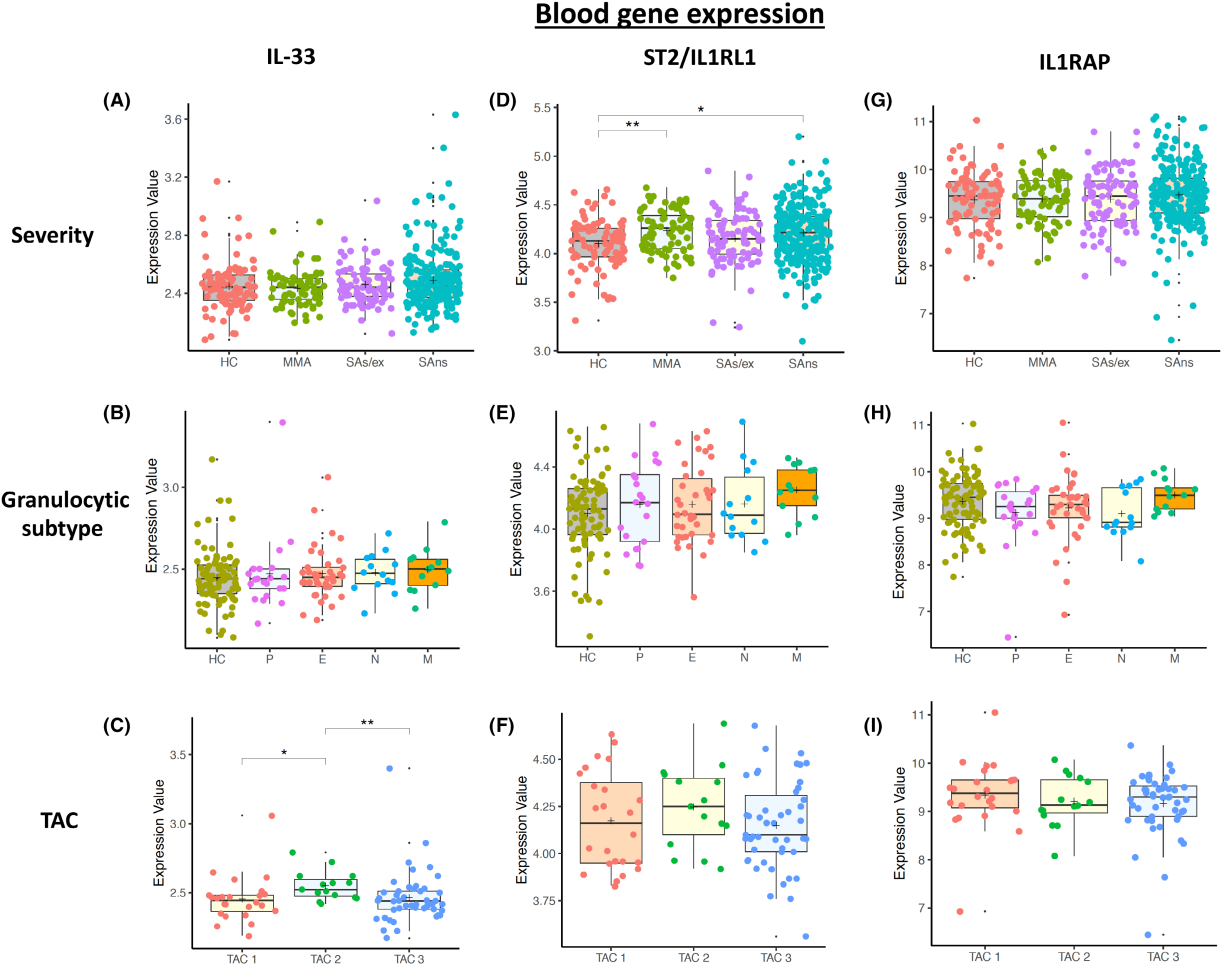
Boxplots of U‐BIOPRED blood asthma gene expression for IL‐33, IL1RL1 (ST2) and IL1RAP (co‐dimer of the IL‐33 receptor) by severity cohort (HC = healthy control; MMA = mild–moderate asthma; SAs/ex = severe asthma smoking/ex‐smoking; and SAns = severe asthma non‐smoking) (A, D, G), granulocytic subtype (HC = Healthy Control, P = Paucigranulocytic, E = Eosinophilic, N = Neutrophilic and M = Mixed) (B, E, H), and transcriptome associated cluster (C, F, I) which reflects eosinophilic and non‐eosinophilic phenotypes of asthma (TAC1 = eosinophilic asthma, TAC2 = neutrophilic asthma and TAC3 = mixed or paucigranulocytic asthma)

**FIGURE 6:**
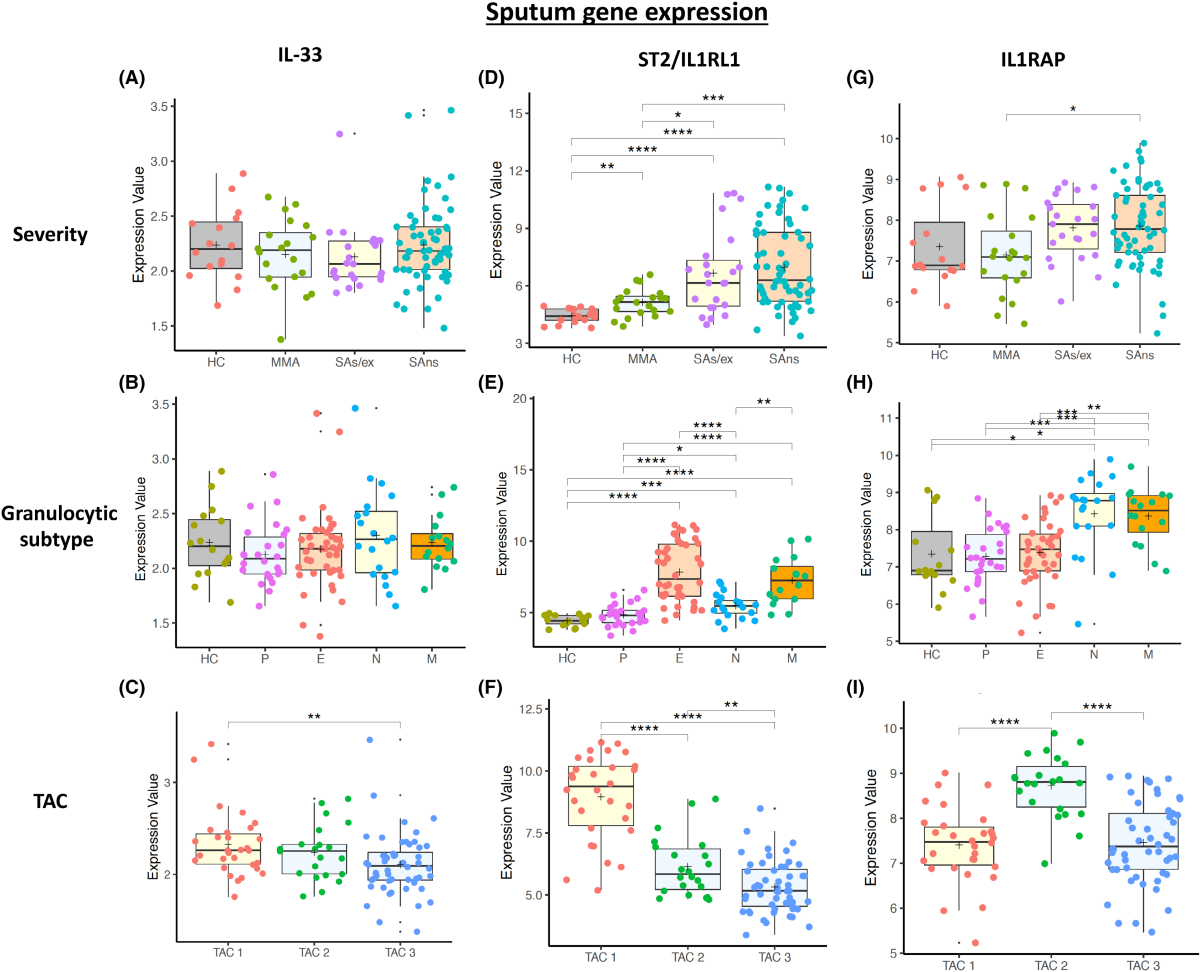
Boxplots of U‐BIOPRED sputum asthma gene expression for IL‐33, IL1RL1 (ST2) and IL1RAP (co‐dimer of the IL‐33 receptor) by severity cohort (HC = healthy control; MMA = mild–moderate asthma; SAs/ex = severe asthma smoking/ex‐smoking; SAns = severe asthma non‐smoking) (A, D, G), granulocytic subtype (HC = healthy control, P = paucigranulocytic, E = eosinophilic, N = neutrophilic and M = mixed) (B, E, H), and transcriptome associated cluster (C, F, I) which reflects eosinophilic and non‐eosinophilic phenotypes of asthma (TAC1 = eosinophilic asthma, TAC2 = neutrophilic asthma and TAC3 = mixed or paucigranulocytic asthma)

IL‐33, IL1RL1 (ST2) and IL1RAP mRNA expression was determined in blood (Figure 5A‐I), sputum (Figure 6A‐I), bronchial biopsies (Figure S4A‐I) and bronchial (Figure S5A‐I) and nasal brushings (Figure S6A‐I).

IL‐33 mRNA levels were generally very low in blood (Figure 5A‐C) and sputum (Figure 6A‐C) in comparison with bronchial biopsies (Figure S4A‐C), bronchial brushings (Figure S5A‐C) and nasal brushings (Figure S6A‐C). Asthma severity and granulocyte status did not alter IL‐33 mRNA expression except in bronchial brushings where it was significantly suppressed (*p* < 0.01) with respect to HC. IL‐33 mRNA expression was significantly (*p* < 0.05) higher in TAC2 patients in blood compared to other TACs (Figure 5C) but higher in sputum TAC1 compared to TAC3 (Figure 6C).

### 3.7 IL1RL1 (ST2) gene expression

There was a wide spread of IL1RL1 mRNA expression in blood that was significantly elevated in peripheral blood cells of MMA (*p* < 0.001) and SAns (*p* < 0.05) compared with HC (Figure 5D). There was no difference in IL1RL1 mRNA expression across asthma severity which agreed with the blood eosinophil levels previously reported in these patients.^17^

Sputum IL1RL1 mRNA levels were significantly higher in SA compared with HC (*p* < 0.001) and MMA (*p* < 0.0001)(Figure 6D). IL1RL1 was significantly elevated in eosinophilic and mixed subtypes (both *p* < 0.0001) compared with paucigranulocytic and neutrophilic asthma subtypes and against HC subjects (Figure 6E). IL1RL1 mRNA was also elevated in neutrophilic asthma when compared to HC (Figure 6E). IL1RL1 mRNA expression was significantly elevated in TAC1 against TAC2 and 3 (*p* < 0.0001)(Figure 6F).

However, IL1RL1 mRNA levels were significantly lower in SAs/ex compared with SAns and MMA and HC (all *p* < 0.05) in bronchial biopsies (Figure S4D). No significant difference in IL1RL1 mRNA expression was seen by granulocyte (Figure S4E) or TAC (Figure S4F) status.

### 3.8 IL1RAP gene expression

IL1RAP mRNA expression in blood was unaffected by asthma severity, granulocytic grouping or TAC subtype (Figure 5G‐I). In contrast, IL1RAP mRNA expression was significantly increased in sputum in SAns and MMA (both *p* < 0.05) compared with HC (Figure 6G). Furthermore, IL1RAP mRNA levels were significantly elevated in neutrophilic and mixed asthma compared to HC (both *p* < 0.001)(Figure 6H) and in TAC2 asthmatics compared with TAC1 and TAC3 asthmatics (both *p* < 0.0001)(Figure 6I).

IL1RAP mRNA expression was unaffected by asthma severity, granulocytic or TAC status in bronchial biopsies (Figure S4G‐I) or in nasal brushings (Figure S6G‐I). IL1RAP mRNA was significantly down‐regulated in bronchial brushings in SA compared to HC (*p* < 0.05; Figure S5G).

The expression of IL‐33, IL‐1RL1 and IL1RAP in the blood and sputum was related to cell counts and treatment (Figures S7‐S11 and Table S15). There was a significant increase in IL1RL1 (p = 9.0 × 10^−7^) and IL1RAP (p = 0.033) mRNAs in sputum in patients prescribed OCS therapy whilst LABA treatment was associated with higher expression of IL‐33 (*p* = 0.031) and IL1RAP (*p* = 0.045) mRNA in sputum. There was a significant correlation between IL1RAP mRNA and sputum neutrophils (*p* = 1.13 × 10^−13^) and between IL‐1RL1 mRNA and sputum eosinophils (*p* = 2.2 × 10^−16^). In addition, there was a significant correlation between IL‐33 (*p* = 3.4 × 10^−5^) and IL1RAP (*p* = 2.2 × 10^−16^) mRNAs and blood neutrophils. Further details are provided in Appendix S1.

One‐way analysis of variance (ANOVA) testing reveals that blood neutrophils, blood lymphocytes and blood monocytes do not alter the distribution of blood IL1RAP gene expression across the granulocytic groups (*p* = 0.874962). However, when these are included as interaction terms with regard to TAC subtype, it attains significance (*p* = 0.0227). Adjustment of the potentially confounding effect of these cells would result in a more TAC2 distribution of IL1RAP mRNA, a finding which further supports our investigation's finding of IL1RAP being associated with neutrophilic asthma.

## 4 DISCUSSION

We obtained cell‐specific IL‐33‐up‐regulated gene signatures from various cells involved in asthma pathogenesis. Signatures were highly cell‐specific with little gene overlap despite sharing enriched pathways for TNF, NF‐κB and JAK/STAT signalling pathways and Th17, Th1 and Th2 cell signalling. We observed enrichment of IL‐33‐up‐regulated signatures predominantly in the sputum of asthmatics with neutrophilic or the TAC2 molecular phenotype of asthma. The mast cell IL‐33‐up‐regulated signature was also enriched in eosinophilic inflammatory phenotype as well as the neutrophilic and mixed phenotypes. Enrichment of IL‐33‐up‐regulated cell signatures was associated with enhanced ILRAP, but not that of IL‐33 or IL1RL1, mRNA expression in the same samples. The enrichment of IL‐33 signatures in neutrophilic (N) and mixed granulocytic (M) asthma sputum transcriptomics was partially replicated in the ADEPT cohort. Although similar trends were observed, significance was attained only with the IL‐33‐stimulated basophil‐derived signature.

Although there was minimal gene overlap between IL‐33‐stimulated basophils, mast cells, ILC2s and Tregs, common downstream signalling pathways were identified. This may reflect distinct cell‐type responses and timing‐specific issues following IL‐33 stimulation. This suggests that the expression of single genes or a group of genes may not adequately indicate common cell activation effects. Activation of the same downstream pathways with potentially the same cell activation effects may, therefore, provide a better description of IL‐33 cell‐specific activation than individual genes themselves.

IL‐33 mRNA expression was similar across all subjects, providing little insight into IL‐33‐driven pathology. IL‐33 is difficult to study as no reliable sensitive assays are available^40^ due to the transient expression of the active reduced form.^41^ This means that absolute gene expression and protein levels do not accurately reflect the status of active IL‐33. Hence, our approach in this study provides a useful alternative means of assessing this.

The IL‐33 receptor is a complex receptor constructed of IL1RL1 and IL1RAP. IL1RL1 and IL1RAP mRNA expression was not differentiated amongst asthma cohorts in blood but IL1RL1 was elevated in the sputum of severe eosinophilic asthma. IL1RAP mRNA expression was highest in severe neutrophilic and mixed granulocytic asthma. Together with the enrichment of the IL‐33‐stimulated cell signatures, these data provide evidence for IL‐33 pathway activation in severe neutrophilic asthmatics in addition to those with eosinophilic asthma. This supports evidence from recent clinical trials where anti‐IL‐33‐targeted therapies worked better in asthmatics with lower blood eosinophil levels.^15^, ^16^

Our findings of high enrichment of the IL‐33‐activated signatures in neutrophilic patients contrasts with the concept IL‐33 being a driver of Th2 biology and of blood eosinophils being biomarker of IL‐33 pathology.^10^ We identified the JAK/STAT pathway as being activated downstream of IL‐33 interaction with its heterodimer receptor. The JAK–STAT pathway can be activated in both T2 and non‐T2 pathologies^42^ which is further supported by our finding of IL1RAP mRNA being elevated in neutrophilic subjects. In a previous study, the expression of IL1RAP mRNA in sputum was significantly associated with sputum neutrophilia and airflow obstruction in asthma.^43^ Furthermore, the human protein atlas shows high levels of IL1RAP protein in neutrophils (https://www.proteinatlas.org/ENSG00000196083‐IL1RAP/tissue/granulocytes).

IL1RAP mRNA is increased in PBMCs from children with asthma compared with healthy controls, and its expression is enhanced with increasing asthma severity and during exacerbations.^44^ Selective blocking of IL1RAP with a monoclonal antibody (mAB‐mR3) inhibits signalling not only by IL‐33 but also IL‐1 and IL‐36 pathways.^45^ In an ovalbumin‐driven murine model of asthma, MAB‐mR3 reduced lung neutrophilia as well as suppressing eosinophilia. This is in line with the predominant neutrophilic inflammation induced by IL‐33 in the skin and peritoneal cavity and the ability of MAB‐mR3 to suppress neutrophils in a monosodium urate (MSU) crystal‐induced model of gout.^45^ The IL1RAP single nucleotide polymorphism (SNP) rs10513854 is associated with childhood wheeze whilst the rs9290936 SNP is associated with persistent wheeze.^46^ These data support a functional role for the raised levels of IL1RAP seen in this study in patients with TAC2 or neutrophilic asthma. Furthermore, it suggests that these patients may respond to anti‐IL1RAP‐directed therapies.^45^

IL1RL1/ST2 mRNA expression being significantly up‐regulated in the sputum of eosinophilic asthmatics is consistent with the report of a strong association of IL‐33 and its receptor with severe eosinophilic asthma.^10^ This may be a reflection of the high expression of IL1RL1/ST2 on eosinophils^10^ and mast cells^47^ but does not necessarily measure cellular activation by IL‐33.

Mixed clinical trial results have led to the termination of the anti‐ST2 biologic GSK3772847 (GlaxoSmithKline, NCT03207243, NCT03393806) programme although other clinical studies are progressing. Initial results for the anti‐IL‐33 biologic REGN3500 (Regeneron Pharmaceuticals) have shown it to be effective versus placebo (NCT03387852), but it was less effective than the anti‐IL‐4/IL‐13, dupilumab. This may reflect the recruitment of T2 asthmatics with high blood eosinophils and FeNO and this is supported by the recent clinical trial of the anti‐ST2 astegolimab (Genentech) which reduced the annual exacerbation rate in both eosinophilic but more so in non‐eosinophilic severe asthmatics.^15^

Although our study has revealed some novel downstream effects of IL‐33 in asthma, there are some factors that may interfere with the interpretation of the results. The enrichment of IL‐33 pathway activation in T2‐low asthma may be influenced by oral corticosteroid (OCS) use in severe asthmatics driving neutrophilia.^48^ However, OCS use was highest in patients within the TAC1 cohort compared to those in TAC2 and TAC3 groups.^14^ This suggests that neutrophilia and IL‐33‐up‐regulated signature enrichment are not simply an acute effect of OCS in this cross‐sectional study. In addition, T‐cell responses may vary depending on the presence of specific IL‐33 polymorphisms present.^10^ The role of these polymorphisms needs to be further investigated.

We did not see significant enrichment of IL‐33‐activated signatures in the tissue compartments studied (bronchial and nasal brushings and bronchial biopsies). This may reflect the preferential enrichment of immune cells in sputum compared with biopsy samples. Furthermore, it may be a result of the effect of medication in suppressing tissue inflammation as previously described.^39^ It was surprising to see enrichment of an endothelial cell signature in sputum but there is evidence for the presence of endothelial progenitor cells^49^ and endothelial cell microparticles^50^ in the sputum of COPD patients.

There are some limitations to this study including incomplete matching of tissue and sputum samples within U‐BIOPRED with less than 5 TAC2 subjects having had bronchial biopsies or brushings collected, that may account for the lack of enrichment of the IL‐33 signature in the airway samples compared to sputum rather than the use of activation signatures per se or the reflection of other cellular/molecular processes. In addition, the lower numbers of samples within the ADEPT sputum samples and the relative paucity of neutrophilic (*n* = 7) and mixed granulocytic (*n* = 5) subjects may account for only a partial validation of the U‐BIOPRED data. IL‐33 activation signatures derived from bulk RNA sequencing of cells may potentially reflect contamination with other cell types which will be resolved using single cell RNA‐sequencing‐derived signatures. Future studies involving severe asthma molecular phenotyping should strive to obtain samples from different compartments from all subjects to reduce the low overlap between compartments seen with many studies. The data presented here are a step towards the molecular classification of asthma. This type of approach has proved critical for indicating personalized targeted therapies in other chronic inflammatory and immune‐based diseases.^51^

The low expression of IL‐33 mRNA in all sample types, aside from bronchial biopsies and brushings, the lack of differentiation of IL‐33 gene expression between patient groups and the lack of corroboration with the enrichment of the IL‐33 signatures indicates that IL‐33 gene expression alone is insufficient to demonstrate IL‐33 activation. Indeed, it is evident that IL‐33 is not predominantly regulated at the level of mRNA expression, which is preformed and stored intracellularly within the nucleus where it may have additional roles.^20^, ^21^ Without a specific assay of IL‐33 protein expression and activity, it is important to make use of IL‐33 cell activation signatures to indicate IL‐33 presence. This approach could be used in other studies where cellular activation status is more important than cell counts alone and where alarmin detection is also problematic as with thymic stromal lymphopoietin (TSLP). Analysis of TSLP‐stimulated cell signatures in asthma should be undertaken in future studies. In addition, RNA‐seq data from airway tissues, sputum or BAL cells should be obtained before and after successful, or even unsuccessful, treatment with drugs such as tezepilumab^52^, ^53^ to define a responder signature that could be used to help clarify potential responder populations.

In summary, we have shown an enrichment of IL‐33‐stimulated cell signatures in the sputum of severe neutrophilic asthmatics, extending the current genetic association studies and concepts of IL‐33 playing a preferential role in severe eosinophilic asthma. This analysis highlights the need to examine the effect of IL‐33‐targeted treatments in non‐T2 subjects.

## AUTHOR CONTRIBUTIONS

John H Riley, Stewart Bates and IMA contributed substantially to conception or design; Yusef Eamon Badi and Adam Taylor performed the analysis; Yusef Eamon Badi, Adam Taylor and Nazanin Zounemat Kermani contributed to data acquisition, Sven‐Eric Dahlen, Barbora Salcman, Silvia Bulfone‐Paus, AKSJ, MN, DJC and Batika Rana contributed data or analysis tools; Nazanin Zounemat Kermani, Sally Worsley, Sharon Mumby, Sven‐Eric Dahlen, MN, Karen Affleck and Kian Fan Chung contributed substantially to analysis or interpretation; Sven‐Eric Dahlen, MN, Kian Fan Chung, Karen Affleck and Sharon Mumby contributed substantially to intellectual content development or critical review and Yusef Eamon Badi and Ian M. Adcock contributed substantially to writing the paper. All authors approved the final version of the manuscript.

## CONFLICT OF INTEREST

Adam Taylor, John H Riley, Sally Worsley, Karen Affleck and Stewart Bates have worked or are currently working for GSK. Batika Rana is currently working for Orchard Therapeutics and Yusef Eamon Badi is currently working at BenevolentAI. MN, Silvia Bulfone‐Paus, Kian Fan Chung, DJC and IMA have received investigator‐led research grants from GSK. YB and Barbora Salcman are supported by BBSRC CASE awards. DJC has received investigator‐led research grants from AstraZeneca and Genentech. There are no other conflicts of interest regarding this manuscript.

## Supporting information


Appendix S1

## Data Availability

The data that support the findings of this study are openly available in Gene Expression Omnibus (GEO) reference numbers GSE76262 and GSE69683.
