# Supplementary material for: IL1RAP expression and the enrichment of IL‐33 activation signatures in severe neutrophilic asthma
Source: Allergy. 2022 Aug 28;78(1):156–67. doi: 10.1111/all.15487 (PMC10086999; doi:10.1111/all.15487)
Supplement: Supplementary file 1 — Appendix S1 [file ALL-78-156-s001.docx]

**Supplementary data**

**Supplementary Methods:** in vitro IL-33 stimulation of mast cells (based on reference 1).

Peripheral blood mast cells were extracted using Ficoll-Paque method at 1.077 g/ml and centrifugation at 400 x g. These were then magnetically separated and diluted to 1 x 106 cells/ ml. Human primary mast cells were then cultured and maintained at a concentration of 0.5-1x106 cells/cm2. Culturing was performed in 500 mL of StemSpan™SFEM supplemented with 100 U/mL penicillin and 100 μg/mL streptomycin, 100 ng/mL human SCF, 50 ng/mL human IL-6, 10 ng/mL human IL-3 and 10 μg/mL LDL for 4 weeks. From week 5 onward, culturing was performed in 500 mL of IMDM supplemented with 100 U/mL penicillin and 100 μg/mL streptomycin, 100 ng/mL human SCF, 50 ng/mL human IL-6, 50 mMol/L 2-mercaptoethanol, 0.5% BSA until maturity was reached at week 8. Mast cells were then centrifuged at 300 x g for 5 min and resuspended cell at a concentration of 0.5-1x106 in 500 mL of IMDM supplemented with 100 U/mL penicillin and 100 μg/mL streptomycin, 100 ng/mL human SCF, 50 mMol/L 2-mercaptoethanol, 0.5% BSA in addition to either IL-33 (at a concentration of 50 ng/mL), or nothing (for control samples), for 24 hours at 37°C with 5% CO2.

**Supplementary Results**

The expression of IL-33, IL-1RL1 and IL1RAP in the blood and sputum was related to cell counts and treatment. There was a significant increase in IL1RL1 (p=9.0x10^-7^) and IL1RAP (p=0.033) mRNAs in sputum in patients prescribed OCS therapy compared to those not on OCS (**Supplementary Figure S7 Upper panels**). In contrast, LABA treatment was associated with higher expression of IL-33 (p=0.031) and IL1RAP (p=0.045) mRNA in sputum (**Supplementary Figure S7 lower panels)**. We also found a small but higher expression in IL-33 (p=0.027) and IL1RAP (p=0.0003) mRNA in blood of patients on OCS treatment (**Supplementary Figure S8 upper panels**). LABA treatment was not associated with any differences in blood mRNA expression of any of these genes (**Supplementary Figure S8 lower panels)**.

We observed a significant correlation between sputum IL1RAP mRNA and sputum neutrophils (p=1.13x10^-13^) and between sputum IL-1RL1 mRNA and sputum eosinophils (p=2.2.x10^-16^) (**Supplementary Figure S9**). In addition, there was a significant correlation between blood IL-33 (p=3.4x10^-5^) and IL1RAP (p=2.2x10^-16^) mRNAs and blood neutrophils (**Supplementary Figure S10**). There was a significant positive correlation between peripheral blood IL1RL1 mRNA (p=7.8x10^-7^) and a negative correlation between IL1RAP mRNA (p=0.012) and blood eosinophil counts (**Supplementary Figure S10 and Supplementary Table S15**). There was no correlation between blood mRNAs and sputum neutrophil counts but a significant positive correlation between blood IL1RL1 mRNA expression and sputum eosinophil counts (p=0.005) (**Supplementary Figure S11**).

One-way analysis of variance (ANOVA) testing reveals that when blood neutrophils, blood lymphocytes and blood monocytes are included as interaction terms for the statistical association of blood IL1RAP gene expression and granulocytic subtype, these do not alter the distribution of blood IL1RAP gene expression across the granulocytic groups (p=0.87). However, when these are included as interaction terms with regard to transcriptome associated cluster (TAC) subtype, it attains significance (p=0.023). When plotted by TAC, blood neutrophil and monocyte counts are elevated in TAC1 and lymphocytes are elevated in TAC1 and TAC3. Adjustment of the potentially confounding effect of these cells would result in a greater TAC2 distribution of IL1RAP mRNA, a finding which further supports our finding of IL1RAP being associated with neutrophilic asthma.

**Supplementary Table S1** Cell specific genes tables; (A) cell specific genes generated from single cell data and (B) neutrophil cell specific signature.

**A**

| **Cell type** | **Gene Symbol** | **Reference** |
| --- | --- | --- |
| **Activated endothelium** | SELE, SELP, ACKR1, ZNF385D, TSPAN7, MEOX1, NRN1, VCAM1, VWF, ABCG2, ANGPT2 | (2) |
| **Mast cells** | TPSB2, CPA3, HPGDS, MS4A2, SLC18A2 | (2) |

**B**

| **Abbas Neutrophil cell signature** | ABTB1, AMPD2, C5orf6, CCR3, CDA, CKLFSF2, CLC, CREB5, CTBS, DcR1, EST, FCGR2B, FCGR3B, FLJ10298, FPRL1, FRAT2, GPR27, GPR43, HSPA6, IL8RA, IL8RB, KIAA0779, KIAA1126, KRT23, LENG4, LENG5, MAD, MGC10500, MGC14126, MGC16353, MPPE1, MSCP, NCF4, NRBF-2, PHC2, PROK2, RALB, RNF141, SEC14L1, SEPX1, STX3A, TM4-B, VMP1, VNN2, XPO6 | (3) |
| --- | --- | --- |

**C**

| **Sidhar Eosinophil cell signature 1** | PRSS33, GPR44, CLC, ADORA3, IDO1, OLIG1, EMR1, CCR3, VSTM1, LGALS12, CAT, CAMK1, CD9, SIGLEC10, TKTL1, FLVCR1, P2RY14, LOC283070, FAM101B | (4) |
| --- | --- | --- |
| **Sidhar Eosinophil cell signature 2** | PRSS33, SIGLEC10, GPR82, PDE4D, PIK3R6, CAT, SORD, SLC29A1, MARK3, CASP3, EPN2, FGFR2, GADD45A, AREG, AREGB, CLC, PTGDR2, CYP4F12, IL1RL1, SIGLEC8, GFI1B, PAPSS1, MAX, ARHGEF6, RIPK2, CSF1, CACNA1D, GATA1, CCL23, IL5RA, RPL13P5, GSTM4, OLIG2, CEBPE, DAPK2, CYSLTR2, ARL6IP6, LINC00085, PYROXD2, EXOC3, SEMA7A, VSTM1, SLC16A14, EPN2-IT1 | (4) |
| **Sidhar Eosinophil cell signature 3** | CACNG6, CCL23, GPR44, HSD3B7, IDO1, SIGLEC8 | (4) |

**Supplementary Table S2** IL-33 gene signatures with cell specific genes indicated in red

| **Signature name** | **Details of Stimulation** | **Genes** | **Reference** |
| --- | --- | --- | --- |
| Mast cell IL-33 signature of up-regulated genes | Stimulated with IL-33 at a concentration of 50 ng/mL for 24 hours at 37°C with 5% CO_2_ | MUC4, EBI3, AC010247.1, CCR7, BX255923.2, XIRP1, IL5, IL13, CDH13, ITGA1, ROR1-AS1, AC099552.1, GCGR, MMP7, LIMS2, SLCO5A1, ABTB2, COL16A1, AC083837.1, SSTR2, CRTAC1, LINC02690, C3, POU2F2, CCL1, FEZ1, RIPOR3, HLF, COL5A3, AC026310.1, CXCL8, CHGA, CD70, MMP9, C1QTNF1, LINC01215, CCL5, TCERG1L, TNFRSF1B, TSPAN18, RASGRP1, FCER2, GPR68, FCRLA, MIR3142HG, LINC02605, MGP, MT1L, TPSG1, LRFN5, BCAR3, PIGR, ZBTB7C, TRPM6, SLAMF7, COL15A1, KCNK13, TNFRSF9, SHC4, C15orf48, TNFRSF18, PDE4C, CFAP46, NAV3, DSE, AC110611.2, KLF5, MRAP2, IGLV2-18, AC034199.1, NEDD4L, MAOA, MMP2-AS1, MIR155HG, SERPIND1, WFS1, IGLV3-17, EDIL3, IL7R, TLR7, MMP19, OLR1, NMNAT3, BIRC3, CD200, GCKR, RGS9, MT2P1, TRIM9, FAM186B, CACNA1B, PXYLP1, GJA4, VTN, TTC39A, PLA2G4C, IGLV3-21, BAMBI, MT2A, AL160408.3, WLS, HIVEP2, ACE, TNFRSF8, ASB2, AGMAT, HAPLN3, TTC39C, AC114977.1, SYNGR3, IL15RA, MARCKSL1, VEGFC, CD36, MAP7D2, KCNK5, COLGALT2, CSF2, TGFBR3, TLL2, MIAT, RGS3, PIEZO2, PCSK5, TNFAIP3, TMEM176A, SCN9A, CCL24, NUAK1, ZNF804A, TMEM176B, AKR1C3, GPR50, MT1G, ADGRA3, SGPP2, RCN3, CDHR1, ACHE, SORCS2, KISS1R, P2RX5-TAX1BP3, STAT4, AGRN, THBS1, COL6A5, TNF, SLCO4A1, SLC22A31, HLA-DRA, CARD11, B4GALNT1, TGM2, ENPP1, NFKBIZ, SHISA2, IGFBP4, IRF4, LRG1, KCNH2, TLR2, CXCR5, INA, SEPTIN4, DNPH1, CYP1A1, NFKB2, CTSH, IL1B, MAMLD1, PTGIR, GDF11, TRAF1, MT1DP, CD40, FHL1, IL18RAP, CCDC102A, RASGRP3, LTA, LINC01285, CTXN1, LRIG1, CHST1, DLEU1, MGAT5B, PTGES, DAB2IP, KIRREL3, GP1BA, SERPINF1, MUC20, IL32, ZFPM2, H2BC11, TMEM217, NETO2, UBE2E2, GLIS2, FN1, PPP1R3C, PHGDH, GCH1, CPNE3, ASIC1, TNFSF4, MAP3K8, COL7A1, RRAD, IL2RG, EPOP, IRAK2, ZC3H12A, P4HA2, LINC00892, AC124798.1, TINCR, RYR1, SLC7A11, WNT9A, VWCE, PLA2G4A, IL4I1, GPC6, RGL1, PYCR1, H2AC6, NEK10, ZCCHC12, SLC16A1, MTHFD1L, NMB, IL2RA, NTN1, NT5DC2, RAI2, FLVCR2, ZFPM1, MFSD12, CD74, AC007278.1, AL645608.8, COL13A1, TMEM120A, SYT12, N4BP3, TNFAIP2, AL133520.1, SPAG1, ROBO1, PDLIM4, CYP4F22, DIXDC1, AQP3, ADAM8, PRSS23, MYL9, TNFSF9, PTGES3L, IL10, AL031283.1, H2BC5, QPCT, MYO1E, CYP27B1, AC093503.2, MARCKS, GAS6, PTPN13, IGLV3-19, PDIA5, ARNT2, LINC00475, ANK2, ROR1, GSTP1, GBP4, KIF26B, IL17RB, PTGS2, FERMT1, ITGB4, HLA-DPB1, SYPL2, SLC16A9, IL34, PCSK9, TNFSF14, LTB, COL9A2, LHFPL6, SEMA4B, AC007278.2, EGR2, CACNA1H, TJP1, BATF, LAMP3, CCL4, AC245100.7, CBS, CTTN, SEC11C, LINC02694, NOS3, TNIP1, PIM2, MREG, GADL1, RELB, TP53RK, MFSD2A, IL1A, SFRP2, ST6GAL1, CHPF, AC120498.6, LINC01134, H2BC12, MEX3A, SPTA1, PRG2, CNR2, PEAK1, TNS3, SPARC, KIAA1958, BCL9, MMP11, GOLGA2P10, FRMD4A, STARD10, AC107959.3, PSD, DLEU2, WT1, SDF2L1, MMP2, PRCP, LOXL4, CCDC6, PIFO, PGAP4, TYMSOS, ATOX1, SERPINE1, S100A9, NME1, GPRC5C, MT1E, NACAD, MRC1, TMEM198, NR4A3, AC078785.1, OCIAD2, SAMD15, CA12, CNTNAP1, AHRR, TENT5A, MYDGF, SCG2, SMAD3, NFKBIA, TIGIT, REEP2, IGFL2, NAMPTP1, LST1, INHBA, RASL11A, CYP7B1, LINC02068, CCL3, IL27RA, FBXL19-AS1, EFCAB12, TMEM64, ALOX15, CHST7, PSAT1, VWA8, SLC27A2, H2BC4, OAS3, ARHGAP23, RDH10, CD81, AL445524.1, AL137060.3, EDARADD, IL2RB, FKBP5, APOC1, AC007384.1, SORD2P, AC011611.3, PKMP1, BAALC, HLA-B, PKM, IL19, CLCN4, OLFM2, CGN, C1orf53, SBK1, DPP4, TMEM25, P4HB, SPATC1, ALDH8A1, FIRRE, H1-2, DYRK3, CYTIP, AC034102.1, VCAM1, IL1RL1 | - |
| Mast cell IL-33 signature of down-regulated genes | Stimulated with IL-33 at a concentration of 50 ng/mL for 24 hours | AC104248.1, KCNK3, MRGPRX6P, VWC2, AL356489.1, FAR2P1, ERVFRD-1, JPH4, KCNJ5, ZBTB16, PVALB, AL670729.3, KY, PADI2, SCN2A, UNC80, ANXA8L1, TGM5, MYLK, MRGPRX7P, ADRA2A, AL136018.1, MRGPRX2, CDSN, IFI30, RGS4, REM1, DNAH8, C1orf127, RPS17P1, ABLIM3, AC091563.1, FREM1, P2RY10, AL356489.3, OR10Y1P, SGSM1, TRBV26OR9-2, UNC79, KRT72, MAS1L, GPR162, SYN3, LINC02395, SLFN14, AL356489.2, PLXNA4, AP001122.1, C11orf45, MATK, LINC02008, RASL10A, NTM, PTENP1-AS, HNF4A-AS1, SPINK4, COL25A1, AC073869.3, OTOA, DEPTOR, Z85996.3, OR8A1, ANXA8, RASGEF1C, FAM20A, SIGLEC15, GATA3, SCN7A, LIPN, KLF2P4, EMP1, CD226, AL139142.1, VWA5B2, M1AP, SLC38A11, PDE7B, ZNF467, CYP4F30P, FAR2P4, HGD, SORBS3, CALB1, RASGEF1B, IQGAP3, PANX3, S1PR1, CALCRL, PRR5L, RAB3B, LINC02147, ICAM4, AL049552.2, UTS2, AC018865.2, LINC02773, CPED1, KRT73, CHST8, DNM1P35, ITGA3, SLC22A18AS, AC006230.1, PTPRN2-AS1, PICART1, CPNE5, AC019197.1, PPP2R2B, LILRB3, CD2, TP53I11, ADGRE4P, OR10V3P, PDK4, AC020907.5, KRT17P8, PDZPH1P, DHRS9, PLXDC1, ATP1B1, CACNG8, CLDN9, AC004233.2, EEF1A1P14, SNCG, AL136456.1, AC116424.1, ASPM, REXO5, TMCC3, MAS1LP1, F8, CREG2, ADAM11, CCNA1, RBM20, C12orf42, TNNI2, SNX21, TNS1, AC087369.2, FMN1, RTN4R, MPIG6B, SFTPD, BIRC7, LINC00607, SLC37A2, GJA3, CDH23, ESR2, AL133313.1, BTG1P1, LINC01918, S100Z, ANK3, HBD, SLC22A10, OR8G5, NEURL1B, LILRB2, TXNIP, RGS5, MS4A3, ADGRG7, AP003119.1, MAF, TOX2, AL021917.1, TSPYL2, ARAP3, TSPAN11, NPTX1, LINC02082, CREB3L1, SCN1B, SSPOP, CSF3R, CHST15, NFE2, GPR34, TIMP2, SLC38A6, LINC00323, HK3, LTB4R, AL021392.1, SGIP1, EFHC2, LRRC7, IRAG1, APBB1IP, SHC3, BX571818.1, CR1, ZNF367, PM20D1, ITPR3, ADGRG4, TCTEX1D1, SEPTIN7P9, PITPNM3, NAALADL2, CTSG, SIGLEC12, ST14, HPN, AC009652.1, SLC15A2, UBE2D3-AS1, KIF18B, SMAD6, ADAMTSL3, AC113414.1, MLPH, OR8G1, SNX29, CYRIA, CFAP58-DT, MAGEB2, ZMAT4, MYO16, NABP1, ZMYND15, CA8, SLC27A3, LINC00987, SERPINB1, MKI67, CRYBB1, SEMA3D, AC011899.2, AIF1, PDE2A, RHBDF1, MOB3B, SEMA6B, FAM111B, LINC01529, FAM216B, MS4A6A, ARHGEF37, SGK1, PTCH2, TBC1D2, RALGPS2, LURAP1L, AK5, GCSAM, ADRA1D, ZBTB20, CNIH3, AL355365.1, KIAA0040, B3GNT5, AL078590.2, AC020914.3, STAB1, AC009145.1, IL16, E2F8, ICAM5, LTB4R2, NRGN, PIK3IP1, GFOD1, RYR3, AC108053.1, LINC01366, PPIAP41, FBP1, FCER1A, FOXN2, SERPINB9P1, MIR223HG, RNASE6, CXCL16, LBH, OR8G3P, AC112178.1, TREML2, SHANK1, KIAA1614, NLRC4, AC010210.1, PLEKHA5, LINC02829, TTN, BTBD19, EVPL, AC018450.1, SYNE2, MIR3681HG, AC060766.6, ARL15, NHSL1, AC092650.1, IL12A-AS1, SIPA1L2, SVOPL, CDK1, RGS13, SCIN, SCPEP1, DLGAP5, CCDC188, COBLL1, UBE2C, LINC02656, ANLN, AC104964.2, RHOQP3, TNXB, AL590617.2, EGFEM1P, PEPD, PRKG2, SLA, SESN3, ELFN1, DPY19L2P1, PLXNA2, ARHGEF40, HS3ST1, AC080013.3, CSF2RA, ABHD4, LINC02115, CIDEB, C3AR1, LOXL3, LINCR-0001, INKA1, ZNF483, FOXF1, NDRG2, CDK15, ARRDC4, TNFRSF14-AS1, CCDC30, IL18BP, AL356804.1, EGR3, SDK2, SLC22A18, TXLNB, KREMEN1, LINC01583, HACD4, AC010175.1, MROH7, AURKB, F2R, ANGPT2, SIGLEC8, CCL23, CYP4F12, PTGDR2, FGFR2, MS4A2 | - |
| Basophil IL-33 signature of up-regulated genes | Stimulated with IL-33 at a concentration of 20 ng/ml for 24 hours | AQP9, GNLY, CRLF2, THBS1, OLR1, HIST1H2BD, SPOCK1, INHA, ATP10D, TMEM140, BMP6, LOC653560, ABCA1, TGIF1, NFKB2, STAT4, LOC642567, SOD2, LOC389791, C3AR1, EVI2A, GBP2, IPO11, MEGF9, VNN3, LOC730092, FEZ1, RELB, HIST2H4A, LIPN, FMNL2, TGFBR3, PTGDR, CALCRL, MGC42367, HIST1H2AC, PTCHD1, ECHDC3, ABCG1, TFDP1, ZNF200, NINJ1, LOC132241, TTYH2, VNN1, FLJ42986, D2HGDH, BACH2, PHTF1, C5orf53, NCRNA00085, GABRB1, HIST2H4B, RBKS, LOC339192, MGC61598, RBBP8, NFKB1, IL4R, BRE, DUSP16, HIST1H4H, LOC728431, PRDM8, HAS1, CXCR4, FTHL3, PILRA, IFIT2, TNFAIP6, IL18R1, PIM3, ARRDC2, PEX11G, EXD3, MITD1, C19orf28, ATP2B1, IFIH1, MGC16121, CYP21A2, MAST3, LOC648164, ST6GALNAC2, SH3TC1, AHI1, RNU4-2, PTPRC, LYPD3, NAB1, PLAUR, LQK1, INDO, LOH12CR1, LOC388969, PERP, SLC39A8, CDKN2AIP, FNDC3A, FLJ35776, DUOX2, SLC23A2, PCSK5, LOC338799, CMIP, MEP1A, C8orf44, LOC730256, FTH1, Mar-03, MGC11082, LOC285741, ZNF521, CBLB, LOC729009, CXorf65, KCNJ8, RCN1, C2orf55, SDHAF1, TNFAIP8L2, KLHL21, MRPL1, SLC36A4, GDPD3, C7orf53, VEGFA, LYSMD2, FPR1, KCNE1L, PRG1, HDAC4, SIPA1L1, PLA2G7, ZNF613, PPAPDC2, FLJ39653, PLGLB1, LOC727820, HIST1H2BG, TTC9C, ZNF585A, SLA, QPCTL, AGPAT6, LOC441376, LOC653853, SNORA7B, HIVEP1, FAIM3, GAB1, SLA2, GHRL, MYOM1, LRRC6, LOC644879, TNFAIP8L1, PDGFC, SLC25A15, CHST2, LIMK2, SLC26A11, NSD1, COL6A1, FTHL11, PKIA, NFE2L3, CDKN2C, LOC100133930, MOCOS, LOC646043, PLD6, NF1, ENG, FAM43A, FTHL8, MAGED4B, BAMBI, PLCB1, RASL11A, HIST1H3D, FLJ11783, NUAK2, EID2, B3GAT1, TFPI, SMOX, LOC100130828, PDE8A, MUC20, FLJ77644, PLGLA, APOLD1, AMDHD1, GPR160, HIST1H3F, KBTBD7, ANGPT2, VNN2, LGALS12, IDO1, PDE4D, GFI1B | (5)  Accession GSE64639 |
| Basophil IL-33 signature of down-regulated genes | Stimulated with IL-33 at a concentration of 20 ng/ml for 24 hours | KIAA0564, CCR2, TCN1, CEACAM1, FOSB, MMD, HK3, CISH, LOC399940, CCR1, SPRYD5, ITGAM, MS4A3, DUSP5, SOCS2, LOC100129034, PIM1, SFXN1, CKAP4, SOCS1, ST6GAL1, IFITM1, FAM65B, LOC732393, LTB, LST1, NDFIP2, FLNB, LAT2, LAT, SELL, FLJ43093, CD69, H3F3B, PTGER3, KRT86, BHLHB2, STK17B, SLC45A3, CCL3, ALOX5, ROPN1L, KRT81, JUN, CORO6, C10orf128, OSM, HBEGF, E2F2, GZMB, OSBPL3, XK, C9orf140, KLF2, TIMM8A, RGPD5, KCNK17, FAM113B, TCP11L2, CCL23, MS4A2, CAMK1 | (5)  Accession GSE64639 |
| ILC2 IL-33 signature of up-regulated genes | Stimulated with IL-33 at a concentration of 50 ng/ml for 7 days | GZMA, IL8, IL5, MYB, PTGS2, CTLA4, GZMB, BHLHE22, IL13, MT1A, CCL3L3, CSF2, TCTN3, C10orf61, GBP5, MT1E, LOC387882, SLC27A2, GBP2, MTE, RGS2, TNFRSF8, ITGA1, LRIG1, GPR55, PRDM1, FCER1G, LIMA1, BASP1, CCR1, CD74, IL26, HSH2D, SYT11, CD38, MYL6B, PHLDA1, IRF4, IL2RA, GBP4, MIR21, PIM2, PLA2G4A, GBP1, ELL2, IL17RB, CCL3, MAP3K8, ATP8B4, EPSTI1, ATP9A, MT1F, RHOBTB3, NPTX1, MX1, INPP1, RASGRP2, ENTPD1, MUC1, TUBB6, PSEN2, GNA15, CCL3L1, UQCRC2, PSMD1, WARS, PTGER3, ICOS, TRIB1, SH2D1A, MLLT11, H1F0, CXorf21, NFKBIZ, TAP1, BAG2, TCF4, OSTF1, TMEM49, GPR56, CHCHD3, TESC, PELO, TSPAN13, RAB9A, SRGN, ZEB2, PSMB8, ERAP2, ASCL2, MAPK6, NFIL3, ALOX5AP, IL4, CREG1, TMEM64, TNFSF10, BATF, QPRT, DCBLD2, RNASEN, UBE2Z, ZBTB32, CSF2RB, ERI1, PAGE5, RNF19B, VNN2, IL1RL1, P2RY14, NCF4, SLC29A1 | Accession GSE72433 |
| ILC2 IL-33 signature of down-regulated genes | Stimulated with IL-33 at a concentration of 50 ng/ml for 7 days | FAM102A, RXRA, MATK, ASTN2, IL7R, ARRDC3, LIME1, ABLIM1, TCEAL3, NR1D2, ZBTB16, KRT1, SPOCK2, PLCG1, SCN2A, NEK6, APP, SLC5A3, AMICA1, TP53I11, OSBPL7, ATP1B1, LSP1, TNFRSF1A, CAST, SERPINE2, FAM134B, SNORA5A, KIT, SH3GLB2, LEF1, MAZ, GPR44 | Accession GSE72433 |
| HUVEC IL-33 signature of up-regulated genes | Stimulated with IL-33 at a concentration of 50 ng/ml for 4 hours | PDZD2, CCL20, CSF2, CXCL2, CX3CL1, CXCL1, IL6, ICAM1, CXCL6, TNFAIP2, IL8, SERPINA3, UBD, CCL2, BIRC3, NLF2, TNFAIP3, CEBPD, SOD2, PTGS2, SLC7A2, TNFAIP6, CHIC2, NFKBIZ, RND1, LOC387763, CXCL10, EBI3, IL18R1, NCOA7, F3, CSF3, CFB, CCL8, S100A3, IRAK2, IL1A, SDC4, C6orf128, RELB, HAS3, CCL7, APOL3, ATF3, ICOSLG, NFKB2, TNIP3, TNIP1, CXCR7, ISG20, SERPINB2, ZC3H12A, NFKB1, GCH1, MAP3K8, NFKBIA, IL7R, CNKSR3, UGCG, RASD1, FSTL3, ARID5B, POU2F2, DARC, TAP1, SLC2A6, CCRN4L, TNC, CBR3, LYPD6, CITED4, CXCL5, GFPT2, DNAJB9, GBP1, PDE5A, DSCR1, MMP10, PPAP2B, CTHRC1, PDLIM4, BMP2, ETS1, SQSTM1, IL32, PRIC285, TUBB2B, TRIB1, IL1B, GBP4, IL15, SAMD4A, C8orf4, TNFRSF9, FAM129A, SAT1, JAG1, PARP14, NINJ1, LTB, IFIH1, ABCG1, C1orf24, ELL2, PRRX1, HEY1, NKD2, TNFAIP1, TRAF1, F2RL1, SNFT, THRAP4, DDX58, CD83, IFNGR2, CD47, TNFRSF11B, PLAU, TGFB3, RGS2, PBEF1, CLIP2, FAM101A, SAMD9L, ADAMTS9, PAPLN, LOC730417, MSC, LAMC2, STAT5A, OPTN, DRAM, IFI30, MSX1, A4GALT, ESM1, NKX3-1, SMAD3, TIFA, PTPRK, ST5, SLC25A37, MAFF, IER3, ST6GAL1, NNMT, SERPINB8, LOC654103, APOL2, TNFSF15, SHB, KIAA1199, SBNO2, CXCL11, WTAP, SLC31A2, NFKBIE, HLA-B, PSTPIP2, OTUD4, SLC41A2, KYNU, ADAMTS4, RSPO3, MARCH3, STX11, IRF1, TNFSF10, IL4I1, CD69, HERPUD1, SSH1, ADAMTS1, IL15RA, ADORA2A, NRIP1, BIRC2, RHBDF2, MT1G, EFNA1, ZFPM2, MX1, HINT3, SAV1, LOC653778, CDK6, C3orf52, GPRC5A, OSGIN2, SLC11A2, CAB39, GALNT4, TRIM21, WWC1, TGFB2, C15orf48, B4GALT1, GBP2, SLC25A28, MOBKL2C, IFIT3, FBXO32, CSF2RB, LITAF, SOX17, MCTP1, SLC30A7, EDG1, CD200, KIAA0247, YPEL2, IKBKE, BCL6, OLR1, ZNFX1, SLC15A3, PANX1, SLC7A11, LOC646517, HRH1, BTG3, UPP1, DSE, CDC42EP2, AXUD1, PLA2G4C, FLJ31951, GPR37L1, TMPIT, GRAMD3, EHD1, ITGAV, COL27A1, OBFC2A, LRIG3, DUSP16, TICAM1, C6orf58, SELE, VCAM1, RIPK2 | (6)  Accession GSE37624 |
| HUVEC IL-33 signature of down-regulated genes | Stimulated with IL-33 at a concentration of 50 ng/ml for 4 hours | GJA4, SOX18, PRICKLE1, C3orf54, DUSP4, RUNX1T1, GFOD1, KCTD12, LYL1, GALNAC4S-6ST, TXNIP, ZNF395, CLEC14A, C8orf55, CYP1A1, KLHL3, TSC22D3, BMP4, KLF2, FAM124B, RARA, LFNG, PPP1R3C, LRRC33, TMEM37, SMAD6, LOC400451, CABLES1, PPFIBP2, JAG2, FRMD3, SDPR, DACH1, MAFB, RGC32, NUAK1, STX3, EFCAB4A, CMTM8, FAM84B, SLC45A3, KRT80, SORBS2, PCDH7, AXIIR, DKFZp761P0423, TMEM46, SERTAD4, GPR126, SALL2, CARD10, LMCD1, VPS37D, TRAM2, NDRG4, CDCA7, GIMAP7, HES1, GPR162, E2F2, HOXA10 | (6)  Accession GSE37624 |

Gene highlighted in red represent cell-specific genes that have been removed from the final signature.

**Supplementary Table S3** Pathway enrichment of the up-regulated IL-33 stimulated mast cell signature

| Term | Overlap | P-value | Adjusted P-value | Genes |
| --- | --- | --- | --- | --- |
| Cytokine-cytokine receptor interaction | 40/295 | 3.87E-21 | 9.22E-19 | CD40, CSF2, CXCL8, EBI3, CXCR5, IL2RG, TNF, IL27RA, IL18RAP, CCL5, CCL4, CCL3, TNFRSF8, CCR7, CCL1, IL10, IL32, IL15RA, CCL24, GDF11, TNFSF14, CD70, IL34, TNFRSF9, TNFRSF18, IL13, IL19, INHBA, TNFRSF1B, IL17RB, IL1A, IL5, TNFSF4, IL1B, IL2RA, IL2RB, LTA, TNFSF9, LTB, IL7R |
| Viral protein interaction with cytokine and cytokine receptor | 19/100 | 2.26E-13 | 2.69E-11 | IL10, CCL24, CXCL8, TNFSF14, IL34, CXCR5, IL19, TNFRSF1B, IL2RG, TNF, IL18RAP, CCL5, IL2RA, CCL4, IL2RB, LTA, CCL3, CCR7, CCL1 |
| NF-kappa B signaling pathway | 17/104 | 5.22E-11 | 4.14E-09 | EDARADD, CD40, CXCL8, TNFSF14, TNFAIP3, TRAF1, PTGS2, TNF, NFKB2, RELB, NFKBIA, IL1B, CCL4, LTA, LTB, CARD11, BIRC3 |
| Inflammatory bowel disease | 13/65 | 7.39E-10 | 4.40E-08 | IL10, SMAD3, IL13, IL2RG, TNF, IL1A, IL18RAP, IL5, IL1B, STAT4, HLA-DPB1, HLA-DRA, TLR2 |
| TNF signaling pathway | 15/112 | 1.22E-08 | 5.82E-07 | CSF2, DAB2IP, TNFAIP3, VEGFC, TRAF1, TNFRSF1B, PTGS2, TNF, MMP9, NFKBIA, IL1B, CCL5, LTA, MAP3K8, BIRC3 |
| Hematopoietic cell lineage | 13/99 | 1.46E-07 | 5.81E-06 | CSF2, ITGA1, GP1BA, TNF, FCER2, IL1A, IL5, IL1B, IL2RA, HLA-DPB1, HLA-DRA, CD36, IL7R |
| Asthma | 8/31 | 1.76E-07 | 5.99E-06 | IL10, CD40, IL5, IL13, PRG2, HLA-DPB1, HLA-DRA, TNF |
| Leishmaniasis | 11/77 | 5.64E-07 | 1.60E-05 | IL10, C3, NFKBIA, IL1A, MARCKSL1, IL1B, HLA-DPB1, HLA-DRA, PTGS2, TNF, TLR2 |
| IL-17 signaling pathway | 12/94 | 6.04E-07 | 1.60E-05 | NFKBIA, CSF2, CXCL8, IL5, IL1B, IL13, TNFAIP3, PTGS2, TNF, MMP9, S100A9, IL17RB |
| Malaria | 9/50 | 8.19E-07 | 1.95E-05 | IL10, CD40, CXCL8, CD81, IL1B, CD36, TNF, THBS1, TLR2 |
| Rheumatoid arthritis | 11/93 | 3.82E-06 | 7.66E-05 | IL1A, CSF2, CXCL8, IL1B, CCL5, CCL3, HLA-DPB1, HLA-DRA, LTB, TNF, TLR2 |
| Human T-cell leukemia virus 1 infection | 17/219 | 3.86E-06 | 7.66E-05 | IL15RA, EGR2, CD40, CSF2, SMAD3, MMP7, HLA-B, IL2RG, TNF, RELB, NFKB2, NFKBIA, IL2RA, IL2RB, HLA-DPB1, LTA, HLA-DRA |
| Chagas disease | 11/102 | 9.43E-06 | 1.73E-04 | C3, NFKBIA, IL10, ACE, CXCL8, IL1B, CCL5, SERPINE1, CCL3, TNF, TLR2 |
| Toll-like receptor signaling pathway | 11/104 | 1.14E-05 | 1.90E-04 | NFKBIA, CD40, CXCL8, IL1B, CCL5, CCL4, CCL3, MAP3K8, TLR7, TNF, TLR2 |
| Allograft rejection | 7/38 | 1.19E-05 | 1.90E-04 | IL10, CD40, IL5, HLA-B, HLA-DPB1, HLA-DRA, TNF |
| ECM-receptor interaction | 10/88 | 1.51E-05 | 2.24E-04 | VTN, ITGB4, ITGA1, FN1, GP1BA, CD36, COL6A5, COL9A2, AGRN, THBS1 |
| Type I diabetes mellitus | 7/43 | 2.79E-05 | 3.90E-04 | IL1A, IL1B, HLA-B, HLA-DPB1, LTA, HLA-DRA, TNF |
| JAK-STAT signaling pathway | 13/162 | 3.74E-05 | 4.95E-04 | IL10, IL15RA, CSF2, FHL1, IL13, IL19, IL2RG, IL27RA, IL5, IL2RA, IL2RB, STAT4, IL7R |
| AGE-RAGE signaling pathway in diabetic complications | 10/100 | 4.63E-05 | 5.80E-04 | IL1A, SMAD3, CXCL8, NOS3, MMP2, IL1B, SERPINE1, FN1, VEGFC, TNF |
| Pathways in cancer | 26/531 | 5.81E-05 | 6.46E-04 | CXCL8, GSTP1, PTGS2, IL2RG, RASGRP1, RASGRP3, STAT4, PIM2, IL15RA, ARNT2, SMAD3, MMP2, IL13, FN1, VEGFC, WNT9A, TRAF1, MMP9, NFKB2, NFKBIA, IL5, IL2RA, IL2RB, CCDC6, IL7R, BIRC3 |
| Intestinal immune network for IgA production | 7/48 | 5.82E-05 | 6.46E-04 | IL10, PIGR, IL15RA, CD40, IL5, HLA-DPB1, HLA-DRA |
| Protein digestion and absorption | 10/103 | 5.97E-05 | 6.46E-04 | DPP4, KCNK5, COL15A1, COL16A1, COL13A1, COL5A3, COL7A1, PRCP, COL9A2, COL6A5 |
| Th17 cell differentiation | 10/107 | 8.26E-05 | 8.55E-04 | NFKBIA, SMAD3, IRF4, IL1B, IL2RA, IL2RB, HLA-DPB1, HLA-DRA, IL2RG, IL27RA |
| Systemic lupus erythematosus | 11/135 | 1.28E-04 | 1.25E-03 | C3, IL10, H2BC12, CD40, H2AC6, H2BC5, H2BC11, H2BC4, HLA-DPB1, HLA-DRA, TNF |
| Th1 and Th2 cell differentiation | 9/92 | 1.31E-04 | 1.25E-03 | NFKBIA, IL5, IL2RA, IL13, IL2RB, STAT4, HLA-DPB1, HLA-DRA, IL2RG |
| Legionellosis | 7/57 | 1.78E-04 | 1.61E-03 | C3, NFKBIA, CXCL8, IL1B, TNF, TLR2, NFKB2 |
| Lipid and atherosclerosis | 14/215 | 1.82E-04 | 1.61E-03 | CD40, CXCL8, NOS3, POU2F2, TNF, MMP9, NFKBIA, IL1B, CCL5, CYP1A1, CCL3, OLR1, CD36, TLR2 |
| Graft-versus-host disease | 6/42 | 2.23E-04 | 1.90E-03 | IL1A, IL1B, HLA-B, HLA-DPB1, HLA-DRA, TNF |
| Tuberculosis | 12/180 | 4.20E-04 | 3.45E-03 | C3, IL10, CYP27B1, IL1A, CD74, IRAK2, IL1B, MRC1, HLA-DPB1, HLA-DRA, TNF, TLR2 |
| Measles | 10/139 | 6.94E-04 | 5.51E-03 | NFKBIA, IL1A, IL1B, OAS3, IL2RA, IL2RB, TNFAIP3, TLR7, IL2RG, TLR2 |
| Autoimmune thyroid disease | 6/53 | 8.05E-04 | 6.18E-03 | IL10, CD40, IL5, HLA-B, HLA-DPB1, HLA-DRA |
| Epstein-Barr virus infection | 12/202 | 1.16E-03 | 8.63E-03 | NFKBIA, FCER2, CD40, OAS3, HLA-B, HLA-DPB1, HLA-DRA, TNFAIP3, TNF, TLR2, RELB, NFKB2 |
| C-type lectin receptor signaling pathway | 8/104 | 1.53E-03 | 0.01 | NFKBIA, IL10, EGR2, IL1B, PTGS2, TNF, NFKB2, RELB |
| T cell receptor signaling pathway | 8/104 | 1.53E-03 | 0.01 | NFKBIA, IL10, CSF2, IL5, MAP3K8, TNF, RASGRP1, CARD11 |
| Arachidonic acid metabolism | 6/61 | 1.69E-03 | 0.01 | PLA2G4C, ALOX15, AKR1C3, PLA2G4A, PTGS2, PTGES |
| Necroptosis | 10/159 | 1.93E-03 | 0.01 | IL1A, H2AC6, IL1B, ALOX15, PLA2G4C, STAT4, TNFAIP3, PLA2G4A, TNF, BIRC3 |
| Transcriptional misregulation in cancer | 11/192 | 2.44E-03 | 0.02 | ARNT2, CD40, CSF2, NR4A3, CXCL8, WT1, NFKBIZ, IL2RB, TRAF1, MMP9, BIRC3 |
| Toxoplasmosis | 8/112 | 2.44E-03 | 0.02 | NFKBIA, IL10, CD40, HLA-DPB1, HLA-DRA, TNF, BIRC3, TLR2 |
| Fc epsilon RI signaling pathway | 6/68 | 2.95E-03 | 0.02 | CSF2, IL5, IL13, PLA2G4C, PLA2G4A, TNF |
| Apoptosis | 9/142 | 3.03E-03 | 0.02 | SPTA1, NFKBIA, DAB2IP, CTSH, TRAF1, SEPTIN4, PTPN13, TNF, BIRC3 |
| Influenza A | 10/172 | 3.41E-03 | 0.02 | NFKBIA, IL1A, CXCL8, IL1B, OAS3, CCL5, HLA-DPB1, HLA-DRA, TLR7, TNF |
| Focal adhesion | 11/201 | 3.47E-03 | 0.02 | SHC4, VTN, ITGB4, ITGA1, FN1, VEGFC, COL9A2, COL6A5, MYL9, THBS1, BIRC3 |
| Viral carcinogenesis | 11/203 | 3.74E-03 | 0.02 | C3, NFKBIA, H2BC12, EGR2, PKM, H2BC5, H2BC4, H2BC11, HLA-B, TRAF1, NFKB2 |
| Arginine and proline metabolism | 5/50 | 3.81E-03 | 0.02 | MAOA, NOS3, P4HA2, PYCR1, AGMAT |
| Ovarian steroidogenesis | 5/51 | 4.15E-03 | 0.02 | PLA2G4C, AKR1C3, CYP1A1, PLA2G4A, PTGS2 |
| Phagosome | 9/152 | 4.75E-03 | 0.02 | C3, MRC1, HLA-B, HLA-DPB1, HLA-DRA, OLR1, CD36, THBS1, TLR2 |
| Pertussis | 6/76 | 5.13E-03 | 0.03 | IL10, C3, IL1A, CXCL8, IL1B, TNF |
| Amoebiasis | 7/102 | 5.58E-03 | 0.03 | IL10, CSF2, CXCL8, IL1B, FN1, TNF, TLR2 |
| Neutrophil extracellular trap formation | 10/189 | 6.57E-03 | 0.03 | C3, H2BC12, CLCN4, H2AC6, H2BC5, H2BC11, H2BC4, GP1BA, TLR7, TLR2 |
| Chemokine signaling pathway | 10/192 | 7.31E-03 | 0.03 | SHC4, NFKBIA, CCL24, CXCL8, CCL5, CCL4, CXCR5, CCL3, CCR7, CCL1 |
| Mineral absorption | 5/60 | 8.29E-03 | 0.04 | MT2A, MT1G, TRPM6, ATOX1, MT1E |
| MAPK signaling pathway | 13/294 | 9.27E-03 | 0.04 | PLA2G4C, CACNA1B, VEGFC, PLA2G4A, TNF, RASGRP1, CACNA1H, RELB, RASGRP3, NFKB2, IL1A, IL1B, MAP3K8 |
| Glycine, serine and threonine metabolism | 4/40 | 9.49E-03 | 0.04 | MAOA, CBS, PSAT1, PHGDH |
| Bladder cancer | 4/41 | 0.01 | 0.05 | CXCL8, MMP2, THBS1, MMP9 |
| Tryptophan metabolism | 4/42 | 0.01 | 0.05 | IL4I1, MAOA, CYP1A1, ALDH8A1 |
| Proteoglycans in cancer | 10/205 | 0.01 | 0.05 | VTN, CTTN, MMP2, FN1, ANK2, WNT9A, TNF, MMP9, THBS1, TLR2 |
| NOD-like receptor signaling pathway | 9/181 | 0.01 | 0.06 | NFKBIA, CXCL8, IL1B, OAS3, CCL5, TNFAIP3, TNF, GBP4, BIRC3 |
| PI3K-Akt signaling pathway | 14/354 | 0.02 | 0.07 | ITGB4, NOS3, ITGA1, FN1, VEGFC, IL2RG, THBS1, VTN, IL2RA, IL2RB, COL9A2, COL6A5, IL7R, TLR2 |
| Cysteine and methionine metabolism | 4/50 | 0.02 | 0.08 | IL4I1, CBS, PSAT1, PHGDH |
| Hepatitis B | 8/162 | 0.02 | 0.08 | NFKBIA, EGR2, SMAD3, CXCL8, STAT4, TNF, MMP9, TLR2 |
| Linoleic acid metabolism | 3/29 | 0.02 | 0.09 | ALOX15, PLA2G4C, PLA2G4A |
| Antigen processing and presentation | 5/78 | 0.02 | 0.09 | CD74, HLA-B, HLA-DPB1, HLA-DRA, TNF |
| Coronavirus disease | 10/232 | 0.02 | 0.09 | C3, NFKBIA, ACE, CSF2, CXCL8, IL1B, OAS3, TLR7, TNF, TLR2 |
| Glycosaminoglycan biosynthesis | 4/53 | 0.02 | 0.09 | CHST7, CHPF, DSE, CHST1 |
| Insulin resistance | 6/108 | 0.03 | 0.09 | NFKBIA, PPP1R3C, NOS3, CD36, TNF, SLC27A2 |
| Fluid shear stress and atherosclerosis | 7/139 | 0.03 | 0.10 | IL1A, NOS3, MMP2, IL1B, GSTP1, TNF, MMP9 |
| Serotonergic synapse | 6/113 | 0.03 | 0.11 | MAOA, ALOX15, PLA2G4C, CACNA1B, PLA2G4A, PTGS2 |
| Shigellosis | 10/246 | 0.03 | 0.12 | C3, NFKBIA, CSF2, CXCL8, CTTN, TNIP1, IL1B, CCL5, MYL9, TNF |
| VEGF signaling pathway | 4/59 | 0.03 | 0.12 | NOS3, PLA2G4C, PLA2G4A, PTGS2 |
| Viral myocarditis | 4/60 | 0.04 | 0.12 | CD40, HLA-B, HLA-DPB1, HLA-DRA |
| African trypanosomiasis | 3/37 | 0.04 | 0.14 | IL10, IL1B, TNF |
| Cytosolic DNA-sensing pathway | 4/63 | 0.04 | 0.14 | NFKBIA, IL1B, CCL5, CCL4 |
| Small cell lung cancer | 5/92 | 0.04 | 0.14 | NFKBIA, FN1, TRAF1, PTGS2, BIRC3 |
| Primary immunodeficiency | 3/38 | 0.04 | 0.14 | CD40, IL2RG, IL7R |
| Platelet activation | 6/124 | 0.05 | 0.15 | PTGIR, NOS3, PLA2G4C, PLA2G4A, GP1BA, RASGRP1 |
| Human papillomavirus infection | 12/331 | 0.05 | 0.15 | VTN, PKM, ITGB4, ITGA1, HLA-B, FN1, COL9A2, WNT9A, COL6A5, PTGS2, TNF, THBS1 |
| TGF-beta signaling pathway | 5/94 | 0.05 | 0.15 | SMAD3, BAMBI, INHBA, TNF, THBS1 |
| Human cytomegalovirus infection | 9/225 | 0.05 | 0.15 | NFKBIA, CXCL8, IL1B, CCL5, CCL4, HLA-B, CCL3, PTGS2, TNF |
| Phenylalanine metabolism | 2/17 | 0.05 | 0.15 | IL4I1, MAOA |

**Supplementary Table S4** Pathway enrichment of the up-regulated IL-33 stimulated basophil cell signature

| Term | Overlap | P-value | Adjusted P-value | Genes |
| --- | --- | --- | --- | --- |
| TGF-beta signaling pathway | 5/94 | 2.38E-03 | 0.36 | TGIF1, TFDP1, BAMBI, THBS1, BMP6 |
| Inflammatory bowel disease | 4/65 | 3.87E-03 | 0.36 | IL4R, STAT4, NFKB1, IL18R1 |
| Pantothenate and CoA biosynthesis | 2/21 | 0.02 | 0.62 | VNN1, VNN3 |
| Rap1 signaling pathway | 6/210 | 0.02 | 0.62 | SIPA1L1, PDGFC, FPR1, PLCB1, THBS1, VEGFA |
| C-type lectin receptor signaling pathway | 4/104 | 0.02 | 0.62 | CBLB, NFKB1, NFKB2, RELB |
| Lipid and atherosclerosis | 6/215 | 0.02 | 0.62 | ABCA1, OLR1, PLCB1, SOD2, ABCG1, NFKB1 |
| Cortisol synthesis and secretion | 3/65 | 0.03 | 0.63 | CYP21A2, PLCB1, PDE8A |
| MAPK signaling pathway | 7/294 | 0.03 | 0.63 | PDGFC, NF1, DUSP16, NFKB1, RELB, VEGFA, NFKB2 |
| Neutrophil extracellular trap formation | 5/189 | 0.04 | 0.69 | HDAC4, AQP9, FPR1, PLCB1, NFKB1 |

**Supplementary Table S5** Pathway enrichment of the up-regulated IL-33 stimulated ILC2 cell signature

| Term | Overlap | P-value | Adjusted P-value | Genes |
| --- | --- | --- | --- | --- |
| Cytokine-cytokine receptor interaction | 14/295 | 6.04E-10 | 7.73E-08 | CCR1, CSF2, CCL3L1, IL26, CCL3L3, IL13, CSF2RB, IL17RB, IL4, IL5, IL2RA, TNFSF10, CCL3, TNFRSF8 |
| Fc epsilon RI signaling pathway | 7/68 | 7.63E-08 | 4.88E-06 | IL4, CSF2, FCER1G, IL5, IL13, ALOX5AP, PLA2G4A |
| IL-17 signaling pathway | 7/94 | 7.19E-07 | 3.07E-05 | IL4, CSF2, IL5, IL13, PTGS2, MAPK6, IL17RB |
| Hematopoietic cell lineage | 6/99 | 1.52E-05 | 3.99E-04 | IL4, CSF2, IL5, IL2RA, ITGA1, CD38 |
| Viral protein interaction with cytokine and cytokine receptor | 6/100 | 1.61E-05 | 3.99E-04 | CCR1, CCL3L1, CCL3L3, IL2RA, TNFSF10, CCL3 |
| T cell receptor signaling pathway | 6/104 | 2.02E-05 | 3.99E-04 | IL4, CSF2, IL5, CTLA4, MAP3K8, ICOS |
| Asthma | 4/31 | 2.18E-05 | 3.99E-04 | IL4, FCER1G, IL5, IL13 |
| Rheumatoid arthritis | 5/93 | 1.43E-04 | 2.28E-03 | CSF2, CCL3L1, CCL3L3, CCL3, CTLA4 |
| Autoimmune thyroid disease | 4/53 | 1.85E-04 | 2.64E-03 | IL4, IL5, CTLA4, GZMB |
| Human cytomegalovirus infection | 7/225 | 2.09E-04 | 2.67E-03 | CCR1, CCL3L1, CCL3L3, PTGER3, CCL3, TAP1, PTGS2 |
| JAK-STAT signaling pathway | 6/162 | 2.37E-04 | 2.76E-03 | IL4, CSF2, IL5, IL2RA, IL13, CSF2RB |
| Natural killer cell mediated cytotoxicity | 5/131 | 6.96E-04 | 7.42E-03 | CSF2, FCER1G, SH2D1A, TNFSF10, GZMB |
| Allograft rejection | 3/38 | 1.10E-03 | 0.01 | IL4, IL5, GZMB |
| Oxytocin signaling pathway | 5/154 | 1.43E-03 | 0.01 | RGS2, CD38, PLA2G4A, MYL6B, PTGS2 |
| Th1 and Th2 cell differentiation | 4/92 | 1.51E-03 | 0.01 | IL4, IL5, IL2RA, IL13 |
| Intestinal immune network for IgA production | 3/48 | 2.16E-03 | 0.02 | IL4, IL5, ICOS |
| Pathways in cancer | 9/531 | 2.20E-03 | 0.02 | IL4, IL5, IL2RA, IL13, PTGER3, CSF2RB, PIM2, RASGRP2, PTGS2 |
| Chagas disease | 4/102 | 2.20E-03 | 0.02 | GNA15, CCL3L1, CCL3L3, CCL3 |
| Toll-like receptor signaling pathway | 4/104 | 2.36E-03 | 0.02 | CCL3L1, CCL3L3, CCL3, MAP3K8 |
| Chemokine signaling pathway | 5/192 | 3.73E-03 | 0.02 | CCR1, CCL3L1, CCL3L3, CCL3, RASGRP2 |
| Mineral absorption | 3/60 | 4.08E-03 | 0.02 | MT1A, MT1F, MT1E |
| Inflammatory bowel disease | 3/65 | 5.11E-03 | 0.03 | IL4, IL5, IL13 |
| Nicotinate and nicotinamide metabolism | 2/35 | 0.02 | 0.08 | QPRT, CD38 |
| NOD-like receptor signaling pathway | 4/181 | 0.02 | 0.09 | GBP5, GBP2, GBP1, GBP4 |
| Primary immunodeficiency | 2/38 | 0.02 | 0.09 | TAP1, ICOS |
| Th17 cell differentiation | 3/107 | 0.02 | 0.10 | IL4, IRF4, IL2RA |
| TNF signaling pathway | 3/112 | 0.02 | 0.11 | CSF2, MAP3K8, PTGS2 |
| Proteasome | 2/46 | 0.03 | 0.12 | PSMD1, PSMB8 |
| Lipid and atherosclerosis | 4/215 | 0.03 | 0.12 | CCL3L1, CCL3L3, TNFSF10, CCL3 |
| Platelet activation | 3/124 | 0.03 | 0.12 | FCER1G, PLA2G4A, RASGRP2 |
| Ovarian steroidogenesis | 2/51 | 0.03 | 0.13 | PLA2G4A, PTGS2 |
| Regulation of lipolysis in adipocytes | 2/55 | 0.04 | 0.14 | PTGER3, PTGS2 |
| Measles | 3/139 | 0.04 | 0.15 | IL2RA, MX1, RAB9A |
| VEGF signaling pathway | 2/59 | 0.04 | 0.15 | PLA2G4A, PTGS2 |
| Apoptosis | 3/142 | 0.04 | 0.15 | TNFSF10, GZMB, CSF2RB |
| Arachidonic acid metabolism | 2/61 | 0.04 | 0.15 | PLA2G4A, PTGS2 |
| Alzheimer disease | 5/369 | 0.05 | 0.17 | TUBB6, PSEN2, PSMD1, UQCRC2, PTGS2 |

**Supplementary Table S6** Pathway enrichment of the up-regulated IL-33 stimulated HUVEC signature

| Term | Overlap | P-value | Adjusted P-value | Genes |
| --- | --- | --- | --- | --- |
| TNF signaling pathway | 24/112 | 1.37E-23 | 2.74E-21 | CXCL6, CSF2, JAG1, IL15, CCL20, TNFAIP3, CXCL1, TRAF1, PTGS2, CXCL2, NFKB1, CX3CL1, CXCL5, ICAM1, NFKBIA, CXCL10, IL6, IL1B, IRF1, CCL2, MAP3K8, BIRC2, IL18R1, BIRC3 |
| Cytokine-cytokine receptor interaction | 32/295 | 1.52E-21 | 1.52E-19 | CXCL6, CSF3, CSF2, EBI3, TNFRSF11B, CSF2RB, CXCL1, CXCL2, CX3CL1, CXCL5, CCL8, CCL7, TNFSF10, CCL2, IL32, IL15RA, TGFB2, TNFSF15, IL15, CCL20, TGFB3, IFNGR2, TNFRSF9, IL1A, CXCL10, IL6, BMP2, CXCL11, IL1B, LTB, IL7R, IL18R1 |
| IL-17 signaling pathway | 17/94 | 1.01E-15 | 6.74E-14 | CXCL6, CSF3, CSF2, CCL20, TNFAIP3, CXCL1, PTGS2, CXCL2, CXCL5, NFKB1, NFKBIA, CXCL10, IL6, CCL7, IL1B, CCL2, IKBKE |
| NF-kappa B signaling pathway | 17/104 | 5.94E-15 | 2.97E-13 | DDX58, TNFAIP3, CXCL1, TRAF1, TICAM1, PTGS2, CXCL2, NFKB1, ICAM1, NFKB2, RELB, NFKBIA, PLAU, IL1B, LTB, BIRC2, BIRC3 |
| Rheumatoid arthritis | 15/93 | 2.99E-13 | 1.20E-11 | CXCL6, TGFB2, CSF2, IL15, CCL20, TGFB3, CXCL1, CXCL2, CXCL5, ICAM1, IL1A, IL6, IL1B, CCL2, LTB |
| Viral protein interaction with cytokine and cytokine receptor | 14/100 | 1.39E-11 | 4.63E-10 | CXCL6, CCL20, CXCL1, CXCL2, CX3CL1, CXCL5, CXCL10, CXCL11, IL6, CCL8, CCL7, TNFSF10, CCL2, IL18R1 |
| Influenza A | 16/172 | 2.57E-10 | 7.35E-09 | DDX58, IFNGR2, MX1, TICAM1, NFKB1, ICAM1, NFKBIA, IFIH1, CXCL10, IL1A, IL6, CDK6, IL1B, TNFSF10, CCL2, IKBKE |
| NOD-like receptor signaling pathway | 16/181 | 5.49E-10 | 1.37E-08 | TNFAIP3, CXCL1, TICAM1, CXCL2, NFKB1, NFKBIA, PANX1, IL6, IL1B, CCL2, GBP2, IKBKE, GBP1, BIRC2, GBP4, BIRC3 |
| Pathways in cancer | 25/531 | 5.41E-09 | 1.20E-07 | CSF2RB, LAMC2, PTGS2, ETS1, HEY1, ITGAV, NKX3-1, STAT5A, IL15RA, TGFB2, JAG1, SMAD3, IL15, TGFB3, IFNGR2, TRAF1, NFKB1, NFKB2, NFKBIA, IL6, BMP2, CDK6, IL7R, BIRC2, BIRC3 |
| Human T-cell leukemia virus 1 infection | 16/219 | 8.77E-09 | 1.75E-07 | STAT5A, IL15RA, TGFB2, CSF2, SMAD3, IL15, TGFB3, HLA-B, ETS1, NFKB1, ICAM1, RELB, NFKB2, NFKBIA, IL6, MSX1 |
| AGE-RAGE signaling pathway in diabetic complications | 11/100 | 3.00E-08 | 5.46E-07 | STAT5A, IL1A, TGFB2, IL6, SMAD3, TGFB3, IL1B, CCL2, F3, NFKB1, ICAM1 |
| Lipid and atherosclerosis | 15/215 | 4.78E-08 | 7.96E-07 | CXCL1, TICAM1, POU2F2, SOD2, CXCL2, NFKB1, ICAM1, NFKBIA, IL6, IL1B, TNFSF10, OLR1, CCL2, IKBKE, ABCG1 |
| Inflammatory bowel disease | 9/65 | 7.37E-08 | 1.13E-06 | IL1A, TGFB2, IL6, SMAD3, TGFB3, IFNGR2, IL1B, NFKB1, IL18R1 |
| Measles | 12/139 | 1.07E-07 | 1.54E-06 | STAT5A, NFKBIA, IFIH1, IL1A, IL6, CDK6, DDX58, IL1B, MX1, TNFAIP3, IKBKE, NFKB1 |
| Epstein-Barr virus infection | 14/202 | 1.47E-07 | 1.96E-06 | DDX58, HLA-B, TAP1, TNFAIP3, NFKB1, ICAM1, RELB, NFKB2, NFKBIA, CXCL10, IL6, CDK6, NFKBIE, IKBKE |
| Chemokine signaling pathway | 13/192 | 5.38E-07 | 6.73E-06 | CXCL6, CCL20, CXCL1, CXCL2, NFKB1, CX3CL1, CXCL5, NFKBIA, CXCL10, CXCL11, CCL8, CCL7, CCL2 |
| Small cell lung cancer | 9/92 | 1.52E-06 | 1.78E-05 | NFKBIA, CDK6, LAMC2, ITGAV, TRAF1, PTGS2, BIRC2, NFKB1, BIRC3 |
| Malaria | 7/50 | 2.00E-06 | 2.22E-05 | CSF3, TGFB2, IL6, TGFB3, IL1B, CCL2, ICAM1 |
| Osteoclast differentiation | 10/127 | 2.92E-06 | 2.97E-05 | NFKBIA, IL1A, TGFB2, IFNGR2, IL1B, TNFRSF11B, SQSTM1, NFKB1, NFKB2, RELB |
| Necroptosis | 11/159 | 3.31E-06 | 2.97E-05 | STAT5A, IL1A, IFNGR2, IL1B, PLA2G4C, TNFSF10, TNFAIP3, TICAM1, SQSTM1, BIRC2, BIRC3 |
| Pertussis | 8/76 | 3.36E-06 | 2.97E-05 | CXCL6, IL1A, IL6, IL1B, IRF1, TICAM1, CXCL5, NFKB1 |
| Amoebiasis | 9/102 | 3.60E-06 | 2.97E-05 | TGFB2, IL6, CSF2, TGFB3, IL1B, CXCL1, LAMC2, CXCL2, NFKB1 |
| Chagas disease | 9/102 | 3.60E-06 | 2.97E-05 | NFKBIA, TGFB2, IL6, TGFB3, IFNGR2, IL1B, CCL2, TICAM1, NFKB1 |
| Kaposi sarcoma-associated herpesvirus infection | 12/193 | 3.61E-06 | 2.97E-05 | NFKBIA, IL6, CSF2, CDK6, HLA-B, CXCL1, PTGS2, TICAM1, IKBKE, CXCL2, NFKB1, ICAM1 |
| Leishmaniasis | 8/77 | 3.71E-06 | 2.97E-05 | NFKBIA, IL1A, TGFB2, TGFB3, IFNGR2, IL1B, PTGS2, NFKB1 |
| Hepatitis B | 11/162 | 3.97E-06 | 3.03E-05 | STAT5A, NFKBIA, IFIH1, TGFB2, IL6, SMAD3, TGFB3, DDX58, TICAM1, IKBKE, NFKB1 |
| C-type lectin receptor signaling pathway | 9/104 | 4.24E-06 | 3.03E-05 | NFKBIA, IL6, IL1B, IRF1, PTGS2, IKBKE, NFKB1, NFKB2, RELB |
| Toll-like receptor signaling pathway | 9/104 | 4.24E-06 | 3.03E-05 | NFKBIA, CXCL10, CXCL11, IL6, IL1B, MAP3K8, TICAM1, IKBKE, NFKB1 |
| Coronavirus disease | 13/232 | 4.46E-06 | 3.08E-05 | CSF3, CSF2, DDX58, MX1, NFKB1, IFIH1, NFKBIA, CXCL10, IL6, IL1B, CCL2, IKBKE, CFB |
| Legionellosis | 7/57 | 4.93E-06 | 3.29E-05 | NFKBIA, IL6, IL1B, CXCL1, CXCL2, NFKB1, NFKB2 |
| Cytosolic DNA-sensing pathway | 7/63 | 9.71E-06 | 6.26E-05 | NFKBIA, CXCL10, IL6, DDX58, IL1B, IKBKE, NFKB1 |
| Cellular senescence | 10/156 | 1.81E-05 | 1.13E-04 | IL1A, TGFB2, IL6, SMAD3, CDK6, TGFB3, HLA-B, ETS1, SQSTM1, NFKB1 |
| FoxO signaling pathway | 9/131 | 2.76E-05 | 1.67E-04 | TGFB2, IL6, SMAD3, BCL6, TGFB3, TNFSF10, FBXO32, IL7R, SOD2 |
| Chronic myeloid leukemia | 7/76 | 3.37E-05 | 1.98E-04 | STAT5A, NFKBIA, TGFB2, SMAD3, CDK6, TGFB3, NFKB1 |
| Th17 cell differentiation | 8/107 | 4.23E-05 | 2.42E-04 | STAT5A, NFKBIA, IL6, SMAD3, IFNGR2, IL1B, NFKBIE, NFKB1 |
| Toxoplasmosis | 8/112 | 5.87E-05 | 3.26E-04 | NFKBIA, TGFB2, TGFB3, IFNGR2, LAMC2, NFKB1, BIRC2, BIRC3 |
| Human cytomegalovirus infection | 11/225 | 8.44E-05 | 4.56E-04 | NFKBIA, IL6, CDK6, IL1B, HLA-B, TAP1, CCL2, ITGAV, PTGS2, CX3CL1, NFKB1 |
| JAK-STAT signaling pathway | 9/162 | 1.44E-04 | 7.55E-04 | STAT5A, IL15RA, CSF3, IL6, CSF2, IL15, IFNGR2, CSF2RB, IL7R |
| Hippo signaling pathway | 9/163 | 1.50E-04 | 7.71E-04 | TGFB2, BMP2, SMAD3, TGFB3, WWC1, BIRC2, SAV1, BIRC3, NKD2 |
| Human papillomavirus infection | 13/331 | 1.78E-04 | 8.92E-04 | JAG1, MX1, HLA-B, TNC, LAMC2, PTGS2, TICAM1, NFKB1, CDK6, HEY1, IRF1, ITGAV, IKBKE |
| RIG-I-like receptor signaling pathway | 6/70 | 1.85E-04 | 9.03E-04 | IFIH1, NFKBIA, CXCL10, DDX58, IKBKE, NFKB1 |
| Fluid shear stress and atherosclerosis | 8/139 | 2.64E-04 | 1.26E-03 | IL1A, SDC4, IL1B, CCL2, ITGAV, SQSTM1, NFKB1, ICAM1 |
| Transcriptional misregulation in cancer | 9/192 | 5.05E-04 | 2.35E-03 | IL6, CSF2, BCL6, PLAU, NFKBIZ, TRAF1, NFKB1, BIRC2, BIRC3 |
| Hepatitis C | 8/157 | 5.97E-04 | 2.71E-03 | NFKBIA, CXCL10, CDK6, DDX58, MX1, TICAM1, IKBKE, NFKB1 |
| Th1 and Th2 cell differentiation | 6/92 | 8.10E-04 | 3.60E-03 | STAT5A, NFKBIA, JAG1, IFNGR2, NFKBIE, NFKB1 |
| MAPK signaling pathway | 11/294 | 8.32E-04 | 3.62E-03 | EFNA1, IL1A, TGFB2, TGFB3, IL1B, PLA2G4C, MAP3K8, DUSP16, NFKB1, RELB, NFKB2 |
| African trypanosomiasis | 4/37 | 9.49E-04 | 4.03E-03 | IL6, IL1B, F2RL1, ICAM1 |
| Herpes simplex virus 1 infection | 15/498 | 9.68E-04 | 4.03E-03 | DDX58, IFNGR2, HLA-B, TAP1, TICAM1, POU2F2, NFKB1, IFIH1, NFKBIA, IL6, IL1B, CCL2, IKBKE, BIRC2, BIRC3 |
| Hematopoietic cell lineage | 6/99 | 1.19E-03 | 4.85E-03 | IL1A, CSF3, IL6, CSF2, IL1B, IL7R |
| Tuberculosis | 8/180 | 1.45E-03 | 5.78E-03 | IL1A, TGFB2, IL6, IRAK2, TGFB3, IFNGR2, IL1B, NFKB1 |
| Graft-versus-host disease | 4/42 | 1.53E-03 | 6.02E-03 | IL1A, IL6, IL1B, HLA-B |
| Apoptosis | 7/142 | 1.58E-03 | 6.08E-03 | NFKBIA, TNFSF10, CSF2RB, TRAF1, NFKB1, BIRC2, BIRC3 |
| Pancreatic cancer | 5/76 | 2.13E-03 | 8.03E-03 | TGFB2, SMAD3, CDK6, TGFB3, NFKB1 |
| Intestinal immune network for IgA production | 4/48 | 2.52E-03 | 9.35E-03 | IL15RA, IL6, IL15, ICOSLG |
| ECM-receptor interaction | 5/88 | 4.02E-03 | 0.01 | SDC4, TNC, LAMC2, ITGAV, CD47 |
| PD-L1 expression and PD-1 checkpoint pathway in cancer | 5/89 | 4.22E-03 | 0.02 | NFKBIA, IFNGR2, TICAM1, NFKBIE, NFKB1 |
| Yersinia infection | 6/137 | 6.01E-03 | 0.02 | NFKBIA, IL6, IL1B, CCL2, TICAM1, NFKB1 |
| T cell receptor signaling pathway | 5/104 | 8.11E-03 | 0.03 | NFKBIA, CSF2, MAP3K8, NFKBIE, NFKB1 |
| Epithelial cell signaling in Helicobacter pylori infection | 4/70 | 9.71E-03 | 0.03 | NFKBIA, CXCL1, CXCL2, NFKB1 |
| Salmonella infection | 8/249 | 0.01 | 0.03 | NFKBIA, TUBB2B, IL6, IL1B, TNFSF10, NFKB1, BIRC2, BIRC3 |
| Viral carcinogenesis | 7/203 | 0.01 | 0.04 | STAT5A, NFKBIA, CDK6, HLA-B, TRAF1, NFKB1, NFKB2 |
| Ferroptosis | 3/41 | 0.01 | 0.04 | SLC11A2, SLC7A11, SAT1 |
| Type I diabetes mellitus | 3/43 | 0.01 | 0.05 | IL1A, IL1B, HLA-B |
| Other types of O-glycan biosynthesis | 3/47 | 0.02 | 0.06 | ST6GAL1, B4GALT1, GALNT4 |
| Complement and coagulation cascades | 4/85 | 0.02 | 0.06 | SERPINB2, PLAU, F3, CFB |
| Natural killer cell mediated cytotoxicity | 5/131 | 0.02 | 0.06 | CSF2, IFNGR2, TNFSF10, HLA-B, ICAM1 |
| Hypertrophic cardiomyopathy | 4/90 | 0.02 | 0.07 | TGFB2, IL6, TGFB3, ITGAV |
| TGF-beta signaling pathway | 4/94 | 0.03 | 0.08 | TGFB2, BMP2, SMAD3, TGFB3 |
| PI3K-Akt signaling pathway | 9/354 | 0.03 | 0.08 | EFNA1, CSF3, IL6, CDK6, TNC, LAMC2, ITGAV, IL7R, NFKB1 |
| Shigellosis | 7/246 | 0.03 | 0.08 | NFKBIA, CSF2, TNIP1, IL1B, TIFA, SQSTM1, NFKB1 |
| Prostate cancer | 4/97 | 0.03 | 0.08 | NFKBIA, PLAU, NFKB1, NKX3-1 |
| Inflammatory mediator regulation of TRP channels | 4/98 | 0.03 | 0.08 | HRH1, IL1B, PLA2G4C, F2RL1 |
| Pathogenic Escherichia coli infection | 6/197 | 0.03 | 0.08 | NFKBIA, TUBB2B, IL6, IL1B, TNFSF10, NFKB1 |
| Cell adhesion molecules | 5/148 | 0.03 | 0.09 | SDC4, HLA-B, ITGAV, ICOSLG, ICAM1 |
| MicroRNAs in cancer | 8/310 | 0.03 | 0.09 | EFNA1, TGFB2, CDK6, PLAU, TNC, ZFPM2, PTGS2, NFKB1 |
| Phagosome | 5/152 | 0.04 | 0.09 | TUBB2B, HLA-B, TAP1, OLR1, ITGAV |
| Arachidonic acid metabolism | 3/61 | 0.04 | 0.09 | PLA2G4C, PTGS2, CBR3 |
| Insulin resistance | 4/108 | 0.04 | 0.10 | NFKBIA, IL6, GFPT2, NFKB1 |
| Acute myeloid leukemia | 3/67 | 0.05 | 0.12 | STAT5A, CSF2, NFKB1 |
| Adipocytokine signaling pathway | 3/69 | 0.05 | 0.12 | NFKBIA, NFKBIE, NFKB1 |
| Renal cell carcinoma | 3/69 | 0.05 | 0.12 | TGFB2, TGFB3, ETS1 |

**Supplementary Table S7** Overlaps across all IL-33 stimulated gene signatures and across TAC 1-3 gene signatures.

| **IL-33 stimulated Mast cell up-regulated gene signature (418 genes)** | | | | | |
| --- | --- | --- | --- | --- | --- |
|  |  |  | |  |  |
|  | **Compared with:** |  | **Overlapping genes** | **Out of** | **Overlap percentage** |
|  | **IL-33 stimulated mast cell down-regulated signature** |  | 0 | 418 | 0.00% |
|  | **IL-33 stimulated basophil cell up-regulated signature** |  | 11 | 418 | 2.63% |
|  | **IL-33 stimulated basophil cell down-regulated signature** |  | 4 | 418 | 0.96% |
|  | **IL-33 stimulated ILC2 cell up-regulated signature** |  | 21 | 418 | 5.02% |
|  | **IL-33 stimulated ILC2 cell down-regulated signature** |  | 1 | 418 | 0.24% |
|  | **IL-33 stimulated HUVEC cell up-regulated signature** |  | 38 | 418 | 9.09% |
|  | **IL-33 stimulated HUVEC cell down-regulated signature** |  | 4 | 418 | 0.96% |
|  | **TAC 1** |  | 1 | 418 | 0.24% |
|  | **TAC 2** |  | 2 | 418 | 0.48% |
|  | **TAC 3** |  | 0 | 418 | 0.00% |
|  |  |  | |  |  |
| **IL-33 stimulated Mast cell down-regulated gene signature (353 genes)** | | | | | |
|  |  |  | |  |  |
|  | **Compared with:** |  | **Overlapping genes** | **Out of** | **Overlap percentage** |
|  | **IL-33 stimulated basophil cell up-regulated signature** |  | 4 | 353 | 1.13% |
|  | **IL-33 stimulated basophil cell down-regulated signature** |  | 2 | 353 | 0.57% |
|  | **IL-33 stimulated ILC2 cell up-regulated signature** |  | 1 | 353 | 0.28% |
|  | **IL-33 stimulated ILC2 cell down-regulated signature** |  | 5 | 353 | 1.42% |
|  | **IL-33 stimulated HUVEC cell up-regulated signature** |  | 1 | 353 | 0.28% |
|  | **IL-33 stimulated HUVEC cell down-regulated signature** |  | 4 | 353 | 1.13% |
|  | **TAC 1** |  | 0 | 353 | 0.00% |
|  | **TAC 2** |  | 1 | 353 | 0.28% |
|  | **TAC 3** |  | 1 | 353 | 0.28% |
|  | | | | | |
| **IL-33 stimulated basophil cell up-regulated gene signature (197 genes)** | | | | | |
|  |  |  | |  |  |
|  | **Compared with:** |  | **Overlapping genes** | **Out of** | **Overlap percentage** |
|  | **IL-33 stimulated basophil cell down-regulated signature** |  | 0 | 197 | 0.00% |
|  | **IL-33 stimulated ILC2 cell up-regulated signature** |  | 1 | 197 | 0.51% |
|  | **IL-33 stimulated ILC2 cell down-regulated signature** |  | 0 | 197 | 0.00% |
|  | **IL-33 stimulated HUVEC cell up-regulated signature** |  | 13 | 197 | 6.60% |
|  | **IL-33 stimulated HUVEC cell down-regulated signature** |  | 0 | 197 | 0.00% |
|  | **TAC 1** |  | 1 | 197 | 0.51% |
|  | **TAC 2** |  | 3 | 197 | 1.52% |
|  | **TAC 3** |  | 0 | 197 | 0.00% |
|  |  |  |  |  |  |
| **IL-33 stimulated basophil cell down-regulated gene signature (59 genes)** | | | | | |
|  |  |  | |  |  |
|  | **Compared with:** |  | **Overlapping genes** | **Out of** | **Overlap percentage** |
|  | **IL-33 stimulated ILC2 cell up-regulated signature** |  | 4 | 59 | 6.78% |
|  | **IL-33 stimulated ILC2 cell down-regulated signature** |  | 0 | 59 | 0.00% |
|  | **IL-33 stimulated HUVEC cell up-regulated signature** |  | 3 | 59 | 5.08% |
|  | **IL-33 stimulated HUVEC cell down-regulated signature** |  | 3 | 59 | 5.08% |
|  | **TAC 1** |  | 1 | 59 | 1.69% |
|  | **TAC 2** |  | 2 | 59 | 3.39% |
|  | **TAC 3** |  | 0 | 59 | 0.00% |
|  |  |  |  |  |  |
|  | | | | | |
| **IL-33 stimulated ILC2 cell up-regulated gene signature (107 genes)** | | | | | |
|  |  |  | |  |  |
|  | **Compared with:** |  | **Overlapping genes** | **Out of** | **Overlap percentage** |
|  | **IL-33 stimulated ILC2 cell down-regulated signature** |  | 0 | 107 | 0.00% |
|  | **IL-33 stimulated HUVEC cell up-regulated signature** |  | 15 | 107 | 14.02% |
|  | **IL-33 stimulated HUVEC cell down-regulated signature** |  | 0 | 107 | 0.00% |
|  | **TAC 1** |  | 0 | 107 | 0.00% |
|  | **TAC 2** |  | 1 | 107 | 0.93% |
|  | **TAC 3** |  | 0 | 107 | 0.00% |
|  |  |  |  |  |  |
| **IL-33 stimulated ILC2 cell down-regulated gene signature (32 genes)** | | | | | |
|  |  |  | |  |  |
|  | **Compared with:** |  | **Overlapping genes** | **Out of** | **Overlap percentage** |
|  | **IL-33 stimulated HUVEC cell up-regulated signature** |  | 1 | 32 | 3.13% |
|  | **IL-33 stimulated HUVEC cell down-regulated signature** |  | 0 | 32 | 0.00% |
|  | **TAC 1** |  | 0 | 32 | 0.00% |
|  | **TAC 2** |  | 0 | 32 | 0.00% |
|  | **TAC 3** |  | 1 | 32 | 3.13% |
|  |  |  |  |  |  |
| **IL-33 stimulated HUVEC cell up-regulated gene signature (238 genes)** | | | | | |
|  |  |  | |  |  |
|  | **Compared with:** |  | **Overlapping genes** | **Out of** | **Overlap percentage** |
|  | **IL-33 stimulated HUVEC cell down-regulated signature** |  | 0 | 238 | 0.00% |
|  | **TAC 1** |  | 0 | 238 | 0.00% |
|  | **TAC 2** |  | 2 | 238 | 0.84% |
|  | **TAC 3** |  | 0 | 238 | 0.00% |
|  |  |  |  |  |  |
| **IL-33 stimulated HUVEC cell down-regulated gene signature (61 genes)** | | | | | |
|  |  |  | |  |  |
|  | **Compared with:** |  | **Overlapping genes** | **Out of** | **Overlap percentage** |
|  | **TAC 1** |  | 0 | 61 | 0.00% |
|  | **TAC 2** |  | 0 | 61 | 0.00% |
|  | **TAC 3** |  | 0 | 61 | 0.00% |

**Supplementary Table S8** Venn diagram tables for up and down IL-33 gene signatures

**A** **Venn diagram tables of all up-regulated IL-33 up-regulated gene signatures**

Numbers of genes inputted

| **List names** | **number of elements** | **number of unique elements** |
| --- | --- | --- |
| Basophil | 197 | 197 |
| HUVEC | 238 | 238 |
| ILC2 | 107 | 107 |
| Mast cell | 418 | 418 |
| **Overall number of unique elements** | | **870** |

Venn diagram table

| Names | total | elements |
| --- | --- | --- |
| Basophil HUVEC Mast cell | 3 | NFKB2, RELB, OLR1 |
| HUVEC ILC2 Mast cell | 5 | MAP3K8, NFKBIZ, PTGS2, GBP4, CSF2 |
| Basophil HUVEC ILC2 | 1 | GBP2 |
| Basophil Mast cell | 8 | FEZ1, THBS1, STAT4, BAMBI, TGFBR3, PCSK5, RASL11A, MUC20 |
| ILC2 Mast cell | 16 | IRF4, IL2RA, MT1E, CD74, ITGA1, IL5, TMEM64, PLA2G4A, SLC27A2, IL17RB, LRIG1, CCL3, TNFRSF8, IL13, PIM2, BATF |
| HUVEC Mast cell | 30 | C15orf48, EBI3, IRAK2, SMAD3, TNFRSF9, PDLIM4, TRAF1, TNIP1, IL7R, MT1G, ZFPM2, ZC3H12A, IL1A, TNFAIP2, IL32, HLA-B, LTB, DSE, PLA2G4C, IL1B, CD200, IL4I1, ST6GAL1, NFKBIA, GCH1, IL15RA, SLC7A11, POU2F2, TNFAIP3, BIRC3 |
| Basophil HUVEC | 9 | SOD2, ABCG1, TNFAIP6, IFIH1, NFKB1, MARCH3, DUSP16, NINJ1, IL18R1 |
| HUVEC ILC2 | 9 | RGS2, TNFSF10, GBP1, TAP1, MX1, IL8, ELL2, TRIB1, CSF2RB |
| Mast cell | 356 | RCN3, ACE, AC011611.3, MMP2, ATOX1, H2BC4, KIF26B, PRCP, SLCO4A1, CNTNAP1, H2BC12, MIR3142HG, IL19, IGLV3-21, NMB, LINC01134, SGPP2, PXYLP1, AC245100.7, COL5A3, AC083837.1, GP1BA, MMP7, RDH10, P4HA2, DYRK3, EDIL3, TPSG1, NAMPTP1, NETO2, MREG, MARCKS, CNR2, TNS3, LINC02690, OCIAD2, AC107959.3, AL160408.3, CDH13, SERPINF1, CYP4F22, B4GALNT1, TNFSF4, LRFN5, GADL1, INHBA, TGM2, LIMS2, TYMSOS, IL10, AGRN, COL9A2, HIVEP2, AC007278.2, RGS3, TRPM6, STARD10, INA, SYNGR3, RASGRP1, CYP27B1, CARD11, PSD, SSTR2, CFAP46, SCG2, IGLV2-18, KIAA1958, SORD2P, ITGB4, PGAP4, DPP4, MT1L, MEX3A, CHST1, XIRP1, COL15A1, MAOA, TTC39A, AQP3, DAB2IP, C3, TLR7, PSAT1, VWA8, LAMP3, ALDH8A1, SYT12, PRG2, AL031283.1, FBXL19-AS1, MRAP2, CD36, HLA-DPB1, P2RX5-TAX1BP3, LINC02694, SLC22A31, NTN1, LINC02068, AC099552.1, CLCN4, HLA-DRA, KLF5, BCL9, LINC01215, GPR50, KCNH2, PDE4C, CPNE3, DNPH1, SYPL2, CYP1A1, NMNAT3, CD40, TNFRSF1B, COL6A5, RYR1, SEPTIN4, RAI2, RGL1, TMEM217, PIGR, NT5DC2, SEC11C, CYTIP, IGLV3-19, MYDGF, CCDC102A, AL137060.3, SPTA1, PRSS23, CHST7, H2BC5, GLIS2, NACAD, PTGES, MTHFD1L, GCKR, COL7A1, COLGALT2, CD81, MIR155HG, ALOX15, CHGA, CACNA1B, RASGRP3, AC034199.1, GDF11, CCL1, AL133520.1, KCNK13, FIRRE, HLF, H2BC11, NOS3, GSTP1, LINC01285, CTXN1, MMP2-AS1, CXCL8, AC120498.6, RIPOR3, MGAT5B, AC007384.1, GPRC5C, COL16A1, GOLGA2P10, IL27RA, CD70, KISS1R, SBK1, FHL1, FN1, SLAMF7, MFSD12, LINC00475, SORCS2, AC010247.1, TCERG1L, TMEM198, AC114977.1, AC124798.1, RGS9, CDHR1, TSPAN18, ZCCHC12, SAMD15, TNFRSF18, TMEM25, SFRP2, NUAK1, RRAD, SERPINE1, WT1, AHRR, CCDC6, PEAK1, GJA4, CCL5, ADAM8, EDARADD, PIEZO2, OAS3, NAV3, ABTB2, SPAG1, C1orf53, MT1DP, TNF, MFSD2A, TLL2, LOXL4, DIXDC1, TIGIT, AC007278.1, SLC16A9, UBE2E2, EGR2, NEDD4L, MYL9, CACNA1H, AC026310.1, EPOP, VWCE, MARCKSL1, ANK2, FERMT1, VEGFC, PKM, KIRREL3, FCER2, ZFPM1, AC078785.1, ENPP1, IGLV3-17, TMEM120A, LRG1, ASIC1, HAPLN3, BX255923.2, PTGES3L, AL445524.1, ROBO1, LINC02605, TTC39C, PHGDH, IL34, CRTAC1, CYP7B1, ROR1-AS1, LHFPL6, SHISA2, PPP1R3C, H2AC6, TMEM176A, CBS, WNT9A, CGN, VTN, SHC4, GCGR, FCRLA, P4HB, SLCO5A1, CCL4, PYCR1, MGP, FKBP5, LST1, AKR1C3, SPARC, FAM186B, SDF2L1, CTSH, MAP7D2, TNFSF9, GPR68, IGFBP4, TJP1, DLEU2, KCNK5, BAALC, LTA, TRIM9, BCAR3, TLR2, ZNF804A, SPATC1, GPC6, AC110611.2, ZBTB7C, MMP19, EFCAB12, QPCT, GAS6, LINC00892, NR4A3, AL645608.8, FRMD4A, AGMAT, PTGIR, MUC4, MT2A, AC034102.1, MIAT, TNFSF14, ROR1, IGFL2, SEMA4B, TENT5A, NME1, DLEU1, MRC1, PCSK9, ADGRA3, CHPF, IL2RB, ACHE, PTPN13, ARHGAP23, SERPIND1, TP53RK, CTTN, SLC16A1, NEK10, MT2P1, CA12, PKMP1, COL13A1, REEP2, APOC1, SCN9A, TMEM176B, IL18RAP, FLVCR2, CCL24, PIFO, CCR7, C1QTNF1, AC093503.2, H1-2, MAMLD1, ASB2, N4BP3, TINCR, WFS1, S100A9, ARNT2, MMP9, PDIA5, OLFM2, IL2RG, WLS, MMP11, CXCR5, MYO1E |
| Basophil | 176 | C2orf55, HIVEP1, SLA2, LOC728431, ENG, CXCR4, FLJ35776, LOC100133930, LOC441376, RNU4-2, PRDM8, LRRC6, ARRDC2, LOC644879, AQP9, MAGED4B, MRPL1, B3GAT1, FLJ39653, MYOM1, HDAC4, LIPN, SLC23A2, PLCB1, HIST1H2BG, CMIP, PTGDR, TFDP1, SNORA7B, HIST2H4A, EVI2A, LOC388969, VNN1, LQK1, LOC730092, GDPD3, CYP21A2, ABCA1, HIST2H4B, NFE2L3, C3AR1, LOC642567, FNDC3A, D2HGDH, APOLD1, PEX11G, RBBP8, GHRL, CALCRL, LOC653560, MOCOS, DUOX2, PLD6, FTHL11, LOC729009, TTYH2, PILRA, HIST1H2AC, CDKN2AIP, EXD3, AMDHD1, PLA2G7, FLJ77644, LYSMD2, ZNF585A, FAM43A, LOH12CR1, AHI1, PERP, BRE, IFIT2, FAIM3, FTHL8, TFPI, PPAPDC2, PHTF1, MITD1, PKIA, NAB1, FLJ42986, TMEM140, BMP6, IPO11, SLC39A8, IL4R, CBLB, LOC338799, TGIF1, FTH1, PLGLA, GABRB1, PLGLB1, ST6GALNAC2, MGC11082, QPCTL, NSD1, SLA, MGC16121, LOC132241, PDE8A, EID2, SMOX, KLHL21, FLJ11783, HIST1H3D, PRG1, HIST1H4H, ATP10D, LOC389791, CDKN2C, LOC648164, C7orf53, LOC339192, SLC36A4, NUAK2, LOC730256, NF1, INHA, LYPD3, SIPA1L1, HIST1H2BD, NCRNA00085, CXorf65, ZNF613, COL6A1, MEGF9, TTC9C, FTHL3, ECHDC3, LOC653853, PIM3, TNFAIP8L1, KCNE1L, SLC26A11, RCN1, VEGFA, PTCHD1, CRLF2, C19orf28, LOC100130828, HAS1, FMNL2, CHST2, SH3TC1, MGC42367, LOC285741, MEP1A, BACH2, GAB1, MGC61598, HIST1H3F, PLAUR, INDO, GPR160, SDHAF1, TNFAIP8L2, C5orf53, KCNJ8, ZNF521, PDGFC, ATP2B1, C8orf44, MAST3, LIMK2, LOC646043, KBTBD7, SLC25A15, AGPAT6, ZNF200, LOC727820, FPR1, GNLY, PTPRC, SPOCK1, RBKS, VNN3 |
| ILC2 | 76 | PTGER3, IL26, ERI1, CHCHD3, ERAP2, LOC387882, SH2D1A, CCR1, BASP1, MAPK6, C10orf61, ATP9A, ALOX5AP, CCL3L3, GZMA, TSPAN13, GPR55, DCBLD2, CD38, OSTF1, CTLA4, PELO, CREG1, IL4, TUBB6, MUC1, CCL3L1, QPRT, BAG2, RASGRP2, GZMB, PHLDA1, NFIL3, CXorf21, SYT11, HSH2D, TCF4, GPR56, ZEB2, BHLHE22, UBE2Z, PSMD1, LIMA1, MT1F, ENTPD1, ATP8B4, RNF19B, WARS, ASCL2, RHOBTB3, NPTX1, INPP1, MTE, PAGE5, RAB9A, GNA15, ICOS, RNASEN, FCER1G, MT1A, MYL6B, SRGN, EPSTI1, GBP5, MLLT11, PSMB8, PSEN2, TESC, ZBTB32, MIR21, TMEM49, H1F0, UQCRC2, PRDM1, TCTN3, MYB |
| HUVEC | 181 | SAMD4A, TNIP3, UGCG, PTPRK, APOL2, LOC387763, PANX1, TGFB2, PLAU, HAS3, TICAM1, SLC11A2, SERPINB8, MSC, SLC25A37, TNC, IRF1, FAM101A, LOC653778, KIAA1199, LOC646517, KIAA0247, TUBB2B, FAM129A, OPTN, HERPUD1, TGFB3, SQSTM1, GPR37L1, ADAMTS1, TRIM21, ATF3, GFPT2, CXCL2, THRAP4, NKX3-1, CBR3, CNKSR3, F2RL1, SLC2A6, OTUD4, CCL8, SSH1, C8orf4, TNFRSF11B, SBNO2, NKD2, TNFSF15, A4GALT, IFI30, MSX1, PDE5A, ADAMTS9, CX3CL1, ADORA2A, B4GALT1, ICAM1, C1orf24, C3orf52, FLJ31951, CHIC2, NRIP1, SHB, DNAJB9, ARID5B, ETS1, HINT3, ISG20, CXCL1, HRH1, JAG1, CCRN4L, PPAP2B, IKBKE, RASD1, CCL7, CXCL11, ST5, PDZD2, SLC41A2, PBEF1, STAT5A, SLC15A3, CAB39, PARP14, LITAF, LOC730417, RHBDF2, HEY1, WTAP, PRRX1, CXCR7, CXCL10, C6orf58, SERPINB2, S100A3, IER3, CDK6, LRIG3, MOBKL2C, CEBPD, WWC1, EHD1, EFNA1, SNFT, NFKBIE, SDC4, LAMC2, C6orf128, CXCL5, ITGAV, SAMD9L, CSF3, TMPIT, ADAMTS4, SLC25A28, DDX58, CFB, FSTL3, CXCL6, KYNU, UPP1, CCL20, CD47, STX11, IFNGR2, PAPLN, CITED4, CDC42EP2, GPRC5A, SOX17, CLIP2, CD83, SLC30A7, IL15, BMP2, RSPO3, F3, LOC654103, OBFC2A, ZNFX1, ICOSLG, CD69, NCOA7, BCL6, CTHRC1, DRAM, SLC31A2, UBD, SERPINA3, BIRC2, MMP10, FBXO32, COL27A1, RND1, PRIC285, APOL3, TIFA, MAFF, CCL2, OSGIN2, NNMT, DARC, IL6, GALNT4, GRAMD3, BTG3, SAV1, SAT1, YPEL2, PSTPIP2, AXUD1, NLF2, IFIT3, EDG1, MCTP1, LYPD6, DSCR1, SLC7A2, TNFAIP1, ESM1 |

**B** **Venn diagram tables of all down-regulated IL-33 stimulated gene signatures**

Numbers of genes inputted

| **List names** | **number of elements** | **number of unique elements** |
| --- | --- | --- |
| Basophil | 59 | 59 |
| HUVEC | 61 | 61 |
| ILC2 | 32 | 32 |
| Mast cell | 353 | 353 |
| **Overall number of unique elements** | | **491** |

Venn diagram table

| **Names** | **total** | **elements** |
| --- | --- | --- |
| Basophil Mast cell | 2 | HK3, MS4A3 |
| ILC2 Mast cell | 5 | MATK, ZBTB16, ATP1B1, SCN2A, TP53I11 |
| HUVEC Mast cell | 4 | GPR162, GFOD1, TXNIP, SMAD6 |
| Basophil HUVEC | 3 | SLC45A3, KLF2, E2F2 |
| Mast cell | 342 | EMP1, KIAA1614, B3GNT5, CREB3L1, COBLL1, SCN7A, DNM1P35, AURKB, RYR3, E2F8, GCSAM, TSPAN11, M1AP, IL16, SLFN14, CDSN, IQGAP3, CHST8, FMN1, SLC22A18AS, CA8, CCNA1, DEPTOR, AC108053.1, HPN, OTOA, OR8G1, C12orf42, CLDN9, MKI67, DNAH8, KLF2P4, RGS5, S100Z, NFE2, FAR2P4, MIR223HG, MRGPRX6P, LINC02115, TSPYL2, UBE2D3-AS1, AL139142.1, ADGRG4, INKA1, LILRB2, LINC01918, NDRG2, NHSL1, CDH23, NTM, TTN, AC004233.2, REXO5, SGSM1, AC020914.3, NLRC4, ARHGEF37, TMCC3, SIGLEC15, UNC80, SLA, LTB4R2, EEF1A1P14, CCDC188, SYNE2, KCNK3, SESN3, LURAP1L, PTPRN2-AS1, LINC01583, UNC79, SLC15A2, BTBD19, PDE2A, LINC02773, AL136456.1, OR10V3P, ASPM, FAM20A, PLXNA2, AC009652.1, BTG1P1, GATA3, LINC00607, PM20D1, SGK1, PPP2R2B, FCER1A, KRT72, CALB1, AP003119.1, KRT73, RNASE6, SYN3, ANXA8, AC009145.1, CSF3R, AC020907.5, IL12A-AS1, AK5, PITPNM3, RBM20, ITGA3, CDK15, SCPEP1, PTENP1-AS, AC080013.3, AC087369.2, TNNI2, HGD, SVOPL, VWA5B2, AC091563.1, LIPN, SLC22A18, NPTX1, CYRIA, MAF, SHANK1, CD226, TCTEX1D1, AL356804.1, MAS1LP1, CTSG, OR8G3P, CPNE5, ICAM4, SFTPD, ABHD4, ZMYND15, SEPTIN7P9, RPS17P1, FAR2P1, TBC1D2, CSF2RA, ANLN, LILRB3, AC112178.1, HBD, CD2, RTN4R, UTS2, AC018450.1, GJA3, KREMEN1, ARL15, ANK3, AL049552.2, UBE2C, SLC27A3, AC104248.1, TIMP2, ANXA8L1, ZMAT4, CACNG8, FOXN2, RHOQP3, NEURL1B, MYLK, RASGEF1B, ABLIM3, HS3ST1, AL021392.1, AP001122.1, S1PR1, DPY19L2P1, LINC02395, TNFRSF14-AS1, RAB3B, C1orf127, PICART1, CREG2, AC010210.1, SERPINB1, AL078590.2, STAB1, CHST15, CDK1, AC116424.1, ADGRE4P, CFAP58-DT, SLC37A2, REM1, LINC00323, LINC01529, JPH4, SORBS3, PANX3, FREM1, SDK2, CNIH3, SCIN, AL356489.3, TNS1, KCNJ5, LINC02147, TXLNB, Z85996.3, C3AR1, AC073869.3, LINC02082, PRKG2, SEMA6B, GPR34, HNF4A-AS1, ERVFRD-1, TNXB, AC104964.2, HACD4, BIRC7, IFI30, KY, PDZPH1P, KIF18B, FAM111B, CYP4F30P, SERPINB9P1, MAS1L, ADRA2A, CIDEB, CRYBB1, PLEKHA5, RGS4, AL136018.1, COL25A1, PVALB, NRGN, SIGLEC12, SCN1B, CALCRL, AC006230.1, F2R, ADRA1D, AC010175.1, ADAM11, ARAP3, MLPH, LINC02008, AL133313.1, APBB1IP, CXCL16, MRGPRX2, RHBDF1, SPINK4, ADGRG7, ADAMTSL3, AL356489.2, EGFEM1P, LBH, AC019197.1, CCDC30, SHC3, ZNF367, ARRDC4, PRR5L, SSPOP, PLXDC1, AC011899.2, MROH7, RGS13, FOXF1, ZNF467, LRRC7, AC018865.2, ESR2, LINC00987, ZNF483, C11orf45, AIF1, SLC22A10, MRGPRX7P, SIPA1L2, SGIP1, BX571818.1, AL590617.2, RALGPS2, F8, AC113414.1, IRAG1, EGR3, SNCG, FBP1, LINC01366, OR8A1, PDK4, AC092650.1, EVPL, AL021917.1, LINC02829, TREML2, TRBV26OR9-2, SNX29, SEMA3D, ZBTB20, EFHC2, ICAM5, PPIAP41, OR10Y1P, CPED1, RASGEF1C, SLC38A11, P2RY10, NAALADL2, MYO16, LOXL3, PEPD, VWC2, MPIG6B, PADI2, AL355365.1, LINC02656, MIR3681HG, ST14, TOX2, KRT17P8, SNX21, IL18BP, NABP1, AC060766.6, CR1, PTCH2, ARHGEF40, LTB4R, DHRS9, PDE7B, OR8G5, MAGEB2, RASL10A, LINCR-0001, ELFN1, AL670729.3, MS4A6A, DLGAP5, PLXNA4, FAM216B, ITPR3, TGM5, SLC38A6, MOB3B, KIAA0040, PIK3IP1, AL356489.1 |
| Basophil | 54 | PTGER3, ITGAM, SOCS2, FAM113B, XK, CCR2, MMD, SPRYD5, ALOX5, LTB, CCR1, KCNK17, LOC399940, SFXN1, OSBPL3, TCN1, C9orf140, FAM65B, C10orf128, OSM, TCP11L2, SELL, FLJ43093, BHLHB2, FOSB, FLNB, ST6GAL1, LAT2, LST1, HBEGF, NDFIP2, LAT, KIAA0564, CEACAM1, ROPN1L, DUSP5, CD69, LOC100129034, IFITM1, CCL3, JUN, GZMB, H3F3B, CISH, RGPD5, LOC732393, KRT81, SOCS1, STK17B, CORO6, KRT86, TIMM8A, CKAP4, PIM1 |
| ILC2 | 27 | IL7R, LEF1, AMICA1, FAM134B, ASTN2, LSP1, TCEAL3, KIT, FAM102A, SERPINE2, RXRA, LIME1, TNFRSF1A, SNORA5A, KRT1, PLCG1, SH3GLB2, ARRDC3, OSBPL7, SPOCK2, NR1D2, SLC5A3, CAST, MAZ, APP, ABLIM1, NEK6 |
| HUVEC | 54 | JAG2, STX3, NUAK1, CMTM8, PPFIBP2, GJA4, FRMD3, FAM124B, GPR126, AXIIR, RARA, TRAM2, KRT80, HOXA10, DKFZp761P0423, NDRG4, C8orf55, SDPR, HES1, DACH1, CLEC14A, SERTAD4, LOC400451, PPP1R3C, LMCD1, LRRC33, CARD10, DUSP4, TMEM46, SALL2, GIMAP7, RUNX1T1, CABLES1, VPS37D, BMP4, KCTD12, CDCA7, RGC32, GALNAC4S-6ST, PCDH7, PRICKLE1, CYP1A1, TSC22D3, ZNF395, KLHL3, LFNG, SOX18, LYL1, TMEM37, EFCAB4A, FAM84B, SORBS2, MAFB, C3orf54 |

**Supplementary Table S9** The 81 genes up-regulated in at least n=2 or more IL-33 signatures and its pathway enrichment analysis.

| **Genes** |  |  |  |  |
| --- | --- | --- | --- | --- |
| NFKB2, RELB, OLR1, MAP3K8, NFKBIZ, PTGS2, GBP4, CSF2, GBP2, FEZ1, THBS1, STAT4, BAMBI, TGFBR3, PCSK5, RASL11A, MUC20, IRF4, IL2RA, MT1E, CD74, ITGA1, IL5, TMEM64, PLA2G4A, SLC27A2, IL17RB, LRIG1, CCL3, TNFRSF8, IL13, PIM2, BATF, C15orf48, EBI3, IRAK2, SMAD3, TNFRSF9, PDLIM4, TRAF1, TNIP1, IL7R, MT1G, ZFPM2, ZC3H12A, IL1A, TNFAIP2, IL32, HLA-B, LTB, DSE, PLA2G4C, IL1B, CD200, IL4I1, ST6GAL1, NFKBIA, GCH1, IL15RA, SLC7A11, POU2F2, TNFAIP3, BIRC3, SOD2, ABCG1, TNFAIP6, IFIH1, NFKB1, MARCH3, DUSP16, NINJ1, IL18R1, RGS2, TNFSF10, GBP1, TAP1, MX1, IL8, ELL2, TRIB1, CSF2RB | | | | |
|  |  |  |  |  |
| **Pathway analysis Term** | **Overlap** | **P-value** | **Adjusted P-value** | **Genes** |
| Cytokine-cytokine receptor interaction | 18/295 | 1.29E-16 | 2.03E-14 | IL32, IL15RA, CSF2, TNFRSF9, IL13, EBI3, CSF2RB, IL17RB, IL1A, IL5, IL1B, IL2RA, TNFSF10, CCL3, TNFRSF8, LTB, IL7R, IL18R1 |
| NF-kappa B signaling pathway | 10/104 | 1.28E-11 | 1.01E-09 | NFKBIA, IL1B, TNFAIP3, TRAF1, LTB, PTGS2, NFKB1, BIRC3, RELB, NFKB2 |
| TNF signaling pathway | 10/112 | 2.71E-11 | 1.43E-09 | NFKBIA, CSF2, IL1B, TNFAIP3, MAP3K8, TRAF1, PTGS2, NFKB1, IL18R1, BIRC3 |
| IL-17 signaling pathway | 9/94 | 1.49E-10 | 5.90E-09 | NFKBIA, CSF2, IL5, IL1B, IL13, TNFAIP3, PTGS2, IL17RB, NFKB1 |
| Inflammatory bowel disease | 8/65 | 2.13E-10 | 6.72E-09 | IL1A, SMAD3, IL5, IL1B, IL13, STAT4, NFKB1, IL18R1 |
| Pathways in cancer | 15/531 | 3.06E-09 | 8.07E-08 | IL15RA, SMAD3, IL13, CSF2RB, TRAF1, PTGS2, NFKB1, NFKB2, NFKBIA, IL5, IL2RA, STAT4, PIM2, IL7R, BIRC3 |
| Measles | 8/139 | 9.33E-08 | 2.11E-06 | IFIH1, NFKBIA, IL1A, IL1B, IL2RA, MX1, TNFAIP3, NFKB1 |
| Hematopoietic cell lineage | 7/99 | 1.51E-07 | 2.99E-06 | IL1A, CSF2, IL5, IL1B, IL2RA, ITGA1, IL7R |
| Lipid and atherosclerosis | 9/215 | 2.16E-07 | 3.78E-06 | NFKBIA, IL1B, TNFSF10, CCL3, OLR1, POU2F2, SOD2, ABCG1, NFKB1 |
| Human T-cell leukemia virus 1 infection | 9/219 | 2.53E-07 | 3.78E-06 | NFKBIA, IL15RA, CSF2, SMAD3, IL2RA, HLA-B, NFKB1, RELB, NFKB2 |
| Necroptosis | 8/159 | 2.63E-07 | 3.78E-06 | IL1A, IL1B, PLA2G4C, TNFSF10, STAT4, TNFAIP3, PLA2G4A, BIRC3 |
| JAK-STAT signaling pathway | 8/162 | 3.03E-07 | 4.00E-06 | IL15RA, CSF2, IL5, IL2RA, IL13, STAT4, CSF2RB, IL7R |
| NOD-like receptor signaling pathway | 8/181 | 7.06E-07 | 8.58E-06 | NFKBIA, IL1B, TNFAIP3, GBP2, GBP1, GBP4, NFKB1, BIRC3 |
| Th1 and Th2 cell differentiation | 6/92 | 1.98E-06 | 2.23E-05 | NFKBIA, IL5, IL2RA, IL13, STAT4, NFKB1 |
| MAPK signaling pathway | 9/294 | 2.93E-06 | 3.09E-05 | IL1A, IL1B, PLA2G4C, PLA2G4A, MAP3K8, DUSP16, NFKB1, RELB, NFKB2 |
| C-type lectin receptor signaling pathway | 6/104 | 4.04E-06 | 3.99E-05 | NFKBIA, IL1B, PTGS2, NFKB1, RELB, NFKB2 |
| Th17 cell differentiation | 6/107 | 4.77E-06 | 4.43E-05 | NFKBIA, SMAD3, IRF4, IL1B, IL2RA, NFKB1 |
| Influenza A | 7/172 | 6.27E-06 | 5.50E-05 | IFIH1, NFKBIA, IL1A, IL1B, MX1, TNFSF10, NFKB1 |
| Fc epsilon RI signaling pathway | 5/68 | 8.21E-06 | 6.82E-05 | CSF2, IL5, IL13, PLA2G4C, PLA2G4A |
| Osteoclast differentiation | 6/127 | 1.28E-05 | 1.01E-04 | NFKBIA, IL1A, IL1B, NFKB1, RELB, NFKB2 |
| Leishmaniasis | 5/77 | 1.51E-05 | 1.14E-04 | NFKBIA, IL1A, IL1B, PTGS2, NFKB1 |
| Epstein-Barr virus infection | 7/202 | 1.79E-05 | 1.28E-04 | NFKBIA, HLA-B, TNFAIP3, TAP1, NFKB1, RELB, NFKB2 |
| Apoptosis | 6/142 | 2.41E-05 | 1.66E-04 | NFKBIA, TNFSF10, CSF2RB, TRAF1, NFKB1, BIRC3 |
| Human cytomegalovirus infection | 7/225 | 3.56E-05 | 2.27E-04 | NFKBIA, IL1B, HLA-B, CCL3, TAP1, PTGS2, NFKB1 |
| Small cell lung cancer | 5/92 | 3.59E-05 | 2.27E-04 | NFKBIA, TRAF1, PTGS2, NFKB1, BIRC3 |
| Rheumatoid arthritis | 5/93 | 3.78E-05 | 2.30E-04 | IL1A, CSF2, IL1B, CCL3, LTB |
| T cell receptor signaling pathway | 5/104 | 6.46E-05 | 3.65E-04 | NFKBIA, CSF2, IL5, MAP3K8, NFKB1 |
| Toll-like receptor signaling pathway | 5/104 | 6.46E-05 | 3.65E-04 | NFKBIA, IL1B, CCL3, MAP3K8, NFKB1 |
| Legionellosis | 4/57 | 8.38E-05 | 4.56E-04 | NFKBIA, IL1B, NFKB1, NFKB2 |
| Herpes simplex virus 1 infection | 9/498 | 1.82E-04 | 9.59E-04 | IFIH1, NFKBIA, CD74, IL1B, HLA-B, TAP1, POU2F2, NFKB1, BIRC3 |
| Coronavirus disease | 6/232 | 3.59E-04 | 1.83E-03 | IFIH1, NFKBIA, CSF2, IL1B, MX1, NFKB1 |
| Hepatitis B | 5/162 | 5.11E-04 | 2.52E-03 | IFIH1, NFKBIA, SMAD3, STAT4, NFKB1 |
| Graft-versus-host disease | 3/42 | 6.56E-04 | 3.14E-03 | IL1A, IL1B, HLA-B |
| Type I diabetes mellitus | 3/43 | 7.03E-04 | 3.20E-03 | IL1A, IL1B, HLA-B |
| AGE-RAGE signaling pathway in diabetic complications | 4/100 | 7.29E-04 | 3.20E-03 | IL1A, SMAD3, IL1B, NFKB1 |
| Viral protein interaction with cytokine and cytokine receptor | 4/100 | 7.29E-04 | 3.20E-03 | IL2RA, TNFSF10, CCL3, IL18R1 |
| Chagas disease | 4/102 | 7.85E-04 | 3.35E-03 | NFKBIA, IL1B, CCL3, NFKB1 |
| Tuberculosis | 5/180 | 8.24E-04 | 3.42E-03 | IL1A, CD74, IRAK2, IL1B, NFKB1 |
| Transcriptional misregulation in cancer | 5/192 | 1.10E-03 | 4.44E-03 | CSF2, NFKBIZ, TRAF1, NFKB1, BIRC3 |
| Kaposi sarcoma-associated herpesvirus infection | 5/193 | 1.12E-03 | 4.44E-03 | NFKBIA, CSF2, HLA-B, PTGS2, NFKB1 |
| Ovarian steroidogenesis | 3/51 | 1.16E-03 | 4.46E-03 | PLA2G4C, PLA2G4A, PTGS2 |
| Viral carcinogenesis | 5/203 | 1.41E-03 | 5.29E-03 | NFKBIA, HLA-B, TRAF1, NFKB1, NFKB2 |
| VEGF signaling pathway | 3/59 | 1.77E-03 | 6.49E-03 | PLA2G4C, PLA2G4A, PTGS2 |
| Arachidonic acid metabolism | 3/61 | 1.94E-03 | 6.96E-03 | PLA2G4C, PLA2G4A, PTGS2 |
| FoxO signaling pathway | 4/131 | 1.98E-03 | 6.96E-03 | SMAD3, TNFSF10, SOD2, IL7R |
| Cytosolic DNA-sensing pathway | 3/63 | 2.13E-03 | 7.33E-03 | NFKBIA, IL1B, NFKB1 |
| Human papillomavirus infection | 6/331 | 2.25E-03 | 7.56E-03 | ITGA1, MX1, HLA-B, PTGS2, THBS1, NFKB1 |
| Acute myeloid leukemia | 3/67 | 2.54E-03 | 8.37E-03 | CSF2, PIM2, NFKB1 |
| RIG-I-like receptor signaling pathway | 3/70 | 2.88E-03 | 9.29E-03 | IFIH1, NFKBIA, NFKB1 |
| Shigellosis | 5/246 | 3.24E-03 | 0.01 | NFKBIA, CSF2, TNIP1, IL1B, NFKB1 |
| Phagosome | 4/152 | 3.39E-03 | 0.01 | HLA-B, OLR1, TAP1, THBS1 |
| Salmonella infection | 5/249 | 3.41E-03 | 0.01 | NFKBIA, IL1B, TNFSF10, NFKB1, BIRC3 |
| Oxytocin signaling pathway | 4/154 | 3.55E-03 | 0.01 | RGS2, PLA2G4C, PLA2G4A, PTGS2 |
| Chronic myeloid leukemia | 3/76 | 3.64E-03 | 0.01 | NFKBIA, SMAD3, NFKB1 |
| Pertussis | 3/76 | 3.64E-03 | 0.01 | IL1A, IL1B, NFKB1 |
| Cellular senescence | 4/156 | 3.72E-03 | 0.01 | IL1A, SMAD3, HLA-B, NFKB1 |
| Antigen processing and presentation | 3/78 | 3.91E-03 | 0.01 | CD74, HLA-B, TAP1 |
| alpha-Linolenic acid metabolism | 2/25 | 4.58E-03 | 0.01 | PLA2G4C, PLA2G4A |
| PD-L1 expression and PD-1 checkpoint pathway in cancer | 3/89 | 5.66E-03 | 0.02 | NFKBIA, NFKB1, BATF |
| Linoleic acid metabolism | 2/29 | 6.13E-03 | 0.02 | PLA2G4C, PLA2G4A |
| TGF-beta signaling pathway | 3/94 | 6.58E-03 | 0.02 | SMAD3, BAMBI, THBS1 |
| Asthma | 2/31 | 6.98E-03 | 0.02 | IL5, IL13 |
| Inflammatory mediator regulation of TRP channels | 3/98 | 7.38E-03 | 0.02 | IL1B, PLA2G4C, PLA2G4A |
| Amoebiasis | 3/102 | 8.24E-03 | 0.02 | CSF2, IL1B, NFKB1 |
| Pathogenic Escherichia coli infection | 4/197 | 8.42E-03 | 0.02 | NFKBIA, IL1B, TNFSF10, NFKB1 |
| Insulin resistance | 3/108 | 9.63E-03 | 0.02 | NFKBIA, SLC27A2, NFKB1 |
| Allograft rejection | 2/38 | 0.01 | 0.02 | IL5, HLA-B |
| Primary immunodeficiency | 2/38 | 0.01 | 0.02 | TAP1, IL7R |
| Toxoplasmosis | 3/112 | 0.01 | 0.02 | NFKBIA, NFKB1, BIRC3 |
| Human immunodeficiency virus 1 infection | 4/212 | 0.01 | 0.02 | NFKBIA, HLA-B, TAP1, NFKB1 |
| Serotonergic synapse | 3/113 | 0.01 | 0.02 | PLA2G4C, PLA2G4A, PTGS2 |
| Neurotrophin signaling pathway | 3/119 | 0.01 | 0.03 | NFKBIA, IRAK2, NFKB1 |
| ABC transporters | 2/45 | 0.01 | 0.03 | TAP1, ABCG1 |
| PI3K-Akt signaling pathway | 5/354 | 0.01 | 0.03 | IL2RA, ITGA1, IL7R, THBS1, NFKB1 |
| Natural killer cell mediated cytotoxicity | 3/131 | 0.02 | 0.03 | CSF2, TNFSF10, HLA-B |
| Intestinal immune network for IgA production | 2/48 | 0.02 | 0.03 | IL15RA, IL5 |
| Ether lipid metabolism | 2/49 | 0.02 | 0.03 | PLA2G4C, PLA2G4A |
| Malaria | 2/50 | 0.02 | 0.04 | IL1B, THBS1 |
| Yersinia infection | 3/137 | 0.02 | 0.04 | NFKBIA, IL1B, NFKB1 |
| Fluid shear stress and atherosclerosis | 3/139 | 0.02 | 0.04 | IL1A, IL1B, NFKB1 |
| Autoimmune thyroid disease | 2/53 | 0.02 | 0.04 | IL5, HLA-B |
| Phenylalanine, tyrosine and tryptophan biosynthesis | 1/6 | 0.02 | 0.05 | IL4I1 |
| Long-term depression | 2/60 | 0.02 | 0.05 | PLA2G4C, PLA2G4A |
| Mineral absorption | 2/60 | 0.02 | 0.05 | MT1G, MT1E |
| Non-alcoholic fatty liver disease | 3/155 | 0.03 | 0.05 | IL1A, IL1B, NFKB1 |
| Hepatitis C | 3/157 | 0.03 | 0.05 | NFKBIA, MX1, NFKB1 |
| Adipocytokine signaling pathway | 2/69 | 0.03 | 0.06 | NFKBIA, NFKB1 |
| Epithelial cell signaling in Helicobacter pylori infection | 2/70 | 0.03 | 0.06 | NFKBIA, NFKB1 |
| PPAR signaling pathway | 2/74 | 0.04 | 0.06 | OLR1, SLC27A2 |
| MicroRNAs in cancer | 4/310 | 0.04 | 0.07 | ZFPM2, PTGS2, THBS1, NFKB1 |
| Pancreatic cancer | 2/76 | 0.04 | 0.07 | SMAD3, NFKB1 |
| B cell receptor signaling pathway | 2/81 | 0.04 | 0.07 | NFKBIA, NFKB1 |
| Chemokine signaling pathway | 3/192 | 0.04 | 0.07 | NFKBIA, CCL3, NFKB1 |
| Peroxisome | 2/82 | 0.04 | 0.07 | SOD2, SLC27A2 |

**Supplementary Table S10** The 14 genes down-regulated in at least n=2 or more IL-33 signatures and its pathway enrichment analysis.

| **Genes** |  |  |  |  |
| --- | --- | --- | --- | --- |
| HK3, MS4A3, MATK, ZBTB16, ATP1B1, SCN2A, TP53I11, GPR162, GFOD1, TXNIP, SMAD6, SLC45A3, KLF2, E2F2 | | | | |
|  |  |  |  |  |
| **Pathway analysis Term** | **Overlap** | **P-value** | **Adjusted P-value** | **Genes** |
| Carbohydrate digestion and absorption | 2/47 | 4.83E-04 | 0.03 | HK3, ATP1B1 |
| Neomycin, kanamycin and gentamicin biosynthesis | 1/5 | 3.50E-03 | 0.10 | HK3 |
| Transcriptional misregulation in cancer | 2/192 | 0.01 | 0.12 | SLC45A3, ZBTB16 |
| Proximal tubule bicarbonate reclamation | 1/23 | 0.02 | 0.12 | ATP1B1 |
| MicroRNAs in cancer | 2/310 | 0.02 | 0.12 | SLC45A3, E2F2 |
| Galactose metabolism | 1/31 | 0.02 | 0.12 | HK3 |
| Fructose and mannose metabolism | 1/33 | 0.02 | 0.12 | HK3 |
| Starch and sucrose metabolism | 1/36 | 0.02 | 0.12 | HK3 |
| Aldosterone-regulated sodium reabsorption | 1/37 | 0.03 | 0.12 | ATP1B1 |
| Bladder cancer | 1/41 | 0.03 | 0.12 | E2F2 |
| Type II diabetes mellitus | 1/46 | 0.03 | 0.12 | HK3 |
| Amino sugar and nucleotide sugar metabolism | 1/48 | 0.03 | 0.12 | HK3 |
| Endocrine and other factor-regulated calcium reabsorption | 1/53 | 0.04 | 0.12 | ATP1B1 |
| Mineral absorption | 1/60 | 0.04 | 0.12 | ATP1B1 |
| Acute myeloid leukemia | 1/67 | 0.05 | 0.12 | ZBTB16 |
| Glycolysis / Gluconeogenesis | 1/67 | 0.05 | 0.12 | HK3 |
| Central carbon metabolism in cancer | 1/70 | 0.05 | 0.12 | HK3 |
| Melanoma | 1/72 | 0.05 | 0.12 | E2F2 |
| Non-small cell lung cancer | 1/72 | 0.05 | 0.12 | E2F2 |
| Thyroid hormone synthesis | 1/75 | 0.05 | 0.12 | ATP1B1 |
| Glioma | 1/75 | 0.05 | 0.12 | E2F2 |
| Pathways in cancer | 2/531 | 0.05 | 0.12 | ZBTB16, E2F2 |
| Chronic myeloid leukemia | 1/76 | 0.05 | 0.12 | E2F2 |
| Pancreatic cancer | 1/76 | 0.05 | 0.12 | E2F2 |
| Gastric acid secretion | 1/76 | 0.05 | 0.12 | ATP1B1 |

**Supplementary Table S11** Correlations between clinical data and ES of the mast cell IL-33-up-regulated gene signature.

| **Clinical feature** | **Correlation** | **P-value** | **Adj p value** |
| --- | --- | --- | --- |
| Epithelium CD3 T cells (cells/mm^2^) | 0.53 | 9.01E-03 | 0.95 |
| Sputum neutrophils (%) | 0.47 | 4.11E-07 | 4.32E-05 |
| Sputum neutrophils (x10^3^ cells/µL) | 0.45 | 1.57E-06 | 1.65E-04 |
| 11-dehydro-TXB2 (pg/mL) sputum eicosanoid | 0.37 | 1.84E-04 | 0.02 |
| IL6 (pg/ml) plasma biomarker (MSD assay) | 0.27 | 6.62E-03 | 0.70 |
| Galectin-3 (pg/ml) serum biomarker (Luminex) | 0.24 | 0.02 | 1.74 |
| Age | 0.24 | 0.02 | 1.71 |
| IL17AA (pg/ml) serum biomarker (Singulex) | 0.22 | 0.04 | 4.29 |
| PGD2 (pg/mL) sputum eicosanoid | 0.22 | 0.03 | 2.73 |
| 11-dehydroTXB2 (ng/mmolC) urine eicosanoid | 0.22 | 0.02 | 2.49 |
| Blood monocytes (x10^3^ cells/µL) | 0.22 | 0.03 | 3.00 |
| Blood neutrophil (x10^3^ cells/µL) | 0.21 | 0.03 | 3.60 |
| PGE2 (pg/ml) sputum eicosanoid | 0.20 | 0.04 | 4.42 |
| DPPIV (pg/ml) serum biomarker (Luminex) | -0.28 | 4.18E-03 | 0.44 |
| FVC (%) pre-salbutamol | -0.30 | 2.17E-03 | 0.23 |
| FEV1/FVC actual ratio | -0.38 | 6.08E-05 | 6.39E-03 |
| FEV1 (%) pre-salbutamol | -0.40 | 9.01E-03 | 0.95 |
| Sputum macrophages (x10^3^ cells/µL) | -0.66 | 4.11E-07 | 4.32E-05 |
| Sputum macrophages (%) | -0.67 | 1.57E-06 | 1.65E-04 |

**Supplementary Table S12** Correlations between asthma subject clinical data and ES of the basophil IL-33-up-regulated gene signature.

| **Clinical feature** | **Correlation** | **P-value** | **Adj p value** |
| --- | --- | --- | --- |
| Sputum neutrophils (%) | 0.65 | 1.04E-13 | 1.09E-11 |
| Sputum neutrophils (x10^3^ cells/µL) | 0.63 | 4.84E-13 | 5.09E-11 |
| Epithelium CD3 T cells (cells/mm^2^) | 0.49 | 0.02 | 1.98 |
| 11-dehydro-TXB2 (pg/mL) sputum eicosanoid | 0.31 | 1.43E-03 | 0.15 |
| 2,3-Dinor-TXB2 (ng/mmolC) urine eicosanoid | 0.26 | 7.70E-03 | 0.81 |
| hCRP (mg/L) biomarker | 0.23 | 0.02 | 2.41 |
| ACQ 1-5 average | 0.22 | 0.03 | 2.75 |
| No. of severe exacerbations per year | 0.21 | 0.04 | 3.85 |
| No. of exacerbations per year | 0.20 | 0.04 | 4.21 |
| Sputum eosinophils (x10^3^ cells/µL) | -0.20 | 0.05 | 4.81 |
| Sputum eosinophils (%) | -0.20 | 0.04 | 4.66 |
| FeNO | -0.21 | 0.03 | 3.46 |
| FEV1 (%) pre-salbutamol | -0.24 | 0.02 | 1.68 |
| FVC (%) pre-salbutamol | -0.27 | 6.30E-03 | 0.66 |
| AQLQ average | -0.28 | 5.05E-03 | 0.53 |
| Sputum macrophages (x10^3^ cells/µL) | -0.57 | 3.49E-10 | 3.67E-08 |
| Sputum macrophages (%) | -0.57 | 2.81E-10 | 2.95E-08 |

**Supplementary Table S13** Correlations between clinical data and ES of the ILC2 cell IL-33-up-regulated gene signature.

| **Clinical feature** | **Correlation** | **P-value** | **Adj p value** |
| --- | --- | --- | --- |
| Epithelium CD3 T cells (cells/mm^2^) | 0.57 | 4.31E-03 | 0.45 |
| Epithelium CD8 T cells (cells/mm^2^) | 0.50 | 0.01 | 1.57 |
| Sputum neutrophils (x10^3^ cells/µL) | 0.50 | 6.91E-08 | 7.2578E-06 |
| Sputum neutrophils (%) | 0.49 | 1.01E-07 | 1.0588E-05 |
| Submucosa CD4 T cells (cells/mm^2^) | 0.44 | 0.03 | 3.66 |
| IL17AA (pg/ml) serum biomarker (Singulex) | 0.22 | 0.05 | 5.15 |
| 11-dehydro-TXB2 (pg/mL) sputum eicosanoid | 0.20 | 0.04 | 4.60 |
| AQLQ average | -0.21 | 3.04E-02 | 3.20 |
| Sputum macrophages (%) | -0.30 | 2.13E-03 | 0.22 |
| Sputum macrophages (x10^3^ cells/µL) | -0.31 | 1.62E-03 | 0.17 |
| Sputum eosinophils (x10^3^ cells/µL) | -0.33 | 5.53E-04 | 0.06 |
| Sputum eosinophils (%) | -0.34 | 4.95E-04 | 0.05 |

**Supplementary Table S14** Correlations between clinical data and ES of the HUVEC IL-33-up-regulated gene signature.

| **Clinical feature** | **Correlation** | **P-value** | **Adj p value** |
| --- | --- | --- | --- |
| Sputum neutrophils (%) | 0.67 | 5.55E-15 | 5.82E-13 |
| Sputum neutrophils (x10^3^ cells/µL) | 0.66 | 1.86E-14 | 1.95E-12 |
| Epithelium CD3 T cells (cells/mm^2^) | 0.57 | 4.38E-03 | 0.46 |
| Submucosa CD4 T cells (cells/mm^2^) | 0.49 | 0.02 | 1.78 |
| Epithelium CD8 T cells (cells/mm^2^) | 0.43 | 0.04 | 4.05 |
| Submucosa CD3 T cells (cells/mm^2^) | 0.43 | 0.04 | 4.26 |
| 11-dehydro-TXB2 (pg/mL) sputum eicosanoid | 0.37 | 1.65E-04 | 0.02 |
| IL17AA (pg/ml) serum biomarker (Singulex) | 0.27 | 0.01 | 1.35 |
| IL-18 (pg/ml) serum biomarker (Singulex) | 0.19 | 0.05 | 5.20 |
| Galectin-3 (pg/ml) serum biomarker (Luminex) | 0.19 | 0.05 | 5.21 |
| FEV1 (%) pre-salbutamol | -0.21 | 0.03 | 3.34 |
| 12-HETE S-R (pg/mL) sputum eicosanoid | -0.24 | 0.02 | 1.65 |
| FVC (%) pre-salbutamol | -0.24 | 0.01 | 1.41 |
| AQLQ average | -0.27 | 6.48E-03 | 0.68 |
| Sputum eosinophils (x10^3^ cells/µL) | -0.38 | 8.06E-05 | 8.46E-03 |
| Sputum eosinophils (%) | -0.38 | 8.06E-05 | 8.46E-03 |
| Sputum macrophages (%) | -0.46 | 1.11E-06 | 1.16E-04 |
| Sputum macrophages (x10^3^ cells/µL) | -0.46 | 6.92E-07 | 7.26E-05 |

**Supplementary Table S15** Correlations between blood IL1RAP mRNA expression and blood cell counts.

| **Blood cell variable** | **Correlation** | **P value** | **Adjusted p value** | **Significance** |
| --- | --- | --- | --- | --- |
| Eosinophil | -0.1135075 | 0.01193 | 0.05965 | NS |
| Neutrophil | 0.4142479 | 2.20E-16 | 1.1E-15 | **** |
| Lymphocyte | -0.2974324 | 1.82E-11 | 9.1E-11 | **** |
| Monocyte | -0.1239027 | 0.006027 | 0.030135 | * |
| Basophil | -0.0215597 | 0.634 | 3.17 | NS |

**Supplementary Figures**

**
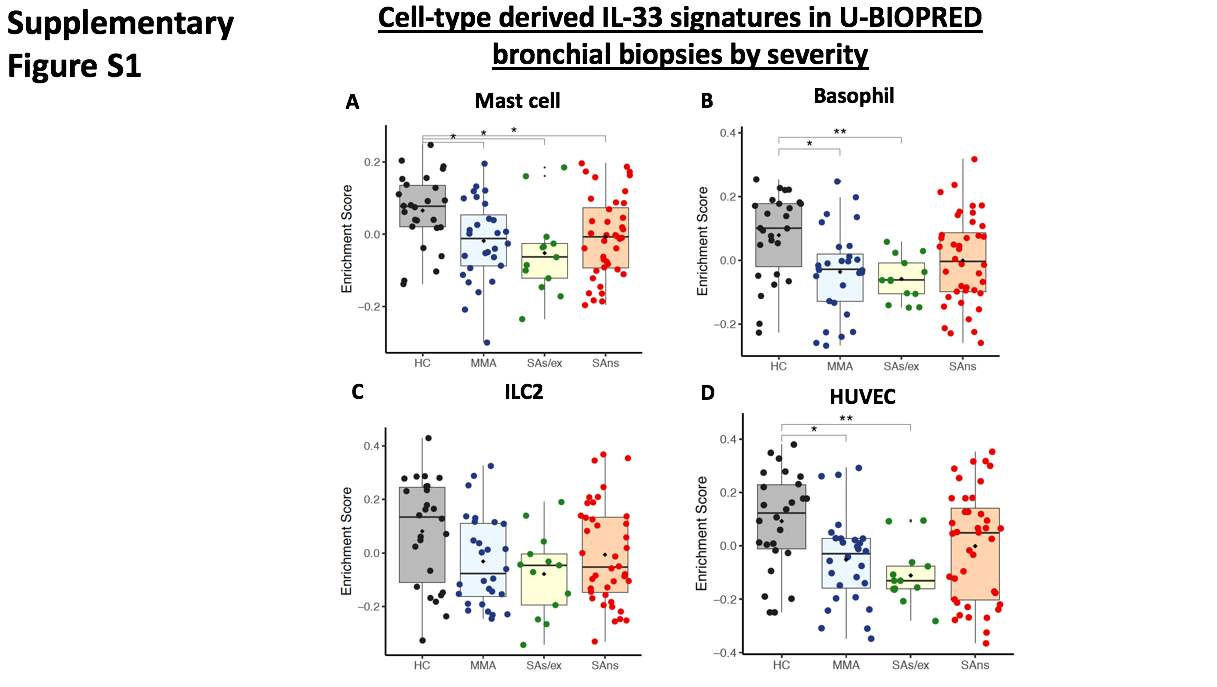
**

**Supplementary Figure S1.** Gene Set Variation Analysis (GSVA) boxplots of U-BIOPRED bronchial biopsy asthma enrichment scores for the (A) mast cell, (B) basophil, (C) ILC2 and (D) HUVEC cell derived IL-33 up-regulated gene signatures by severity cohort (HC=healthy control; MMA=mild-moderate asthma; SAs/ex =severe asthma smoking/ex-smoking; SAns =severe asthma non-smoking).

**
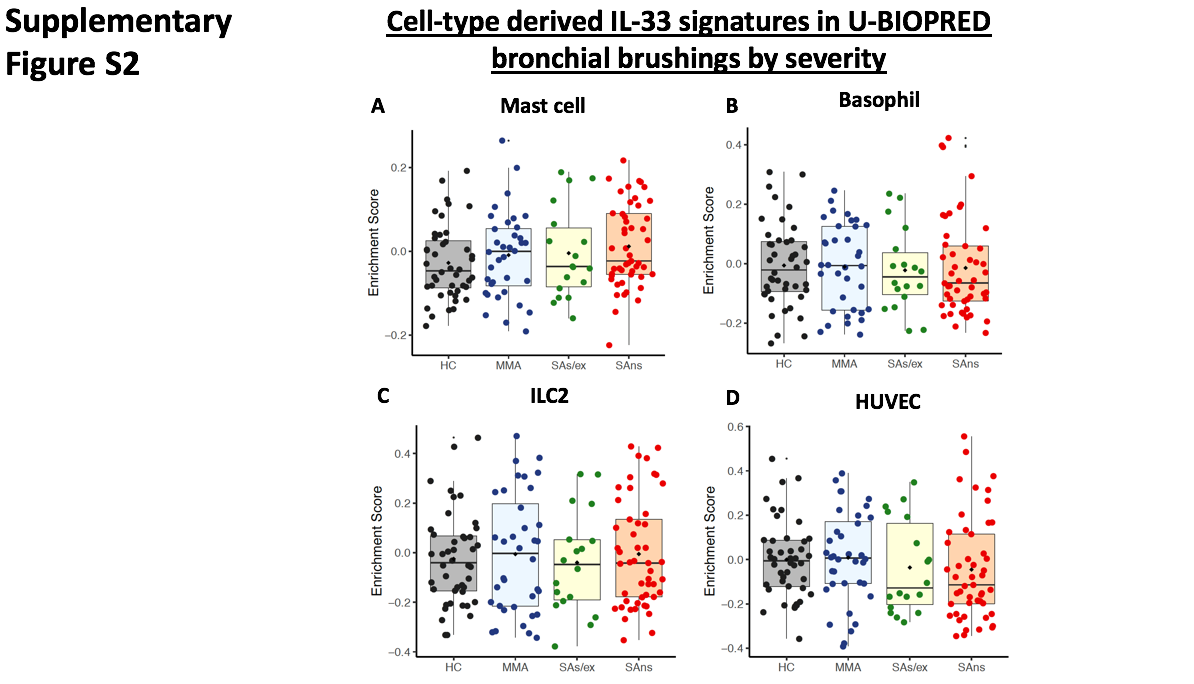
**

**Supplementary Figure S2.** Gene Set Variation Analysis (GSVA) boxplots of U-BIOPRED bronchial brushing asthma enrichment scores for the (A) mast cell, (B) basophil, (C) ILC2 and (D) HUVEC cell derived IL-33 up-regulated gene signatures by severity cohort (HC=healthy control; MMA=mild-moderate asthma; SAs/ex =severe asthma smoking/ex-smoking; SAns =severe asthma non-smoking).

**
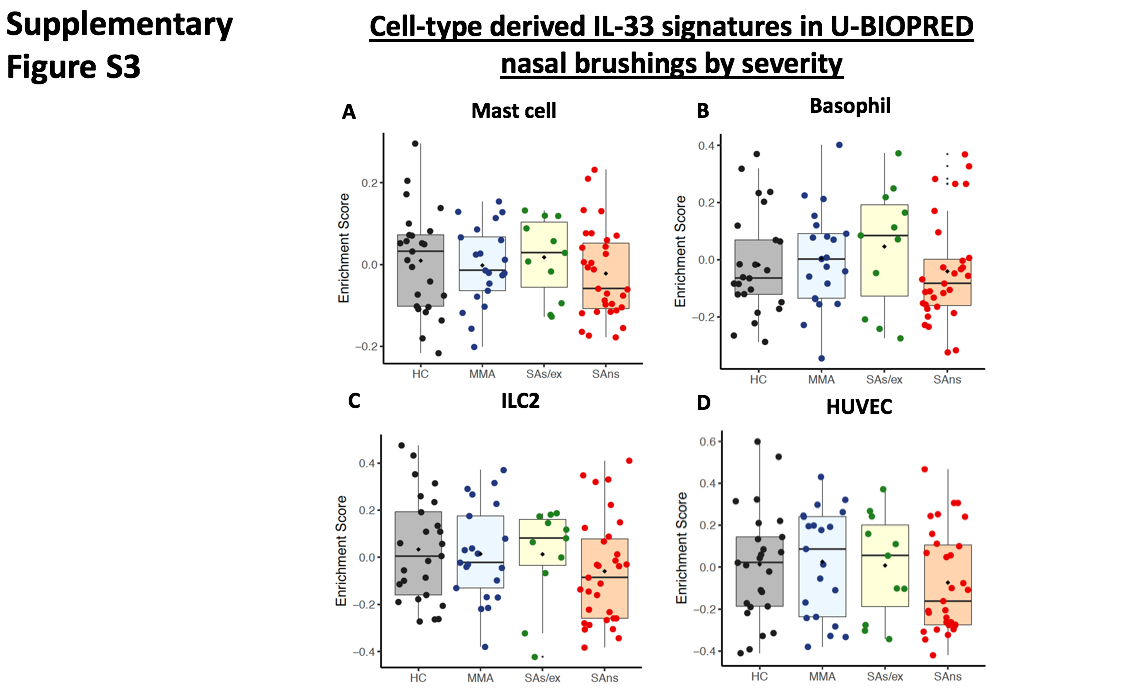
**

**Supplementary Figure S3.** Gene Set Variation Analysis (GSVA) boxplots of U-BIOPRED nasal brushing asthma enrichment scores for the (A) mast cell, (B) basophil, (C) ILC2 and (D) HUVEC cell derived IL-33 up-regulated gene signatures by severity cohort (HC=healthy control; MMA=mild-moderate asthma; SAs/ex =severe asthma smoking/ex-smoking; SAns =severe asthma non-smoking).

**
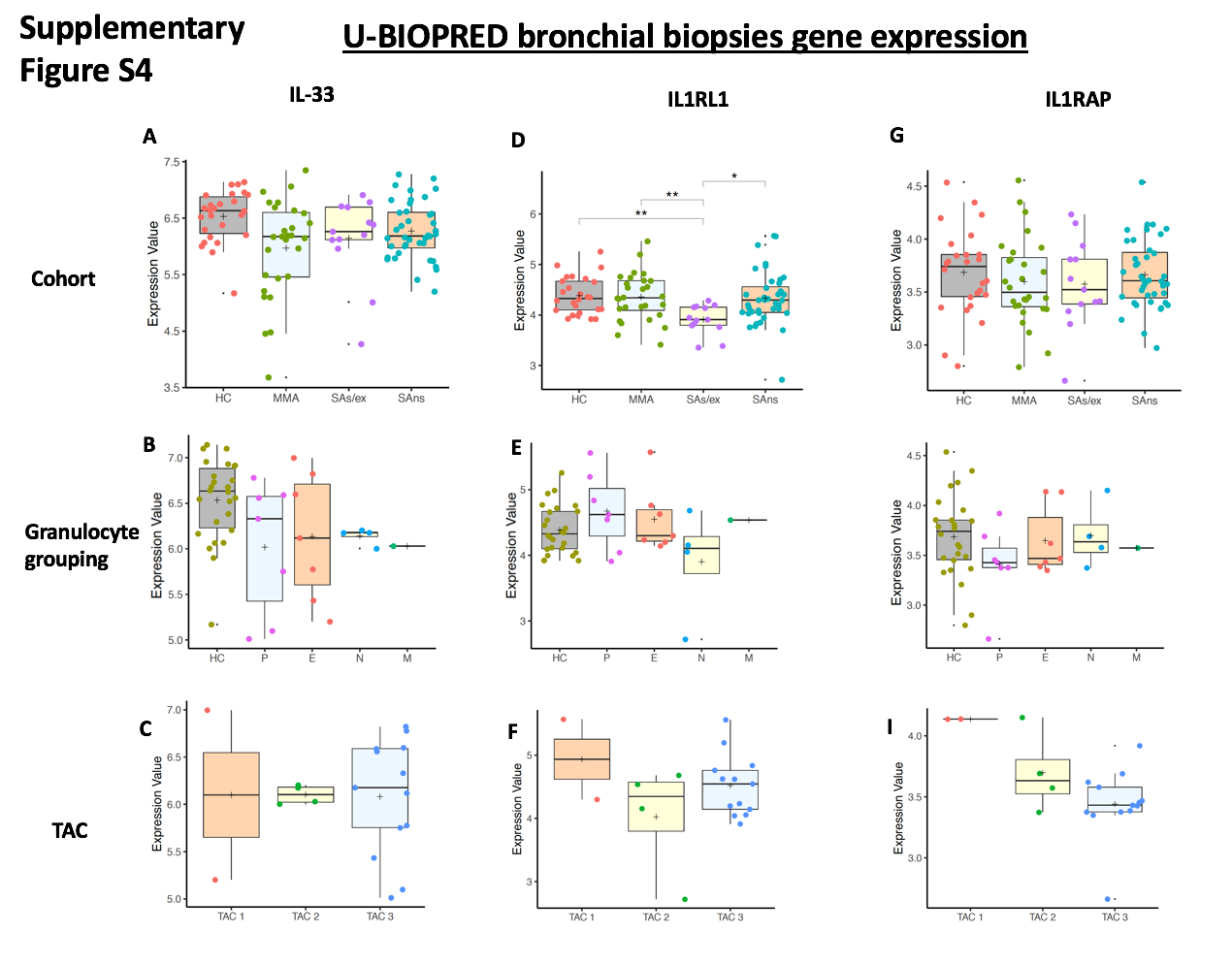
**

**Supplementary Figure S4.** Boxplots of U-BIOPRED bronchial biopsy asthma gene expression for IL-33, IL1RL1 (ST2) and IL1RAP (co-dimer of the IL-33 receptor) by severity cohort (HC=healthy control; MMA=mild-moderate asthma; SAs/ex =severe asthma smoking/ex-smoking; SAns =severe asthma non-smoking ) (A, D, G), granulocytic subtype (HC=healthy control, P=paucigranulocytic, E=eosinophilic, N=neutrophilic, M=mixed) (B, E, H), and transcriptome associated cluster (C, F, I) which reflects eosinophilic and non-eosinophilic phenotypes of asthma (TAC1=eosinophilic asthma, TAC2=neutrophilic asthma, TAC3=mixed or paucigranulocytic asthma).

**
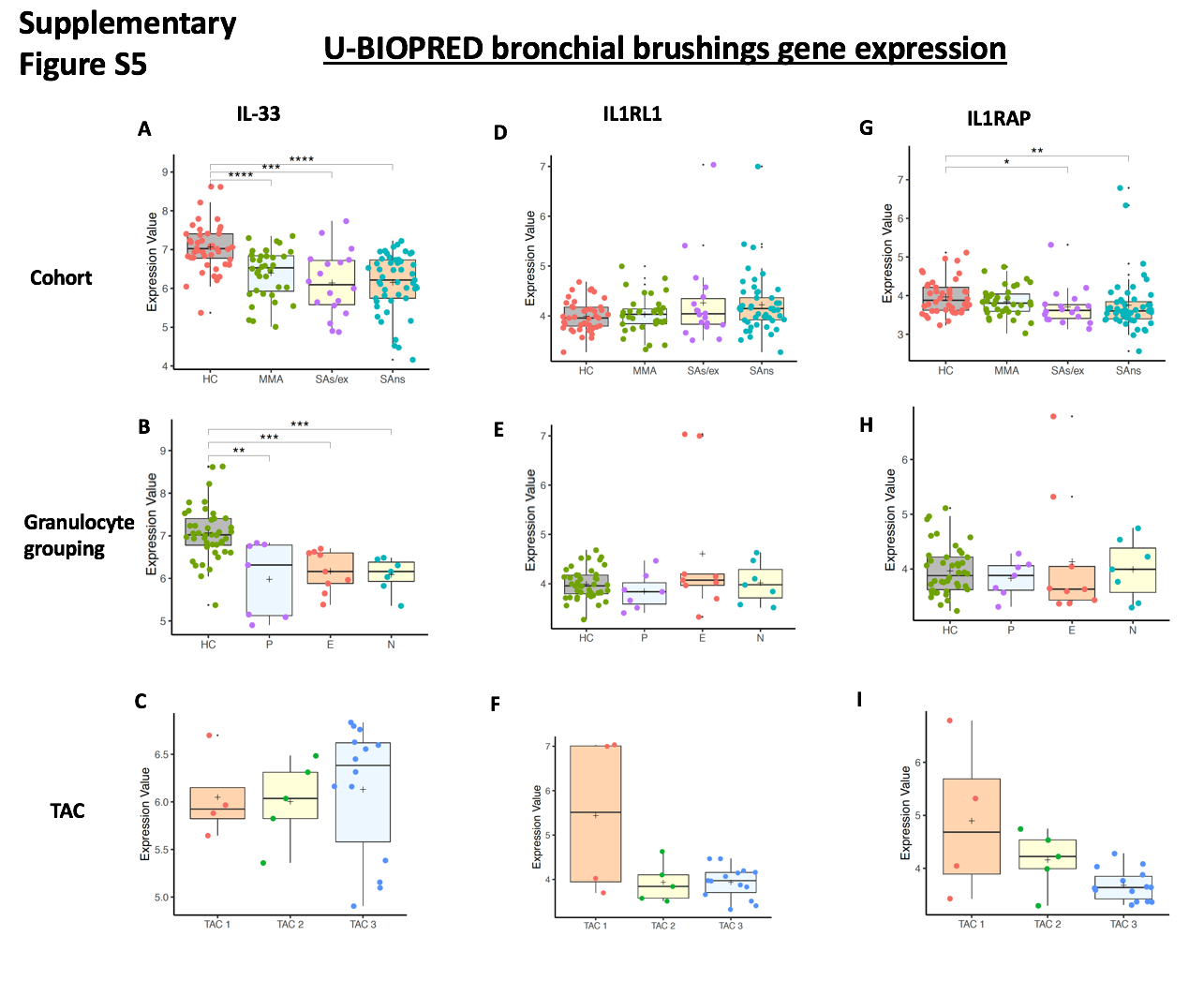
**

**Supplementary Figure S5.** Boxplots of U-BIOPRED bronchial brushing asthma gene expression for IL-33, IL1RL1 (ST2) and IL1RAP (co-dimer of the IL-33 receptor) by severity cohort (HC=healthy control; MMA=mild-moderate asthma; SAs/ex =severe asthma smoking/ex-smoking; SAns =severe asthma non-smoking ) (A, D, G), granulocytic subtype (HC=healthy control, P=paucigranulocytic, E=eosinophilic, N=neutrophilic, M=mixed) (B, E, H), and transcriptome associated cluster (C, F, I) which reflects eosinophilic and non-eosinophilic phenotypes of asthma (TAC1=eosinophilic asthma, TAC2=neutrophilic asthma, TAC3=mixed or paucigranulocytic asthma).

**
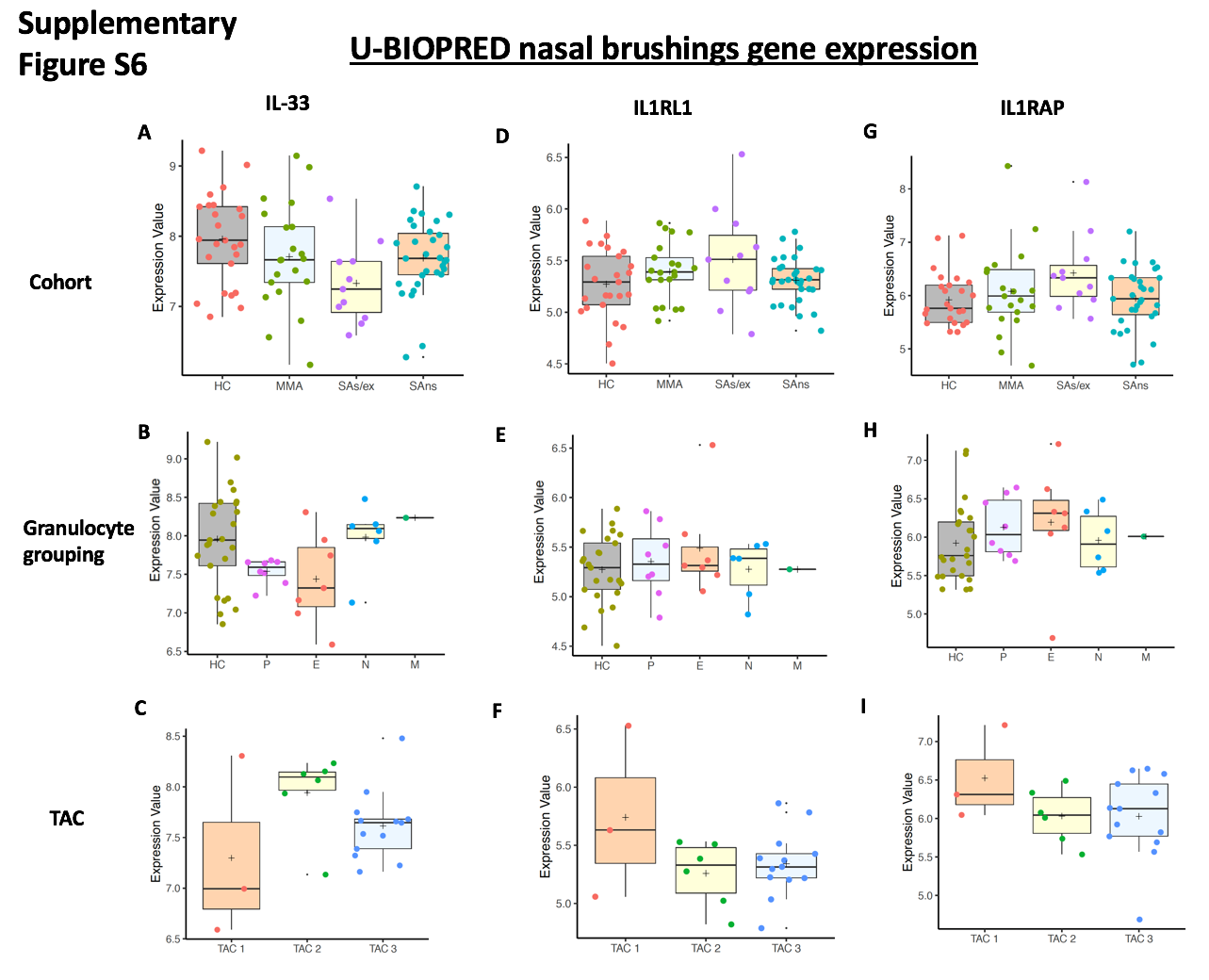
**

**Supplementary Figure S6.** Boxplots of U-BIOPRED nasal brushing asthma gene expression for IL-33, IL1RL1 (ST2) and IL1RAP (co-dimer of the IL-33 receptor) by severity cohort (HC=healthy control; MMA=mild-moderate asthma; SAs/ex =severe asthma smoking/ex-smoking; SAns =severe asthma non-smoking) (A, D, G), granulocytic subtype (HC=healthy control, P=paucigranulocytic, E=eosinophilic, N=neutrophilic, M=mixed) (B, E, H), and transcriptome associated cluster (C, F, I) which reflects eosinophilic and non-eosinophilic phenotypes of asthma (TAC1=eosinophilic asthma, TAC2=neutrophilic asthma, TAC3=mixed or paucigranulocytic asthma).


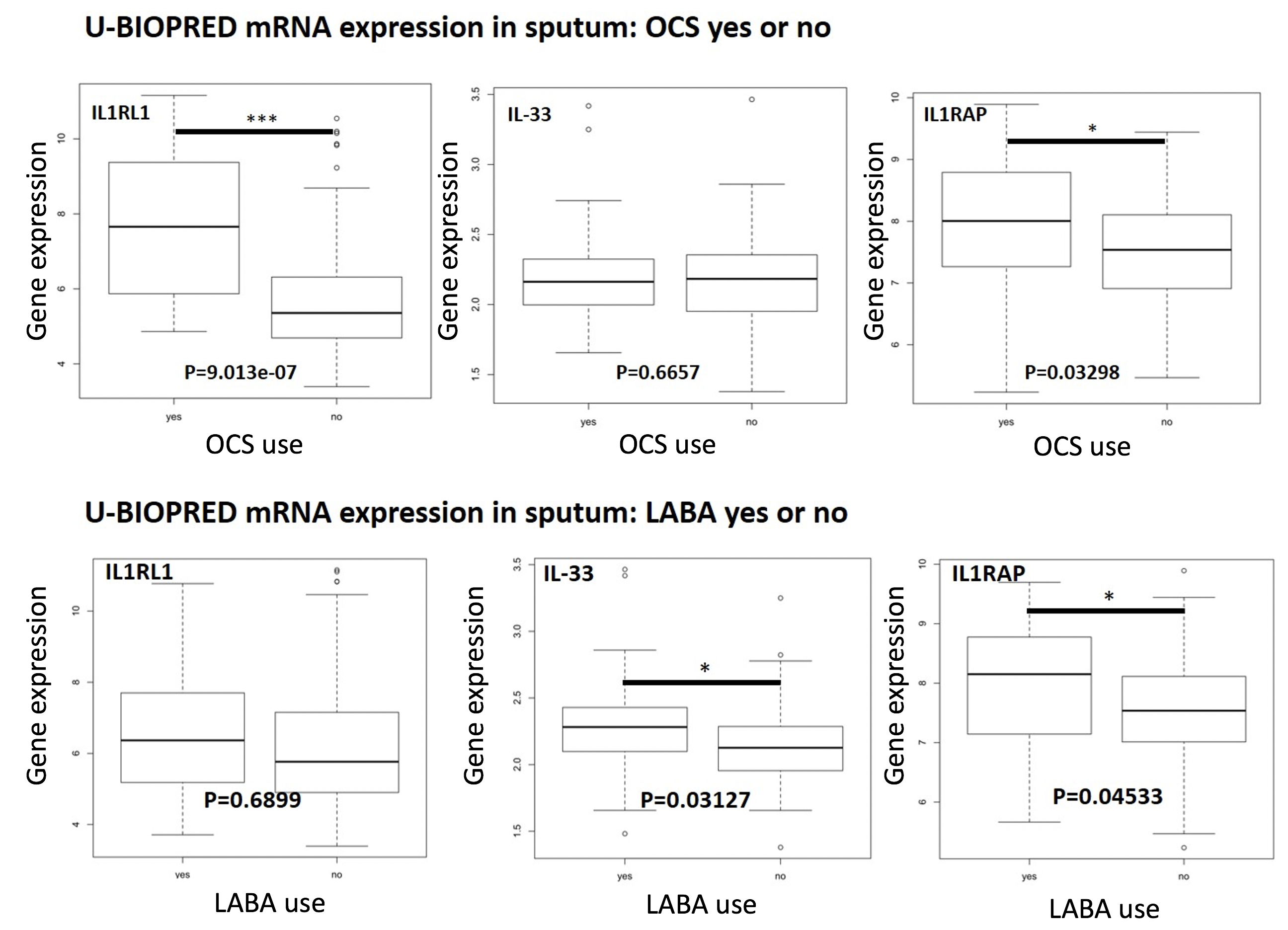


**Supplementary Figure S7. Effect of oral corticosteroid (OCS, A) and long acting beta agonist (LABA, B) treatment on IL1RL1, IL-33 and IL1RAP mRNA in sputum.**


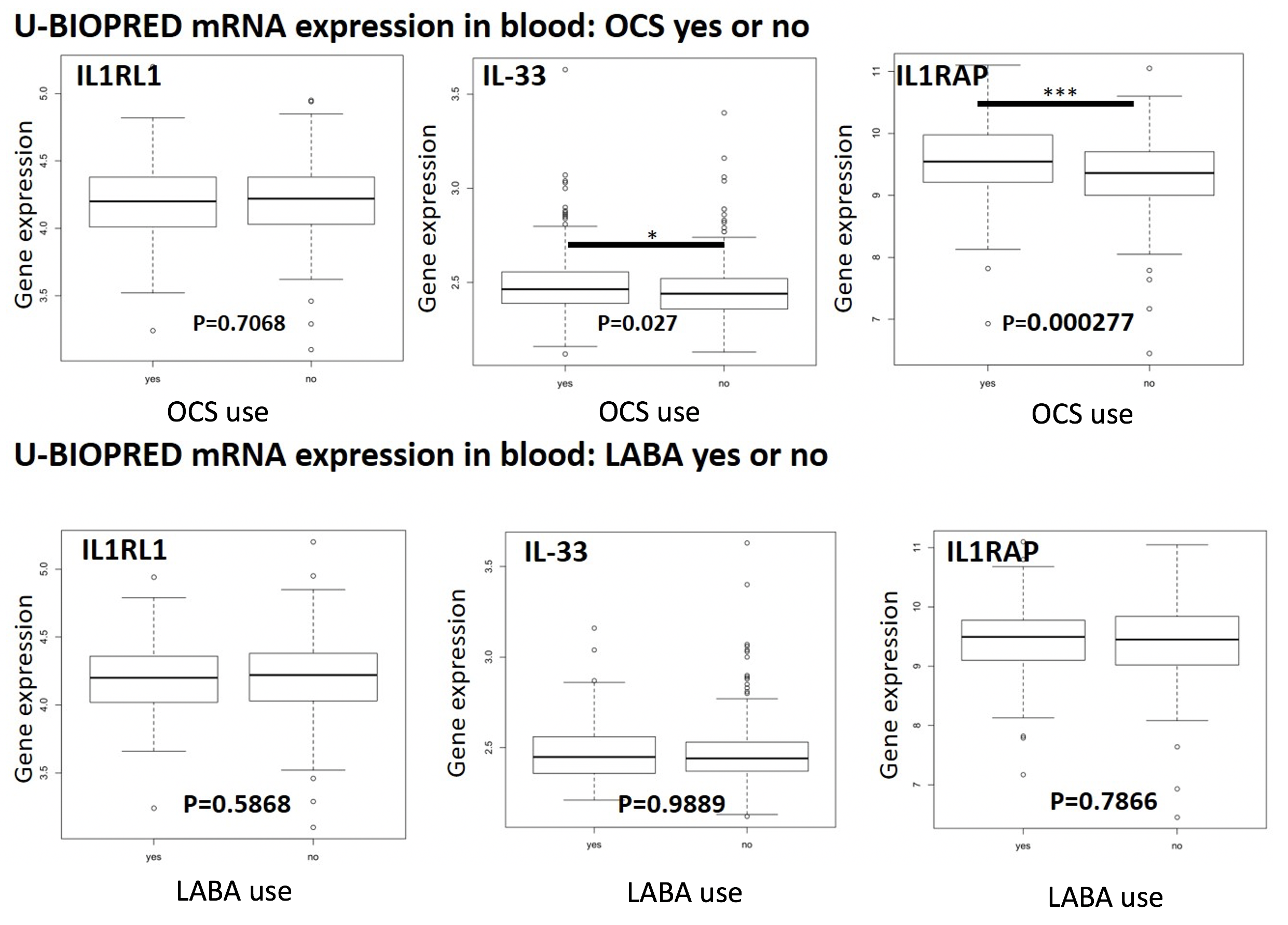


**Supplementary Figure S8. Effect of oral corticosteroid (OCS, A) and long acting beta agonist (LABA, B) treatment on IL1RL1, IL-33 and IL1RAP mRNA in blood.**


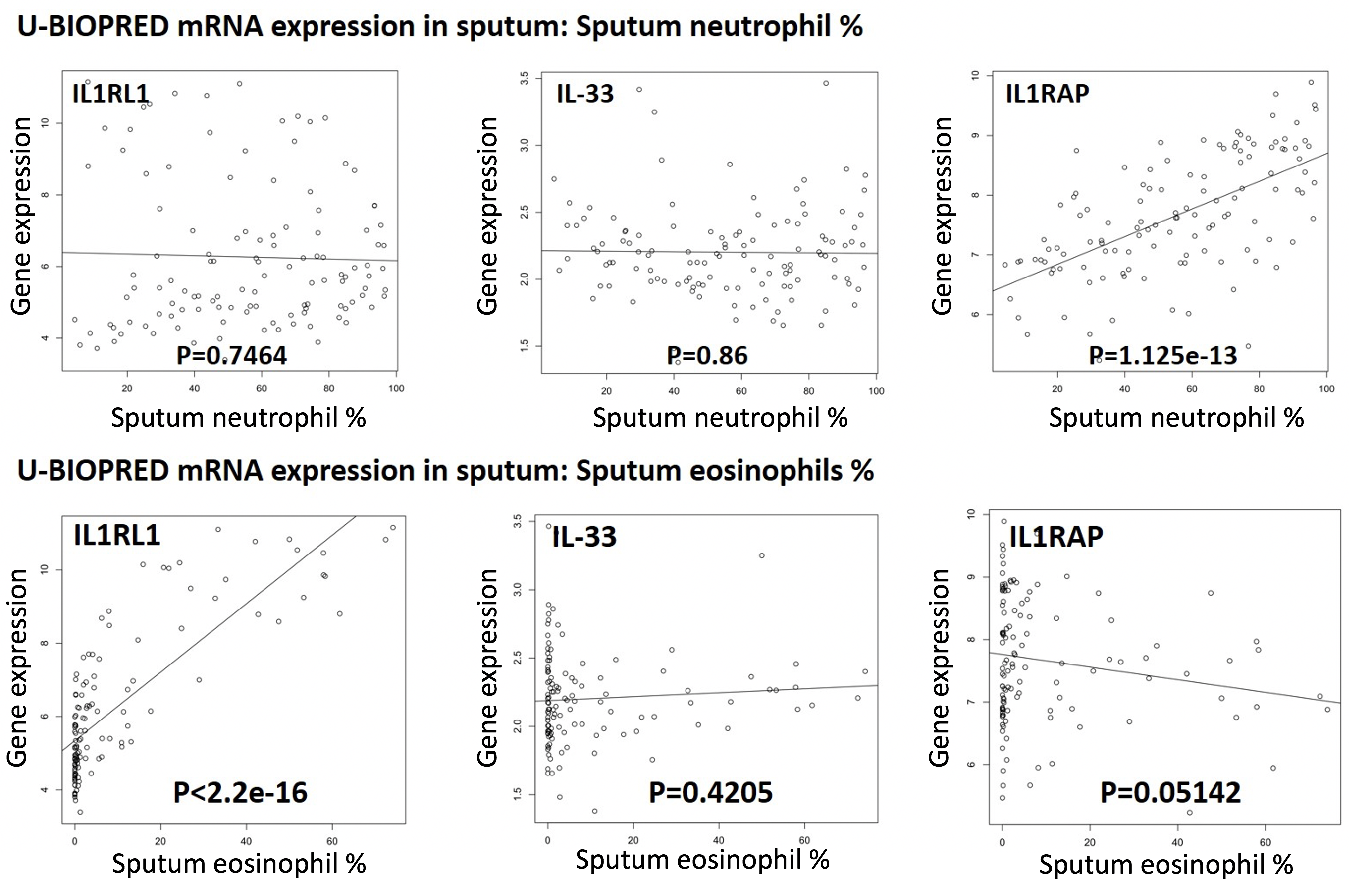


**Supplementary Figure S9.** Correlation between sputum neutrophil (A) and eosinophil levels with sputum IL1RL1, IL-33 and IL1RAP mRNA.


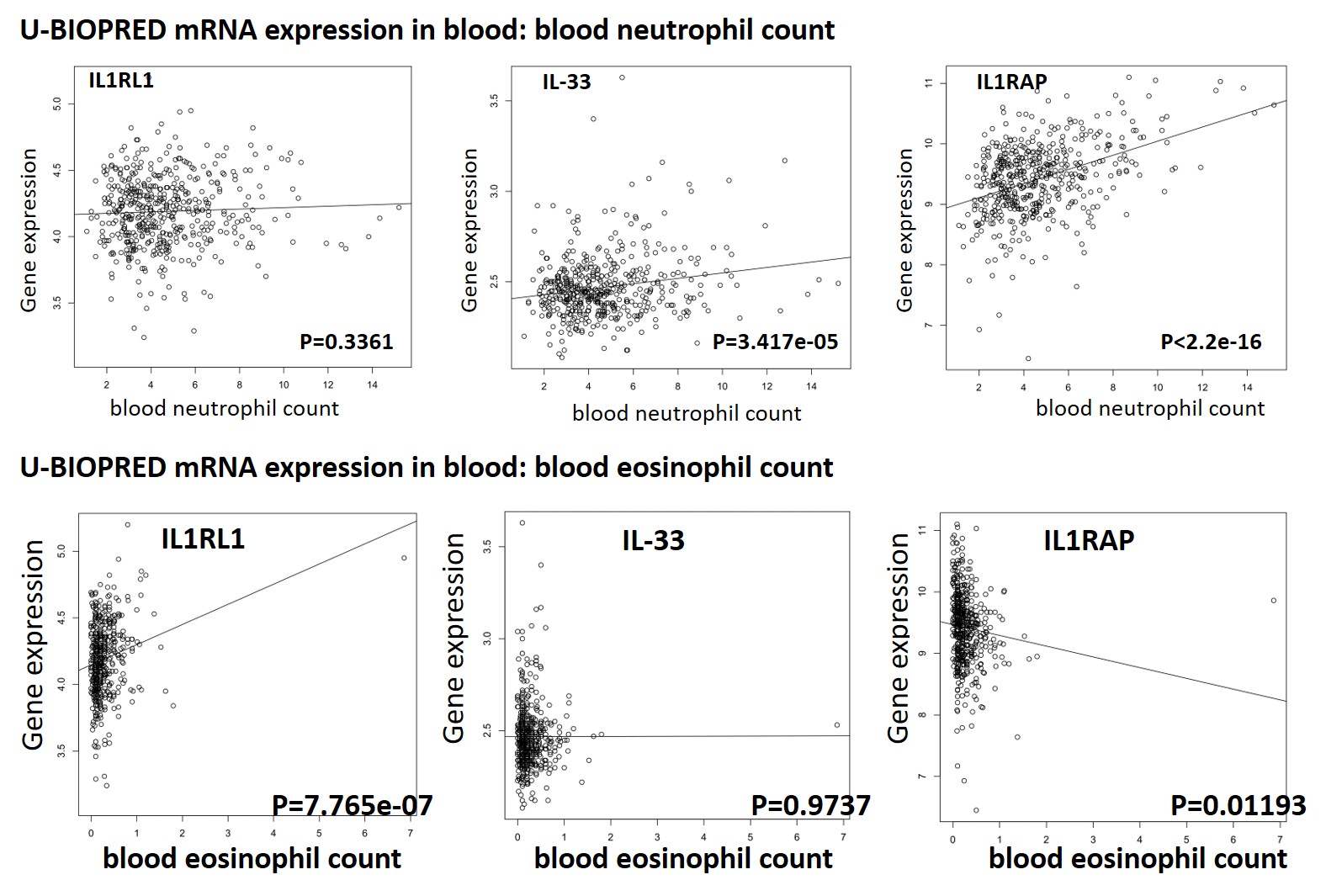


**Supplementary Figure S10.** Correlation between blood neutrophil (A) and eosinophil levels with blood IL1RL1, IL-33 and IL1RAP mRNA.


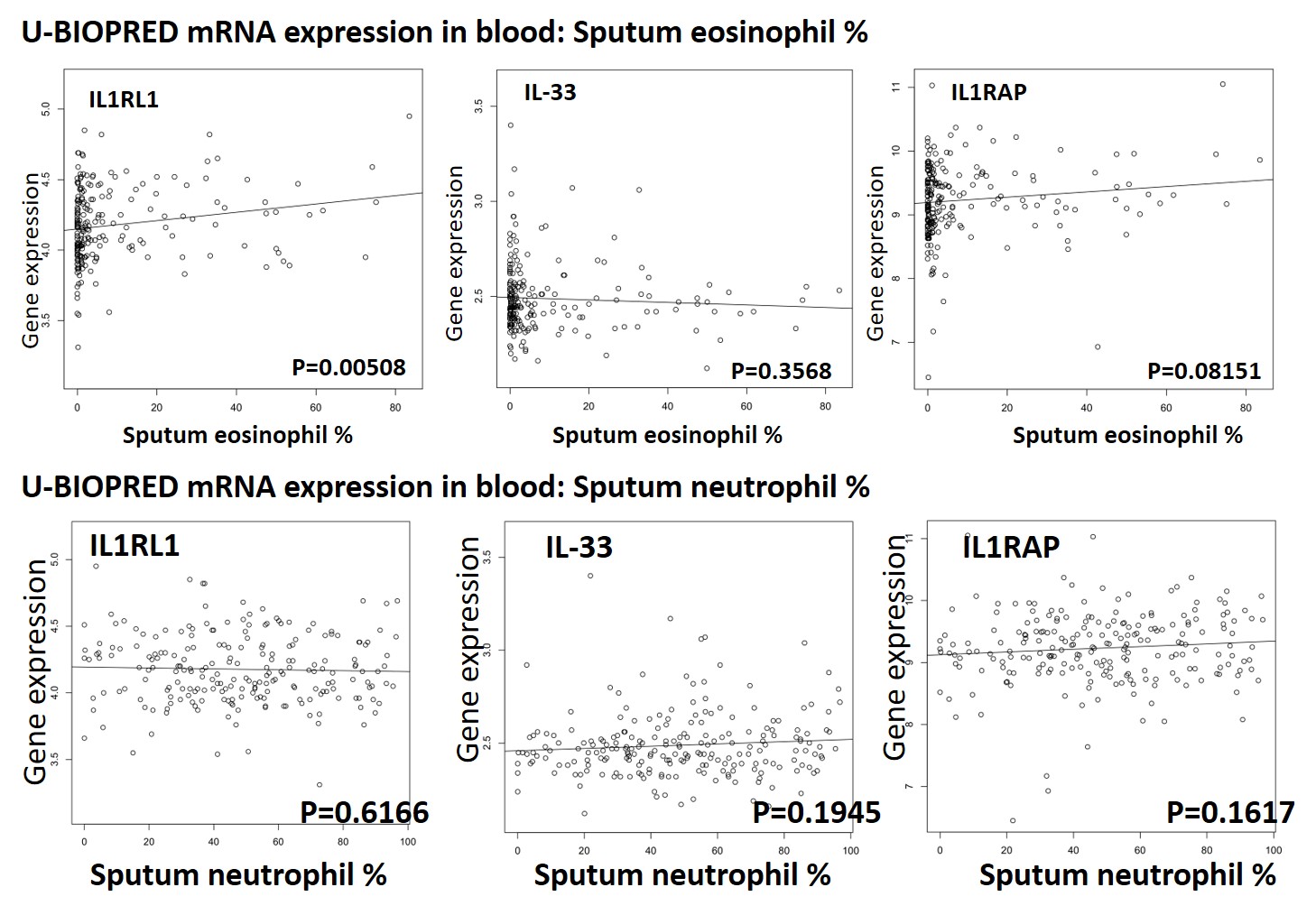


**Supplementary Figure S11.** Correlation between sputum neutrophil (A) and eosinophil levels with blood IL1RL1, IL-33 and IL1RAP mRNA.

**Supplementary References**

1. Bahri R, Custovic A, Korosec P, Tsoumani M, Barron M, Wu J, Sayers R, Weimann A, Ruiz-Garcia M, Patel N, Robb A, Shamji MH, Fontanella S, Silar M, Mills ENC, Simpson A, Turner PJ, Bulfone-Paus S. Mast cell activation test in the diagnosis of allergic disease and anaphylaxis. J Allergy Clin Immunol. 2018 Aug;142(2):485-496.e16. doi: 10.1016/j.jaci.2018.01.043. Epub 2018 Mar 5.
2. Vieira Braga FA, Kar G, Berg M, Carpaij OA, Polanski K, Simon LM, Brouwer S, Gomes T, Hesse L, Jiang J, Fasouli ES, Efremova M, Vento-Tormo R, Talavera-Lopez C, Jonker MR, Affleck K, Palit S, Strzelecka PM, Firth HV, Mahbubani KT, Cvejic A, Meyer KB, Saeb-Parsy K, Luinge M, Brandsma CA, Timens W, Angelidis I, Strunz M, Koppelman GH, van Oosterhout AJ, Schiller HB, Theis FJ, van den Berge M, Nawijn MC, Teichmann SA. A cellular census of human lungs identifies novel cell states in health and in asthma. *Nat Med* 2019; 25: 1153-1163. doi: 1110.1038/s41591-41019-40468-41595. Epub 42019 Jun 41517.
3. Abbas AR, Baldwin D, Ma Y, Ouyang W, Gurney A, Martin F, Fong S, van Lookeren Campagne M, Godowski P, Williams PM, Chan AC, Clark HF. Immune response in silico (IRIS): immune-specific genes identified from a compendium of microarray expression data. *Genes Immun* 2005; 6: 319-331. doi: 310.1038/sj.gene.6364173.
4. Sridhar S, Liu H, Pham TH, Damera G, Newbold P. Modulation of blood inflammatory markers by benralizumab in patients with eosinophilic airway diseases. *Respir Res* 2019; 20: 14. doi: 10.1186/s12931-12018-10968-12938.
5. MacGlashan D, Jr. Expression profiling of human basophils: modulation by cytokines and secretagogues. *PLoS One* 2015; 10: e0126435. doi: 0126410.0121371/journal.pone.0126435. eCollection 0122015.
6. Pollheimer J, Bodin J, Sundnes O, Edelmann RJ, Skånland SS, Sponheim J, Brox MJ, Sundlisaeter E, Loos T, Vatn M, Kasprzycka M, Wang J, Küchler AM, Taskén K, Haraldsen G, Hol J. Interleukin-33 drives a proinflammatory endothelial activation that selectively targets nonquiescent cells. *Arterioscler Thromb Vasc Biol* 2013; 33: e47-55. doi: 10.1161/ATVBAHA.1112.253427. Epub 252012 Nov 253415.
